# Cost thresholds for anticipated long‐acting HIV pre‐exposure prophylaxis products in Eastern and Southern Africa: a mathematical modelling study

**DOI:** 10.1002/jia2.26427

**Published:** 2025-02-24

**Authors:** David Kaftan, Monisha Sharma, Danielle Resar, Masabho Milali, Edinah Mudimu, Linxuan Wu, Cory Arrouzet, Ingrida Platais, Hae‐Young Kim, Sarah Jenkins, Anna Bershteyn

**Affiliations:** ^1^ Department of Population Health New York University Grossman School of Medicine New York New York 10016 USA; ^2^ Department of Global Health University of Washington Seattle Washington USA; ^3^ Clinton Health Access Initiative Boston Massachusetts USA; ^4^ University of South Africa Pretoria South Africa

**Keywords:** Africa, HIV, lenacapavir, long‐acting, modelling, pre‐exposure prophylaxis

## Abstract

**Introduction:**

Affordable HIV prevention tools are needed in Eastern and Southern Africa (ESA). Several promising long‐acting pre‐exposure prophylaxis (LA‐PrEP) products are available or in development. However, ESA settings face severe healthcare resource constraints. We aimed to estimate the threshold price at which LA‐PrEP products could be cost‐effective in three ESA settings.

**Methods:**

We adapted an agent‐based model, EMOD‐HIV, to simulate LA‐PrEP (monthly oral, 2‐ and 6‐monthly injectable) rollout in South Africa, Zimbabwe and Kenya. Due to uncertainties about LA‐PrEP use, we examined a range of coverages (5%−20% of HIV‐negative sexually active adults) and extents to which LA‐PrEP use will be concentrated among those most at risk (prioritized rollout from higher‐ to lower‐risk groups vs. uniform rollout among sexually active adults). To evaluate a 20‐year commitment to LA‐PrEP delivery, we assumed LA‐PrEP was scaled up to target coverage from 2025 to 2030 and maintained at target levels before ending in 2045. We estimated maximum per‐dose and per‐year LA‐PrEP costs that achieve cost‐effectiveness (<US$500 per disability‐adjusted life‐year averted) over 35 years (until 2060), compared to a scenario of daily oral PrEP only. Sensitivity analyses varied PrEP scale‐up speeds and eligible populations.

**Results:**

Risk‐prioritized LA‐PrEP for 5% of adults was projected to avert 11–21% of HIV acquisitions across settings, with 3–5 times more HIV acquisitions averted and 3–5 times higher maximum cost compared to non‐prioritized rollout. Six‐monthly injectable PrEP supported the highest per‐dose cost: in the scenario with the most cost‐effective LA‐PrEP use (5% risk‐prioritized rollout), the maximum per‐dose price in South Africa was $52.99 (95% CI: $48.82–$57.21), in Zimbabwe $14.64 (95% CI: $12.04–$17.38) and in western Kenya $7.50 (95% CI: $6.73–$8.27). For monthly oral PrEP, corresponding per‐dose costs were $5.02 (95% CI: $4.67–$5.37), $1.45 (95% CI: $1.10–$1.79) and $0.87 (95% CI: $0.80–$0.93). Results were sensitive to eligible population and prioritization, and moderately sensitive to scale‐up speed and product effectiveness.

**Conclusions:**

LA‐PrEP is likely to require reduced pricing and/or risk‐prioritized rollout to be cost‐effective in ESA.

## 1 INTRODUCTION

Despite substantial strides in increasing HIV treatment coverage, HIV incidence has not declined to levels of epidemic control in Eastern and Southern Africa (ESA) [1]. Oral pre‐exposure prophylaxis (PrEP) is effective at preventing HIV when taken as prescribed, but uptake and adherence remain low in ESA [2, 3]. Barriers to oral PrEP include pill burden and stigma associated with taking daily antiretrovirals. Studies show preferences for long‐acting PrEP (LA‐PrEP) products among both the general and key populations, including female sex workers, men who have sex with men, transgender people, and adolescent girls and young women [4, 5, 6]. Therefore, substantial research has focused on developing long‐acting PrEP, including 6‐monthly injectable lenacapavir, next‐generation analogues of the oral PrEP candidate islatravir and injectable 2‐monthly cabotegravir (CAB‐LA), which has been approved by the European Medicines Agency and regulatory authorities in 22 other countries as of November 2024 [7, 8].

CAB‐LA is an HIV integrase inhibitor demonstrated safe and effective in two Phase 3 clinical trials when administered using 2‐monthly intramuscular injections [7, 8]. The trials included sexual minority cisgender men and transgender women in the United States, Latin America, Asia, and Africa and heterosexual women in ESA. Lenacapavir is an HIV capsid inhibitor that reduces HIV risk by interfering with multiple stages of the HIV replication cycle [9, 10, 11]. It is administered through 6‐monthly subcutaneous injections and completed Phase 3 clinical trials in adolescent girls and young women in South Africa and sexual minority cisgender men, transgender women, and gender nonbinary individuals in Brazil, Peru and the United States [12, 13, 14]. Potent nucleoside reverse transcriptase translocation inhibitors are under investigation for potential use as monthly oral PrEP [15]. Large‐scale clinical trials of one such compound, islatravir, were stopped due to observed lymphopenia in trial participants [16−18].

Particularly, in low‐ and middle‐income settings, economic evaluations can inform policy decisions regarding the scale‐up of medical products since, even if a product is safe, effective and in demand, there is a risk that it could divert limited resources from more efficient uses, resulting in a net decrement in population health. Resources are especially limited in ESA, home to two‐thirds of people living with HIV. Therefore, we evaluated the potential health impact and cost‐effectiveness of LA‐PrEP in three countries in ESA that were early adopters of oral PrEP: Kenya, South Africa and Zimbabwe. Since product costs are not yet known, we estimated the threshold per‐dose cost at which each product could be cost‐effective in different populations by country. Our analysis can help inform pricing, policy and research on LA‐PrEP in ESA [13, 19].

## 2 METHODS

We adapted an existing model, EMOD‐HIV, to simulate LA‐PrEP scale‐up. EMOD‐HIV is an open‐source HIV model integrating population demography, HIV disease progression and heterosexual network‐based HIV transmission, configured to match age‐ and sex‐specific propensities of forming different types of sexual partnerships [20, 21]. The model simulates within‐host disease processes and between‐host sexual interactions to reflect HIV transmission and the impact of interventions (e.g. antiretroviral therapy [ART] and PrEP) on population health. HIV interventions such as PrEP are incorporated via a configurable healthcare module, which includes a continuum of care and prevention with testing, linkage, retention, adherence and re‐engagement [22]. The model tracks health outcomes including HIV disease status, HIV‐related deaths and healthcare use, enabling the calculation of disability‐adjusted life years (DALYs) and HIV‐related costs.

EMOD‐HIV was parameterized with epidemiological data from South Africa (2020 adult HIV prevalence: 19.1%), western Kenya (11.3%) and Zimbabwe (11.9%) [23]. Model input data included fertility, mortality, voluntary male circumcision coverage, and the use of ART and oral PrEP. Model inputs lacking robust empiric data (particularly behavioural parameters such as multiple partnerships and condom usage) were adjusted to calibrate the model to estimates of HIV prevalence and incidence (Appendix S1). Our calibration began with previously published models for western Kenya [24] and South Africa [25]. We updated the calibrations using the latest epidemiological data from each country, including HIV prevalence and incidence by age and sex using population‐based HIV surveillance, numbers of individuals using ART and PrEP based on delivery data from Ministries of Health, population size and age/sex structure from the national census, and sizes of key populations based on enumeration studies. We selected 100 model parameter sets using roulette resampling in proportion to the goodness‐of‐fit of each simulation to the calibration data. Quantiles of resulting model runs were used to generate confidence intervals for estimates reported, which can be conceptualized as representing a combination of parameter and stochastic uncertainty.

### 2.1 Modelled scenarios

We simulated a reference scenario of continuation of oral PrEP use at 2022 levels and no LA‐PrEP availability. Data on oral PrEP use in each setting were obtained from PrEPWatch [26]. We assumed oral PrEP had a 58% adherence‐adjusted effectiveness [27] and was used for 3 months, based on real‐world continuation data [28, 29]. In the intervention scenarios, we evaluated three LA‐PrEP products: (1) monthly oral PrEP analogous to the investigational antiretroviral MK‐8527; (2) injectable 2‐monthly LA‐PrEP analogous to currently licensed cabotegravir (CAB‐LA); and (3) injectable 6‐monthly PrEP analogous to lenacapavir (LEN). Each LA‐PrEP product was modelled separately as health systems’ capacity to offer multiple products simultaneously is not well‐understood and beyond the scope of the present study.

All LA‐PrEP products were assumed to have high efficacy in base case analyses (Table 1). Based on HPTN 084 (CAB‐LA demonstrated a 0.12 hazard ratio compared to daily oral tenofovir/emtricitabine) [7] and PURPOSE 1 (lenacapavir demonstrated 100% efficacy) [30] trials, we assumed that injectable PrEP decreased the risk of HIV acquisition by 95% for the duration of protection (2 or 6 months) with no protection beyond this time. Similar to oral PrEP, we assumed monthly oral PrEP has high efficacy when used with high adherence. However, we assume users take two out of every three pills, yielding a real‐world effectiveness of 66%.

**Table 1:** Key model parameters

Parameter	Value	Source
CAB‐LA effectiveness	95%	Landovitz et al. [8]; Delany‐Moretlwe et al. [7]
LEN effectiveness	95%	Gilead [30]
Monthly oral effectiveness	66%	Assumption
Oral PrEP effectiveness	58%	Delany‐Moretlwe et al. [7]
Two‐monthly eligibility re‐assessment (CAB‐LA)	2 monthly	FDA [47]
Six‐monthly eligibility (LEN) re‐assessment	6 monthly	Gilead Sciences [12]
Monthly oral PrEP eligibility re‐assessment	3 monthly	Merck Sharp & Dohme [17]
LA‐PrEP scale‐up period	2030−2035	Assumption
LA‐PrEP implementation ends	2045	Assumption
2020 HIV prevalence (ages 15–49)		
Western Kenya	11.3%	UNAIDS [23]
Zimbabwe	11.9%	UNAIDS [23]
South Africa	19.1%	UNAIDS [23]
Population % female		
Western Kenya	52%	UN [48]
Zimbabwe	52%	UN [48]
South Africa	50%	UN [48]
Age: median and interquartile range (IQR)		
Western Kenya	18 (IQR: 8–31)	UN [48]
Zimbabwe	20 (IQR: 9–35)	UN [48]
South Africa	27 (IQR: 13–42)	UN [48]

*Note*: CAB, LEN and LA‐PrEP refer to cabotegravir, lenacapavir and long‐acting pre‐exposure prophylaxis, respectively. Costs are listed in 2021 USD. HIV prevalence was used in HIV calibration and incidence was used in validation. HIV incidence (ages 15–49) across settings: Kenya (2018): 0.15%, Zimbabwe (2020): 0.45%, South Africa (2017): 0.79% (see Supplementary Appendix for more information).

We assumed LA‐PrEP scaled linearly to the target coverage from 2025 to 2030 and remained at the target until 2045. We assumed coverage ends in 2045 in order to evaluate the cost‐effectiveness of a 20‐year commitment to LA‐PrEP delivery. We varied the final proportion of adults receiving LA‐PrEP by 2030 from 5% to 20% across scenarios. Individuals aged 18–49 years were eligible for LA‐PrEP if they were HIV negative at the time of initiation/continuation, and sexually active with at least one partner. We assumed LA‐PrEP discontinuation occurred after product duration ends or after eligibility criteria were no longer met (i.e. if an individual turned 50 years old, has no sexual partners or tested HIV positive). Individuals who discontinued LA‐PrEP were eligible to re‐initiate at background rates.

Due to uncertainty regarding which populations might access LA‐PrEP products, we conducted a bounding analysis varying the extent to which LA‐PrEP was used by those most‐at‐risk of HIV. At one extreme, LA‐PrEP was allocated to those at the highest risk (female sex workers) and then progressively to the groups at the next‐highest risk until the pre‐specified overall adult LA‐PrEP coverage was reached. At the opposite extreme, LA‐PrEP was allocated equally among all sexually active adults.

### 2.2 Outcomes

For each scenario, we assessed number of new HIV acquisitions, HIV‐related deaths, DALYs and net cost impact of PrEP provision on HIV programme spending, including avoided ART costs, over a 35‐year time horizon (until 2060). We estimated the percentage of HIV acquisitions and HIV‐related deaths averted among 18‐ to 49‐year‐olds compared to the counterfactual scenario of daily oral PrEP only. We calculated 95% credible intervals (CI) across the 100 parameter sets to assess parameter uncertainty. We also assessed the potential for each LA‐PrEP product to achieve UNAIDS HIV incidence targets of 1 per 1000 person‐years by 2040 [31].

### 2.3 Cost threshold analysis

We assessed the maximum cost per dose (fully loaded cost including commodities and delivery costs) at which each LA‐PrEP product would be cost‐effective in each scenario. We utilized a commonly cited HIV service cost‐effectiveness threshold of $500 USD per DALY averted [32, 33, 34]. We compared each LA‐PrEP scenario to a reference scenario of daily oral PrEP only. We did not include the costs of identifying and reaching different subgroups. We estimated maximum LA‐PrEP costs inclusive of drug, supply chain, clinic visits and HIV testing. Analysis of model outputs was conducted in R version 4.0.3.

### 2.4 Sensitivity analyses

In sensitivity analysis, we varied the duration of the LA‐PrEP scale‐up from 5 years (2025–2030, in the base case) to 10 years (2025–2035). We also varied the final proportion of the adult population receiving PrEP by 2030 from 5% to 20%. Further, for scenarios of 5% coverage in each country (for both risk‐prioritized and non‐prioritized scale‐up), we varied the effectiveness of each PrEP product based on confidence intervals from clinical trials or prior modelling studies: CAB‐LA (76%, 97%), LEN (100%), monthly oral PrEP (56%, 77%) [35, 36]. We did not model an additional lower bound for LEN, since our base case estimate is close to the lower estimate observed in clinical trials. Finally, since clinical trials among heterosexual adults in ESA have focused on women, we evaluated the impact of scale‐up to females only (instead of all adults in the base case).

### 2.5 Ethics and consent

Data used for this analysis were from publicly available sources, therefore, did not require informed consent nor ethical approval.

## 3 RESULTS

### 3.1 HIV acquisitions averted by LA‐PrEP

Across settings, risk‐prioritized LA‐PrEP scale‐up was estimated to avert 3–5 times as many HIV acquisitions as non‐prioritized scale‐up to the general population at the same coverage levels (Table 2). In the scenario that assumed the most efficient use of PrEP (5% risk‐prioritized coverage of adults by 2030), monthly oral PrEP was estimated to avert 13% of HIV acquisitions in western Kenya, 12% in Zimbabwe and 12% in South Africa compared to the reference scenario of daily oral PrEP only. With the same coverage and targeting, 2‐ or 6‐monthly injectable PrEP were projected to avert 19% of acquisitions in western Kenya, 20% in Zimbabwe and 22% in South Africa.

**Table 2:** Costs and health outcomes of LA‐PrEP interventions by country assuming 5% LA‐PrEP scale up to all adults starting in 2030^a^

Population	Product	% of infections averted	% of deaths averted	Person years on PrEP (millions)	Person years on LA PrEP per DALY averted	Max. price per dose ($)	Max. price per person year on LA PrEP ($)
Western Kenya							
Risk‐prioritized	MO	12.95 (12.23, 13.67)	2.66 (2.38, 2.94)	3.39 (3.38−3.39)	66 (62, 72)	0.87 (0.8, 0.93)	10.43 (9.65, 11.17)
	CAB	18.72 (18.02, 19.43)	3.79 (3.43, 4.14)	3.32 (3.32−3.33)	46 (41, 53)	2.48 (2.16, 2.79)	14.91 (12.97, 16.73)
	LEN	18.74 (18.14, 19.37)	3.99 (3.62, 4.36)	3.45 (3.45−3.46)	46 (42, 51)	7.5 (6.73, 8.27)	15.0 (13.47, 16.54)
Non‐prioritized	MO	4.25 (3.57, 4.91)	0.91 (0.6, 1.23)	3.31 (3.31−3.32)	203 (167, 260)	0.29 (0.22, 0.35)	3.42 (2.67, 4.17)
	CAB	6.31 (5.56, 7.07)	1.3 (0.95, 1.66)	3.30 (3.30−3.31)	146 (105, 250)	0.79 (0.48, 1.11)	4.76 (2.86, 6.67)
	LEN	5.95 (5.21, 6.69)	1.26 (0.86, 1.67)	3.31 (3.31−3.32)	137 (104, 198)	2.53 (1.69, 3.3)	5.05 (3.38, 6.61)
Zimbabwe							
Risk‐prioritized	MO	12.26 (9.4, 15.05)	4.38 (3.26, 5.46)	7.60 (7.58−7.61)	34 (27, 44)	1.45 (1.1, 1.79)	17.35 (13.19, 21.51)
	CAB	19.86 (16.9, 22.79)	7.17 (6.07, 8.3)	7.52 (7.50−7.54)	21 (17, 25)	4.72 (3.86, 5.6)	28.30 (23.14, 33.60)
	LEN	20.97 (18.18, 23.83)	7.58 (6.4, 8.83)	7.68 (7.66−7.70)	20 (16, 25)	14.64 (12.04, 17.38)	29.29 (24.08, 34.75)
Non‐prioritized	MO	4.16 (1.25, 7.03)	1.94 (0.91, 2.97)	7.50 (7.47−7.52)	78 (51, 176)	0.63 (0.29, 0.95)	7.52 (3.48, 11.38)
	CAB	7.34 (4.23, 10.44)	2.68 (1.5, 3.79)	7.48 (7.46−7.50)	50 (35, 93)	1.95 (1.06, 2.81)	11.68 (6.37, 16.86)
	LEN	6.74 (3.43, 9.9)	3.07 (1.89, 4.23)	7.50 (7.48−7.52)	52 (35, 101)	5.73 (2.97, 8.37)	11.46 (5.94, 16.73)
South Africa							
Risk‐prioritized	MO	11.87 (11.1, 12.58)	4.87 (4.52, 5.23)	27.32 (27.22−27.43)	11 (10, 12)	5.02 (4.67, 5.37)	60.19 (56.03, 64.4)
	CAB	21.85 (20.97, 22.7)	8.88 (8.4, 9.37)	27.71 (27.57−27.85)	6 (5, 6)	18.28 (16.86, 19.67)	109.68 (101.16, 117.99)
	LEN	22.07 (21.3, 22.85)	8.83 (8.28, 9.38)	28.51 (28.39−28.63)	6 (6, 7)	52.99 (48.82, 57.21)	105.99 (97.64, 114.42)
Non‐prioritized	MO	2.73 (2.0, 3.43)	0.91 (0.59, 1.24)	25.68 (25.61−25.75)	47 (37, 65)	1.19 (0.87, 1.51)	14.29 (10.46, 18.18)
	CAB	5.48 (4.65, 6.32)	2.1 (1.62, 2.59)	25.71 (25.64−25.78)	21 (16, 28)	5.39 (3.93, 6.83)	32.33 (23.58, 40.96)
	LEN	4.4 (3.65, 5.19)	1.71 (1.17, 2.25)	25.70 (25.63−25.77)	25 (18, 37)	13.69 (8.89, 18.44)	27.37 (17.78, 36.89)

*Note*: MO, CAB and LEN refer to monthly oral, cabotegravir and lenacapavir, respectively. Health impacts are compared to baseline scenario of daily oral PrEP only over a 35‐year time horizon. Prices are in 2021 USD. Values in parentheses show 95% uncertainty intervals representing the 2.5th and 97.5th percentiles across 100 parameter sets.

In risk‐prioritized scenarios, there were no significant differences in health impact between the 2‐ versus 6‐monthly injectable PrEP. However, in non‐prioritized LA‐PrEP scenarios, 2‐monthly injectable PrEP (CAB‐LA) averted slightly more HIV acquisitions than 6‐monthly injectable PrEP (e.g. 7.3% vs. 6.7% in Zimbabwe at 5% coverage) likely due to more re‐assessment of individuals’ risk due to more frequent dosing (Table 2).

The largest number of HIV acquisitions averted over 2025–2045 by LA‐PrEP, in absolute terms and per person‐year of PrEP provided, occurred in South Africa due to its relatively high HIV prevalence and incidence (Table S1). However, the proportion of total HIV acquisitions averted by LA‐PrEP was lower in South Africa than in Zimbabwe and western Kenya in the scenario where only the highest‐risk 5% of the population received LA‐PrEP.

### 3.2 DALYs averted by LA‐PrEP

Person‐years on LA‐PrEP per DALY averted were 2.5−4 times higher in the non‐prioritized than risk‐prioritized scenarios and 1.5 times higher for monthly oral PrEP compared to either 2‐ or 6‐monthly injectable PrEP in the same setting and population. Person‐years on PrEP to avert one DALY were lowest in South Africa since it had the highest HIV incidence and largest gaps in the HIV care continuum among the three settings. For example, risk‐prioritized 6‐monthly injectable PrEP required an estimated 6 person‐years to avert one DALY in South Africa versus 46 person‐years in western Kenya (Table 2).

### 3.3 Maximum cost per dose

The maximum cost per dose for LA‐PrEP to be cost‐effective (Table 2) was highest for 6‐monthly injectable PrEP, which requires the fewest annual doses, and lowest for monthly oral PrEP. Similar to per‐year costs, the maximum cost per dose was highest in South Africa and higher in risk‐prioritized scenarios. At 5% risk‐prioritized coverage, the maximum per‐dose cost of 6‐monthly PrEP scenarios was $7.50 (95% CI: 6.73−8.27) in western Kenya, $14.64 (95% CI: 12.04−17.38) in Zimbabwe and $52.99 (95% CI: 48.82−57.21) in South Africa. The maximum per‐dose cost of monthly oral PrEP was $0.87 (95% CI: 0.80−0.93) in western Kenya, $1.45 (95% CI: 1.10, 1.79) in Zimbabwe and $5.02 (95% CI: 4.67−5.37) in South Africa.

### 3.4 Maximum cost per person‐year

The maximum cost per person‐year on LA‐PrEP (Table 2) was lowest for monthly oral PrEP across settings and similar for 2‐ and 6‐monthly injectable PrEP when delivered with the same coverage and targeting. Maximum costs were highest in South Africa and lowest in western Kenya. Risk‐prioritized LA‐PrEP scale‐up in South Africa resulted in a maximum cost per year on LA‐PrEP of $109.68 (95% CI: 101.16−117.99) per year for 2‐monthly injectable PrEP, $105.99 (95% CI: 97.64−114.42) for 6‐monthly injectable and $60.19 (95% CI: 56.03−64.4) for monthly oral. In Kenya, risk‐prioritized 2‐monthly injectable PrEP could cost up to $15.00 (95% CI: 13.47−16.54) per year, 6‐monthly injectable PrEP could cost up to $14.91 (95% CI: 12.97−16.73) per year, while monthly oral PrEP could cost up to $10.43 (95% CI: 9.65−11.17) per year. In Zimbabwe, risk‐prioritized 2‐monthly injectable PrEP could cost up to $28.30 (95% CI: 23.14−33.6) per year, 6‐monthly injectable PrEP could cost up to $29.29 (95% CI: 24.08−34.75) per year, while monthly oral PrEP could cost up to $17.35 (95% CI: 13.19−21.51) per year.

### 3.5 Effect of PrEP on time to reach HIV incidence targets

**Figure 1:**
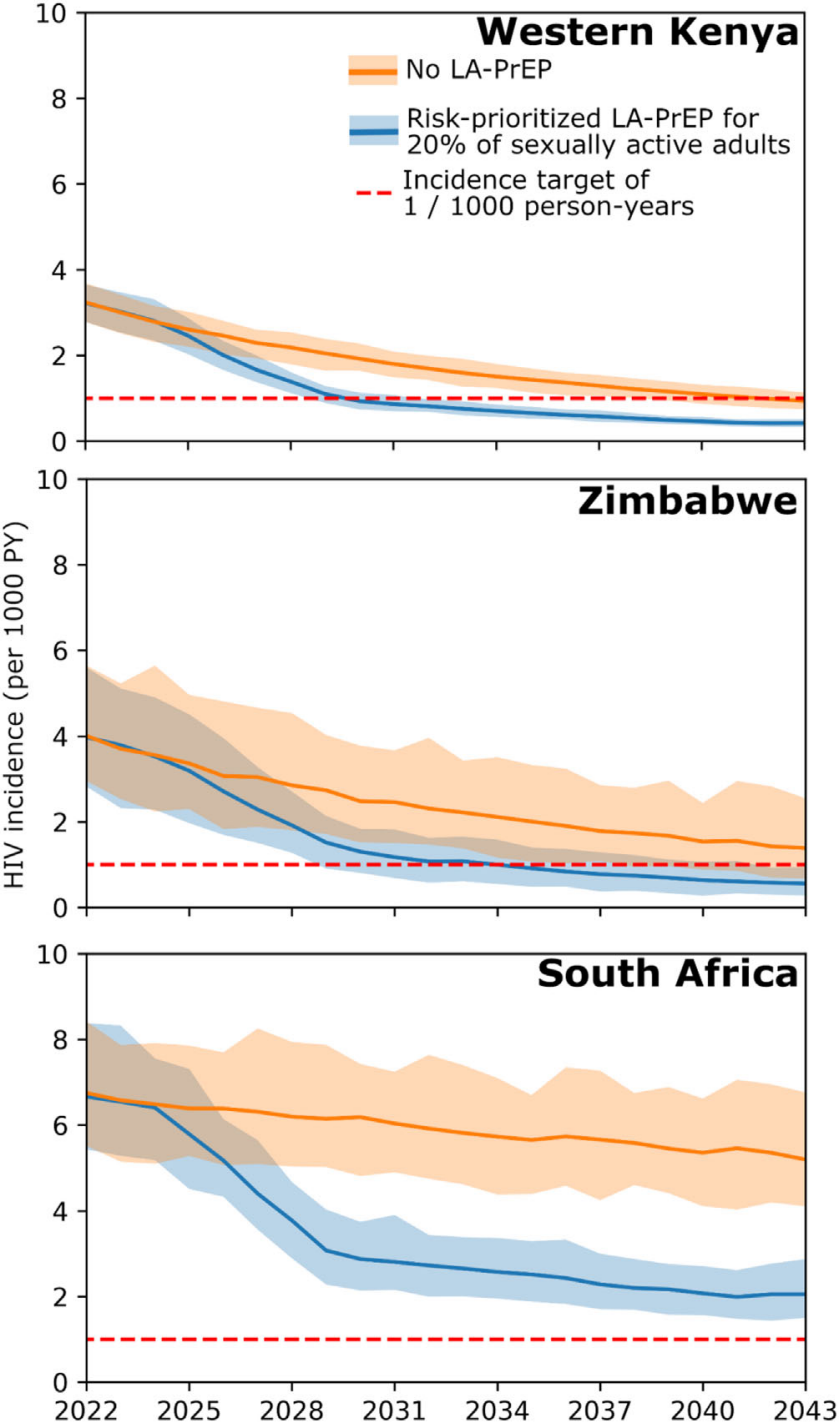
Impact of 20% coverage of risk‐prioritized 2‐monthly LA‐PrEP (blue) compared to no LA‐PrEP (orange) on HIV incidence over 2023–2043 in Nyanza (top panel), Zimbabwe (middle panel) and South Africa (bottom panel). With LA‐PrEP, an incidence target of 1 per 1000 person‐years is reached in Nyanza by 2030 and in Zimbabwe by 2034. This target is not met in South Africa by 2043.

Scale‐up of risk‐prioritized LA‐PrEP could allow western Kenya and Zimbabwe to reach HIV incidence targets of <0.1% by 2040 [31], but targets were not reached for South Africa (Figure 1). In western Kenya, targets were reached with all three products by 2040 with 5% coverage, but the time to reach targets was shorter for injectable LA‐PrEP (reached by 2037) compared to monthly oral PrEP (reached by 2039). In Zimbabwe, incidence targets were met with 10% coverage injectable LA‐PrEP but only reached with 20% coverage of monthly oral PrEP.

### 3.6 Sensitivity to PrEP coverage

The percentage of HIV acquisitions averted increased with expanding LA‐PrEP coverage, but with diminishing returns, for example doubling the coverage of LA‐PrEP did not double the percentage reduction in HIV incidence (Table 3A). Some strategies halved new HIV acquisitions over 2025–2045. Percentage HIV acquisition reductions were similar for western Kenya and Zimbabwe and lower for South Africa, although the absolute number of HIV acquisitions, deaths and DALYs averted was highest in South Africa (Appendix S2).

**Table 3:** HIV infections averted and maximum annual price thresholds for LA‐PrEP assuming PrEP scale up among all adults starting in 2030^a^

A. Percent of all HIV infections averted over 2025–2045 at varying LA‐PrEP coverage levels					
Population	product	5%	10%	15%	20%
Western Kenya					
Risk‐prioritized	MO	12.95 (12.23, 13.67)	20.55 (19.91, 21.19)	25.16 (24.61, 25.7)	28.55 (27.94, 29.14)
	CAB	18.72 (18.02, 19.43)	29.33 (28.72, 29.92)	36.25 (35.69, 36.8)	40.96 (40.45, 41.47)
	LEN	18.74 (18.14, 19.37)	28.96 (28.32, 29.6)	35.39 (34.86, 35.92)	40.6 (40.11, 41.09)
Non‐prioritized	MO	4.25 (3.57, 4.91)	8.43 (7.71, 9.17)	12.6 (11.93, 13.27)	16.19 (15.6, 16.78)
	CAB	6.31 (5.56, 7.07)	12.33 (11.54, 13.08)	18.57 (17.8, 19.3)	23.24 (22.6, 23.87)
	LEN	5.95 (5.21, 6.69)	12.44 (11.74, 13.13)	18.59 (17.95, 19.2)	24.13 (23.5, 24.73)
Zimbabwe					
Risk‐prioritized	MO	12.26 (9.4, 15.05)	22.97 (20.35, 25.44)	25.55 (23.37, 27.61)	27.67 (25.55, 29.85)
	CAB	19.86 (16.9, 22.79)	33.87 (31.61, 36.19)	37.72 (35.64, 39.86)	40.71 (38.52, 42.9)
	LEN	20.97 (18.18, 23.83)	33.48 (31.1, 35.75)	36.6 (34.29, 38.9)	40.5 (38.67, 42.25)
Non‐prioritized	MO	4.16 (1.25, 7.03)	7.66 (4.49, 10.81)	10.21 (7.14, 12.96)	16.05 (13.54, 18.41)
	CAB	7.34 (4.23, 10.44)	13.1 (9.7, 16.66)	18.75 (16.2, 21.3)	21.95 (18.91, 24.85)
	LEN	6.74 (3.43, 9.9)	14.05 (11.08, 16.87)	18.76 (15.73, 21.7)	23.02 (20.45, 25.55)
South Africa					
Risk‐prioritized	MO	11.87 (11.1, 12.58)	16.83 (16.1, 17.61)	20.14 (19.41, 20.87)	22.49 (21.78, 23.2)
	CAB	21.85 (20.97, 22.7)	27.35 (26.55, 28.14)	32.21 (31.58, 32.86)	35.22 (34.48, 35.93)
	LEN	22.07 (21.3, 22.85)	27.41 (26.74, 28.06)	32.3 (31.53, 33.06)	35.32 (34.63, 36.03)
Non‐prioritized	MO	2.73 (2.0, 3.43)	5.11 (4.28, 5.91)	8.23 (7.57, 8.9)	10.36 (9.74, 10.98)
	CAB	5.48 (4.65, 6.32)	9.73 (8.94, 10.49)	13.95 (13.16, 14.72)	18.22 (17.39, 19.02)
	LEN	4.4 (3.65, 5.19)	8.86 (8.06, 9.66)	13.97 (13.21, 14.72)	18.04 (17.36, 18.73)

B. Maximum price per person year at varying LA‐PrEP coverage levels					
Population	product	5%	10%	15%	20%
Western Kenya					
Risk‐prioritized	MO	$10.43 ($9.65, $11.17)	$9.20 ($8.81, $9.59)	$8.06 ($7.75, $8.38)	$7.62 ($7.37, $7.88)
	CAB	$14.91 ($12.97, $16.73)	$12.93 ($11.98, $13.86)	$11.77 ($11.1, $12.45)	$11.27 ($10.65, $11.89)
	LEN	$15.0 ($13.47, $16.54)	$12.64 ($11.8, $13.48)	$10.86 ($10.24, $11.47)	$10.42 ($9.94, $10.91)
Non‐prioritized	MO	$3.42 ($2.67, $4.17)	$3.57 ($3.24, $3.93)	$3.57 ($3.3, $3.84)	$3.53 ($3.34, $3.72)
	CAB	$4.76 ($2.86, $6.67)	$5.07 ($4.13, $6.01)	$5.05 ($4.41, $5.67)	$4.85 ($4.38, $5.33)
	LEN	$5.05 ($3.38, $6.61)	$5.37 ($4.46, $6.24)	$5.24 ($4.71, $5.8)	$5.12 ($4.72, $5.52)
Zimbabwe					
Risk‐prioritized	MO	$17.35 ($13.19, $21.51)	$16.34 ($14.43, $18.38)	$12.78 ($11.47, $14.19)	$10.68 ($9.68, $11.72)
	CAB	$28.3 ($23.14, $33.6)	$25.1 ($22.24, $27.99)	$19.34 ($17.57, $21.32)	$16.19 ($14.66, $17.8)
	LEN	$29.29 ($24.08, $34.75)	$24.35 ($21.65, $27.12)	$18.9 ($17.03, $20.81)	$15.87 ($14.5, $17.36)
Non‐prioritized	MO	$7.52 ($3.48, $11.38)	$6.7 ($4.61, $8.87)	$5.07 ($3.67, $6.44)	$6.04 ($5.14, $6.94)
	CAB	$11.68 ($6.37, $16.86)	$9.78 ($6.91, $12.73)	$9.23 ($7.58, $10.97)	$8.51 ($7.2, $9.9)
	LEN	$11.46 ($5.94, $16.73)	$10.87 ($8.16, $13.74)	$9.67 ($7.87, $11.48)	$8.92 ($7.63, $10.25)
South Africa					
Risk‐prioritized	MO	$60.19 ($56.03, $64.4)	$43.07 ($40.69, $45.46)	$34.68 ($33.23, $36.17)	$29.72 ($28.49, $30.95)
	CAB	$109.68 ($101.16, $117.99)	$72.15 ($67.47, $76.77)	$56.09 ($53.05, $59.23)	$47.31 ($44.95, $49.67)
	LEN	$105.99 ($97.64, $114.42)	$70.26 ($65.87, $74.66)	$54.15 ($51.09, $57.32)	$45.91 ($43.58, $48.29)
Non‐prioritized	MO	$14.29 ($10.46, $18.18)	$11.99 ($9.76, $14.15)	$13.42 ($12.13, $14.7)	$12.87 ($11.87, $13.9)
	CAB	$32.33 ($23.58, $40.96)	$27.24 ($22.78, $31.65)	$25.13 ($22.16, $28.11)	$24.14 ($21.82, $26.45)
	LEN	$27.37 ($17.78, $36.89)	$23.52 ($18.84, $28.32)	$24.66 ($21.52, $27.82)	$23.4 ($21.15, $25.68)

*Note*: MO, CAB and LEN refer to monthly oral, cabotegravir and lenacapavir, respectively. Prices are in 2021 USD. Values in parentheses show 95% uncertainty intervals representing the 2.5th and 97.5th percentiles across 100 parameter sets.

With risk‐prioritized coverage, the maximum cost per person‐year on LA‐PrEP decreased dramatically with expanding coverage (Table 3B). Cost decreases were most extreme in South Africa (e.g. $106 → $54 for 5% → 15% coverage of 6‐monthly injectable PrEP) and least extreme in Kenya (e.g. $15 → $11 for 5% → 15% coverage of 6‐monthly injectable PrEP). In non‐prioritized scenarios, maximum PrEP costs were robust to changes in coverage from 5% to 15% in Kenya and associated with small decreases in maximum costs in Zimbabwe and South Africa. Additional results including HIV acquisitions, deaths and DALYs averted and ART costs averted for each strategy are available in Appendix S2 and Tables S1−S7.

### 3.7 Additional sensitivity analyses

Assuming a longer duration of scale‐up of LA‐PrEP to reach coverage targets (10 vs. 5 years) resulted in slightly lower health benefits and cost thresholds across scenarios compared to the main analysis (Table S8). For example, in South Africa, 6‐monthly injectable PrEP with 5% risk‐prioritized coverage had a maximum cost of $99 per year with a 10‐year scale‐up versus $106 with a 5‐year scale‐up.

Scenarios of risk‐prioritized LA‐PrEP scale‐up among women only resulted in lower health benefits and cost thresholds compared to scale‐up to all adults in South Africa and Zimbabwe. However, in western Kenya, scale‐up to women was more efficient than all adults (Table S8), reflecting epidemiological differences across modelled settings. In particular, the western Kenya model accounted for a relatively high prevalence of voluntary medical male circumcision (Appendix S1 and Table S5) which drove a larger differential in HIV incidence between women versus men (much lower incidence in men) compared to other settings.

Varying LA PrEP effectiveness slightly impacted health impact and maximum cost thresholds in all three settings; however, results for South Africa were most sensitive to changes in effectiveness compared to western Kenya and Zimbabwe. For example, assuming risk‐prioritized CAB‐LA at lower bound effectiveness resulted in a maximum cost per year of $22.96 (17.94–28.18) compared to $28.30 (23.14–33.6) (base case) for western Kenya, and $10.31 (4.94–15.85) versus $11.68 (6.37–16.86) (base case) in Zimbabwe compared to $81.16 (72.34–89.81) versus $109.68 (101.16–117.99) in South Africa (Table S9). Monthly oral PrEP was more sensitive to changes in assumed effectiveness compared to CAB‐LA and LEN.

## 4 DISCUSSION

In this model‐based analysis, we evaluated the health impact and maximum cost threshold for three LA‐PrEP products in western Kenya, Zimbabwe and South Africa. Risk‐prioritized injectable LA‐PrEP could sustain higher annual costs than monthly oral PrEP and is likely to be cost‐effective at a fully loaded annual cost of $15−29 in Kenya and Zimbabwe and $106−109 in South Africa at 5% coverage. Maximum costs declined slightly with a higher coverage or more protracted LA‐PrEP scale‐up time, but remained similar to previously estimated annual generic production costs of CAB‐LA at scale, which ranged from $16 to 23 [37]. Notably, the current manufacturer of LEN has signed voluntary licenses to enable generic manufacturing. Preliminary studies estimate that high‐volume generic manufacturing could potentially reach a cost of ∼$20 per dose, although estimates require further validation [38]. Although monthly oral PrEP was projected to have a lower impact and could sustain lower annual costs, it was still projected to achieve 12–22% HIV incidence reductions and be cost‐effective at a cost per year of $11−62. Cost thresholds varied by setting, highlighting the importance of country‐specific analyses to inform product scale‐up.

We found that risk‐prioritized LA‐PrEP scale‐up can avert 4–5 times more HIV acquisitions than uniform provision to sexually active adults at the same coverage levels, with greater impact from injectable options due to lack of user dependence. However, our results rely on model assumptions of equal scale‐up, uptake and retention with all three LA‐PrEP products. Analyses should be updated as evidence accrues on product availability, delivery capacity and user preferences. For example, most trial participants have preferred CAB‐LA to daily oral PrEP, yet studies among young women in Malawi and South Africa found that some harbour a fear of needles and discomfort in having to remove their clothes for a buttocks injection [39, 40]. Upcoming implementation studies with CAB‐LA [41] will provide an important crucial evidence to narrow the wide range of model scenarios presented in this analysis.

Consistent with previous studies, we find that widespread LA‐PrEP use in the general population is unlikely to be cost‐effective unless LA‐PrEP costs are low [42, 43]. Our findings regarding 2‐monthly injectable PrEP are consistent with a previous modelling study, which projected that CAB‐LA would be cost‐effective at 2.5% population coverage at a cost of $114 per person year, similar to our maximum annual cost estimate of $113 in South Africa but higher than our estimates for Kenya and Zimbabwe. This may be driven by differences in the models used, the level of prioritization and the inclusion of other healthcare costs averted, for example HIV‐related hospitalizations [37]. Another modelling study of South Africa estimated that the threshold cost of CAB‐LA was $63−101 per year, similar to our South Africa estimate; however, authors used the threshold of the same cost‐effectiveness as daily oral PrEP, whereas we utilized $500 per DALY averted [44]. Two population‐level studies of daily oral PrEP in Africa show that making daily oral PrEP easily accessible resulted in large reductions in HIV incidence in the population despite relatively low uptake and high rates of discontinuation, suggesting that individuals were effectively using daily oral PrEP during periods of HIV risk [29, 45]. In line with these studies, it is likely that LA‐PrEP use will align with risk, but not perfectly. As such, our scenarios of risk‐prioritized versus non‐prioritized PrEP should be treated as a bounding analysis.

Our analysis has several limitations. First, we model each LA‐PrEP product separately. Adding several options of LA‐PrEP to the prevention landscape will likely alter the uptake of various products and influence cost thresholds. Second, efficacy and adherence data for monthly oral PrEP are not yet available. However, we conducted sensitivity analyses to estimate the impact of varying effectiveness on health benefits and maximum costs. Third, we compared products at the same levels of availability and timing of scale‐up to isolate the effects of the products and settings (i.e. differences across product characteristics, epidemiological settings and risk profiles of populations receiving PrEP). Delays in PrEP scale‐up resulted in lowering the maximum cost thresholds. If some PrEP products are available earlier than others, they may be able to sustain a higher maximum cost. Fourth, we did not model resistance associated with PrEP use. Studies suggest that scale‐up of CAB‐LA may result in modest increases in drug‐resistant HIV [37]. However, modelling analyses have evaluated the impact of resistance with CAB‐LA scale‐up and have found the effect on cost thresholds in South Africa to be small [37]. Fifth, due to a limitation of the modelling tool used, we did not model men who have sex with men or persons who inject drugs, which are important groups to reach with HIV prevention. Heterosexual adults bear the bulk of the HIV burden in ESA, but these findings are not likely to be generalizable to settings with concentrated HIV epidemics among key populations. Sixth, although we included ART costs avoided by effective PrEP, we did not include other costs, such as avoided costs of HIV care and hospitalizations which would likely make LA‐PrEP more cost‐effective. Finally, our analysis estimated fully loaded maximum costs, without specifying the PrEP implementation model and associated costs, for example the cost of HIV testing or self‐testing while on PrEP. Implementation costs of different PrEP modalities will likely vary. Further, costs associated with injectable PrEP delivery (estimated to be $8.55 per dose in our prior analyses) are likely higher than monthly oral PrEP which may make monthly oral more attractive [46]. Future economic evaluations should consider scale‐up and demand generation costs as well as evaluate the impact of PrEP provision on healthcare staff, infrastructure and budget allocations.

## 5 CONCLUSIONS

LA‐PrEP can have a substantial impact on the HIV epidemic if scaled up to groups who can benefit at reasonable costs. This analysis can facilitate both near‐term decision‐making regarding CAB‐LA and long‐term visions for LA‐PrEP options in ESA.

## COMPETING INTERESTS

AB declares grants from the US National Institutes of Health, The Gates Foundation, the Foundation for Innovative New Diagnostics, and the New York City Department of Health and Mental Hygiene; and personal consulting with Gates Ventures. MS reports grants from the US National Institutes of Health and The Gates Foundation.

## AUTHORS’ CONTRIBUTIONS

All authors contributed to the conceptualization of the modelling question, provided substantive input into the modelling scenarios and assumptions, interpreted results and critically reviewed manuscript drafts. DK, MM and AB contributed to the model development, coding and analysis. MS wrote the first draft of the manuscript. All authors have read and approved the final manuscript.

## FUNDING

This study was funded by the Children's Investment Foundation Fund (CIFF). DK and AB were supported by the US National Institutes of Health (NIH R01AI179417). MS, LW and CA were supported by the Gates Foundation (INV‐038274). Support for EMOD‐HIV was provided by the Gates Foundation. The content is solely the responsibility of the authors and does not necessarily represent the official views of these funders. The funders had no role in study design, data collection, analysis, writing of the report, nor the decision to submit for publication.

## Supporting information



Supporting Information

Supporting Information

## Data Availability

All data used for model calibration and scenario configuration are publicly available and can be found in summary tables in the Supplementary Appendices. EMOD‐HIV is open‐source and available online: docs.idm.org.
