# Supplementary material for: Cost thresholds for anticipated long‐acting HIV pre‐exposure prophylaxis products in Eastern and Southern Africa: a mathematical modelling study
Source: J Int AIDS Soc. 2025 Feb 24;28(2):e26427. doi: 10.1002/jia2.26427 (PMC11850439; doi:10.1002/jia2.26427)
Supplement: Supplementary file 1 — Supporting Information [file JIA2-28-e26427-s001.docx]

**Online Supplemental Appendix**

**Accompanying the manuscript:**

**Health impact and price threshold for emerging long-acting PrEP products in Eastern and Southern Africa: a mathematical modeling study**

**Contents**

[Empiric Data for Model Calibration and Validation 2](#_Toc152180395)

[HIV prevalence in counties by age and gender 2](#_Toc152180396)

[Table S1a. HIV prevalence in counties of the former Nyanza province (Homa Bay, Kisii, Kisumu, Migori, Nyamira, Siaya), by age and gender in Kenya^¥^ 2](#_Toc152180397)

[Table S1b. HIV prevalence data by age and sex from population-based surveys for model calibration in South Africa^¥^ 4](#_Toc152180398)

[Table S1c. HIV prevalence data by age and sex from population-based surveys for model calibration in Zimbabwe^¥^ 5](#_Toc152180399)

[Table S2. HIV prevalence among men and women ages 15-49, by county and gender in Kenya^¥^ 5](#_Toc152180400)

[Number of people on ART 1](#_Toc152180401)

[Table S3a. Number of people on ART by county, gender, and age group in Kenya^¥^ 1](#_Toc152180402)

[Table S3b: Number of people on ART by sex (ages 15-49 years) in South Africa ^¥^ 1](#_Toc152180403)

[Table S3c: Number of people on ART by year and sex (ages 15-49 years) in Zimbabwe ^¥^ 1](#_Toc152180404)

[Population size by age and gender 2](#_Toc152180405)

[Table S4a. Population size by gender, county, and age group in 2009 in Kenya^¥^ 2](#_Toc152180406)

[Table S4b. Population of South Africa by age and sex ^¥^ 3](#_Toc152180407)

[Table S4c. Population of Zimbabwe by age and sex in 2010^¥^ 5](#_Toc152180408)

[Voluntary medical male circumcision 1](#_Toc152180409)

[Table S5a. Circumcision status quo by county, age group, and year in Kenya ^¥^ 1](#_Toc152180410)

[Table S5b. Number of voluntary medical male circumcisions conducted in South Africa by age group^*^ 1](#_Toc152180411)

[Table S5c. Number of voluntary medical male circumcisions conducted in Zimbabwe among male aged 15-25 years old^*^ 1](#_Toc152180412)

[Age-specific population fertility rates 3](#_Toc152180413)

[Table S5a. Age-specific population fertility rates in Kenya 1950-2044^¥^ 3](#_Toc152180414)

[Table S5b. Age-specific population fertility rates in South Africa 1950-2044^¥^ 4](#_Toc152180415)

[Table S5c. Age-specific population fertility rates in Zimbabwe 1950-2044^¥^ 6](#_Toc152180416)

[Age-specific HIV-deleted Mortality Rates 6](#_Toc152180417)

[HIV-deleted mortality rates calculations 6](#_Toc152180418)

[Table S6a. Age-specific HIV deleted mortality rates in Kenya 1950-2049 by gender ^¥^ 7](#_Toc152180419)

[Table S6b. Age-specific mortality rates in South Africa 1950-2049 by gender^¥^ 8](#_Toc152180420)

[Table S6C. Age-specific HIV-deleted mortality rates in Zimbabwe 1950-2049 by gender 9](#_Toc152180421)

[Model fit to age-specific and overall prevalence from population-based surveys by sex 11](#_Toc152180422)

[Figure S1a Model fit to age-specific and overall prevalence from population-based surveys by sex in Kenya 11](#_Toc152180423)

[Figure S1b Model fit to age-specific and overall prevalence from population-based surveys by sex in South Africa 4](#_Toc152180424)

[Figure S1c. Model fit to age-specific and overall prevalence from population-based surveys by sex in Zimbabwe 9](#_Toc152180425)

[Model Overview and Parameters 12](#_Toc152180426)

[Model Parameters 14](#_Toc152180427)

[Table S7a. Select model parameters used to fit the EMOD-HIV transmission model to survey data on prevalence and ART coverage from Kenya. 14](#_Toc152180428)

[Table S7b. Select model parameters used to fit the EMOD-HIV transmission model to survey data on prevalence and ART coverage from South Africa. 16](#_Toc152180429)

[Table S7c. Select model parameters used to fit the EMOD-HIV transmission model to survey data on prevalence and ART coverage from Zimbabwe. 18](#_Toc152180430)

[Table S8. Utility weights for estimating disability-adjusted life-years averted 20](#_Toc152180431)

[References 21](#_Toc152180432)

# Empiric Data for Model Calibration and Validation

## HIV prevalence in counties by age and gender

### Table S1a. HIV prevalence in counties of the former Nyanza province (Homa Bay, Kisii, Kisumu, Migori, Nyamira, Siaya), by age and gender in Kenya^¥^

| **Age group** | **2003** | | **2007** | | **2008** | | **2012** | | **2018** | |
| --- | --- | --- | --- | --- | --- | --- | --- | --- | --- | --- |
|  | **Men** | **Women** | **Men** | **Women** | **Men** | **Women** | **Men** | **Women** | **Men** | **Women** |
| **15 - 19** | 0.0015 | 0.0459 | 0.0121 | 0.0773 | 0.0184 | 0.1078 | 0.0151 | 0.0486 | 0.0025 | 0.0294 |
| **20 - 24** | 0.0562 | 0.2997 | 0.0257 | 0.2056 | 0.0578 | 0.1201 | 0.0289 | 0.1411 | 0.0216 | 0.0910 |
| **25 - 29** | 0.2429 | 0.2301 | 0.1956 | 0.2454 | 0.2450 | 0.2228 | 0.2107 | 0.2454 | 0.0705 | 0.2334 |
| **30 - 34** | 0.1840 | 0.1632 | 0.2578 | 0.2576 | 0.1530 | 0.2593 | 0.2392 | 0.2047 | 0.0911 | 0.2696 |
| **35- 39** | 0.2064 | 0.1838 | 0.2384 | 0.2227 | 0.2275 | 0.2259 | 0.1955 | 0.2811 | 0.1675 | 0.2705 |
| **40 - 44** | 0.2533 | 0.3500 | 0.2024 | 0.1799 | 0.2501 | 0.0927 | 0.3132 | 0.1694 | 0.2075 | 0.2765 |
| **45 - 49** | 0.1624 | 0.1651 | 0.2103 | 0.1291 | 0.1331 | 0.1716 | 0.1623 | 0.2287 | 0.2796 | 0.1991 |
| **15 - 49** | 0.1160 | 0.1830 | 0.1140 | 0.1760 | 0.1140 | 0.1600 | 0.1340 | 0.1760 | 0.0826 | 0.1667 |

**^¥^Sources:** Kenya Demographic and Health Surveys, 2003 & 2008; Kenya AIDS Indicator Surveys, 2007 & 2012; Kenya Population-Based HIV Impact Assessment 2018.

### Table S1b. HIV prevalence data by age and sex from population-based surveys for model calibration in South Africa^¥^

| **Sex** | **Age group** | **Year** | | | | |
| --- | --- | --- | --- | --- | --- | --- |
|  |  | **2002** | **2005** | **2008** | **2012** | **2017** |
| **Men** | 15-19 | 0.040 | 0.032 | 0.025 | 0.007 | 0.047 |
|  | 20-24 | 0.080 | 0.060 | 0.051 | 0.051 | 0.048 |
|  | 25-29 | 0.220 | 0.121 | 0.157 | 0.173 | 0.124 |
|  | 30-34 | 0.240 | 0.233 | 0.258 | 0.256 | 0.184 |
|  | 35-39 | 0.180 | 0.233 | 0.185 | 0.288 | 0.238 |
|  | 40-44 | 0.120 | 0.175 | 0.192 | 0.158 | 0.224 |
|  | 45-49 | 0.120 | 0.103 | 0.084 | 0.134 | 0.248 |
|  | 50-54 | 0.050 | 0.142 | 0.104 | 0.155 | 0.202 |
|  | 55-59 | 0.070 | 0.064 | 0.062 | 0.055 | 0.148 |
|  | 15-49 | 0.128 | 0.117 | 0.116 | 0.145 | 0.148 |
| **Women** | 15-19 | 0.070 | 0.094 | 0.067 | 0.056 | 0.058 |
|  | 20-24 | 0.170 | 0.239 | 0.211 | 0.174 | 0.156 |
|  | 25-29 | 0.320 | 0.333 | 0.327 | 0.284 | 0.275 |
|  | 30-34 | 0.240 | 0.260 | 0.291 | 0.360 | 0.347 |
|  | 35-39 | 0.140 | 0.193 | 0.248 | 0.316 | 0.394 |
|  | 40-44 | 0.190 | 0.124 | 0.163 | 0.280 | 0.359 |
|  | 45-49 | 0.110 | 0.087 | 0.141 | 0.197 | 0.303 |
|  | 50-54 | 0.080 | 0.075 | 0.102 | 0.148 | 0.222 |
|  | 55-59 | 0.070 | 0.030 | 0.077 | 0.097 | 0.176 |
|  | 15-49 | 0.177 | 0.202 | 0.213 | 0.232 | 0.263 |

^¥^Sources: South African National HIV Prevalence, Incidence and Behaviour Surveys (2002, 2005, 2008, 2012 and 2017) from the Human Sciences Research Council (HSRC)

### Table S1c. HIV prevalence data by age and sex from population-based surveys for model calibration in Zimbabwe^¥^

| **Age group** | **2006** | | **2011** | | **2015** | | **2016** | |
| --- | --- | --- | --- | --- | --- | --- | --- | --- |
|  | **Men** | **Women** | **Men** | **Women** | **Men** | **Women** | **Men** | **Women** |
| **15 - 19** | 0.031 | 0.062 | 0.034 | 0.042 | 0.025 | 0.040 | 0.032 | 0.039 |
| **20 - 24** | 0.058 | 0.163 | 0.038 | 0.106 | 0.037 | 0.103 | 0.027 | 0.081 |
| **25 - 29** | 0.130 | 0.288 | 0.103 | 0.201 | 0.075 | 0.155 | 0.066 | 0.143 |
| **30 - 34** | 0.295 | 0.355 | 0.174 | 0.290 | 0.131 | 0.219 | 0.122 | 0.219 |
| **35- 39** | 0.321 | 0.345 | 0.251 | 0.291 | 0.180 | 0.280 | 0.194 | 0.266 |
| **40 - 44** | 0.329 | 0.257 | 0.262 | 0.257 | 0.270 | 0.313 | 0.254 | 0.296 |
| **45 - 49** | 0.260 | 0.180 | 0.299 | 0.225 | 0.232 | 0.243 | 0.281 | 0.289 |
| **15 - 49** | 0.145 | 0.211 | 0.123 | 0.177 | 0.105 | 0.167 | - | - |

Zimbabwe Demographic and Health Surveys 2005-06, 2010-22, 2015. Zimbabwe Population-Based HIV Impact Assessment 2016.

### Table S2. HIV prevalence among men and women ages 15-49, by county and gender in Kenya^¥^

| **County** | **2003** | | **2007** | | **2008** | | **2012** | | **2018** | |
| --- | --- | --- | --- | --- | --- | --- | --- | --- | --- | --- |
|  | **Men** | **Women** | **Men** | **Women** | **Men** | **Women** | **Men** | **Women** | **Men** | **Women** |
| **Homa Bay** | 0.1097 | 0.2458 | 0.2514 | 0.3259 | 0.1737 | 0.2524 | 0.2217 | 0.2787 | 0.1279 | 0.2532 |
| **Kisii** | 0.0114 | 0.0853 | 0.0445 | 0.0693 | 0.0330 | 0.0573 | 0.0346 | 0.0368 | 0.0458 | 0.0684 |
| **Kisumu** | 0.1663 | 0.1914 | 0.1139 | 0.1847 | 0.1109 | 0.1810 | 0.1940 | 0.2022 | 0.0960 | 0.2096 |
| **Migori** | 0.1804 | 0.1860 | 0.1685 | 0.2181 | 0.1923 | 0.2228 | 0.1435 | 0.1925 | 0.0706 | 0.1758 |
| **Nyamira** | 0.0029 | 0.0742 | - | - | 0.0234 | 0.0544 | 0.0419 | 0.1045 | 0.0247 | 0.0432 |
| **Siaya** | 0.1824 | 0.2424 | 0.1445 | 0.2130 | 0.1526 | 0.1921 | 0.2596 | 0.2990 | 0.0961 | 0.1905 |

**^¥^Sources:** Kenya Demographic and Health Surveys, 2003 & 2008; Kenya AIDS Indicator Surveys, 2007 & 2012; Kenya Population-Based HIV Impact Assessment 2018.

## Number of people on ART

### Table S3a. Number of people on ART by county, gender, and age group in Kenya^¥^

| **Gender** | **County** | **Age group** | | **Year** | | | | | | | | | | | | | | |
| --- | --- | --- | --- | --- | --- | --- | --- | --- | --- | --- | --- | --- | --- | --- | --- | --- | --- | --- |
|  |  |  |  | **2004** | **2005** | **2006** | **2007** | **2008** | **2009** | **2010** | **2011** | **2012** | **2013** | **2014** | **2015** | **2016** | **2017** |  |
| **Men** | **Homa Bay** | 0 - 14 | | - | - | - | - | - | - | - | - | - | - | 2,945 | 3,583 | 4,109 | 4,192 |  |
|  |  | 15 - 99 | | 1,067 | 2,313 | 5,148 | 7,194 | 10,002 | 14,436 | 17,178 | 15,954 | 17,522 | 18,279 | 19,157 | 22,834 | 26,441 | 29,220 |  |
|  | **Kisii** | 0 - 14 | | - | - | - | - | - | - | - | - | - | - | 828 | 993 | 1,109 | 1,083 |  |
|  |  | 15 - 99 | | - | - | - | - | - | - | - | 2,972 | - | - | 4,614 | 5,451 | 6,604 | 7,169 |  |
|  | **Kisumu** | 0 - 14 | | - | - | - | - | - | - | - | - | - | - | 3,101 | 3,245 | 3,525 | 3,607 |  |
|  |  | 15 - 99 | | 945 | 2,047 | 4,557 | 6,368 | 8,853 | 12,779 | 15,206 | 14,122 | 15,511 | 16,180 | 21,216 | 24,550 | 28,082 | 31,021 |  |
|  | **Migori** | 0 - 14 | |  |  |  |  |  |  |  |  |  |  | 2,309 | 2,295 | 2,678 | 2,673 |  |
|  |  | 15 - 99 | | 711 | 1,541 | 3,430 | 4,793 | 6,664 | 9,619 | 11,446 | 10,630 | 11,675 | 12,179 | 13,929 | 15,165 | 17,438 | 18,455 |  |
|  | **Nyamira** | 0 - 14 | | - | - | - | - | - | - | - | - | - | - | 484 | 552 | 578 | 611 |  |
|  |  | 15 - 99 | | - | - | - | - | - | - | - | 1,362 | - | - | 2,120 | 2,585 | 3,142 | 3,474 |  |
|  | **Siaya** | 0 - 14 | | - | - | - | - | - | - | - | - | - | - | 2,645 | 2,950 | 3,017 | 3,197 |  |
|  |  | 15 - 99 | | 860 | 1,864 | 4,148 | 5,797 | 8,060 | 11,633 | 13,843 | 12,856 | 14,120 | 14,730 | 16,163 | 18,611 | 21,477 | 23,762 |  |
| **Women** | **Homa Bay** | 0 - 14 | | - | - | - | - | - | - | - | - | - | - | 3,431 | 3,835 | 4,426 | 4,535 |  |
|  |  | 15 - 99 | | 1,359 | 2,944 | 6,551 | 9,155 | 12,522 | 17,202 | 21,798 | 31,454 | 35,182 | 38,819 | 40,118 | 49,956 | 57,286 | 61,811 |  |
|  | **Kisii** | 0 - 14 | | - | - | - | - | - | - | - | - | - | - | 906 | 1,079 | 1,200 | 1,146 |  |
|  |  | 15 - 99 | | - | - | - | - | - | - | - | 7,902 | - | - | 11,691 | 14,350 | 17,274 | 19,044 |  |
|  | **Kisumu** | 0 - 14 | | - | - | - | - | - | - | - | - | - | - | 3,241 | 3,393 | 3,810 | 3,831 |  |
|  |  | 15 - 99 | | 1,203 | 2,606 | 5,799 | 8,104 | 11,084 | 15,227 | 19,296 | 27,843 | 31,143 | 34,362 | 41,230 | 48,424 | 56,384 | 60,789 |  |
|  | **Migori** | 0 - 14 | | - | - | - | - | - | - | - | - | - | - | 2,526 | 2,448 | 2,868 | 2,884 |  |
|  |  | 15 - 99 | | 905 | 1,961 | 4,365 | 6,100 | 8,343 | 11,461 | 14,524 | 20,958 | 23,442 | 25,865 | 27,896 | 31,964 | 38,637 | 40,891 |  |
|  | **Nyamira** | 0 - 14 | | - | - | - | - | - | - | - | - | - | - | 506 | 567 | 601 | 622 |  |
|  |  | 15 - 99 | | - | - | - | - | - | - | - | 3,766 | - | - | 5,964 | 7,210 | 8,258 | 8,654 |  |
|  | **Siaya** | 0 - 14 | | - | - | - | - | - | - | - | - | - | - | 2,778 | 3,136 | 3,299 | 3,569 |  |
|  |  | 15 - 99 | | 1,095 | 2,372 | 5,279 | 7,377 | 10,090 | 13,862 | 17,566 | 25,347 | 28,351 | 31,281 | 33,911 | 39,853 | 44,892 | 48,808 |  |
| **Both** | **All** | | 15-99 | 8,954 | 19,404 | 43,185 | 60,350 | 83,142 | 116,787 | 143,877 | 175,003 | 194,552 | 210,769 | 246,293 | 301,029 | 333,333 | 389,159 |  |

**^¥^**Source: Kenya Ministry of Health

### Table S3b: Number of people on ART by sex (ages 15-49 years) in South Africa ^¥^

| **Year** | **Male** | **Female** |
| --- | --- | --- |
| 2001 | 2713 | 3543 |
| 2002 | 5768 | 7586 |
| 2003 | 9321 | 12313 |
| 2004 | 17717 | 24423 |
| 2005 | 34874 | 59240 |
| 2006 | 66629 | 123308 |
| 2007 | 118977 | 228753 |
| 2008 | 186564 | 367389 |
| 2009 | 277931 | 548206 |
| 2010 | 402000 | 781477 |
| 2011 | 558131 | 1095411 |
| 2012 | 713294 | 1415016 |
| 2013 | 876749 | 1732515 |
| 2014 | 1013499 | 2003014 |
| 2015 | 1130063 | 2239669 |
| 2016 | 1238815 | 2518238 |
| 2017 | 1403702 | 2998170 |

^¥^Source: South Africa Department of Health Surveys

### Table S3c: Number of people on ART by year and sex (ages 15-49 years) in Zimbabwe ^¥^

| Year | Male | Female |
| --- | --- | --- |
| 2004 | 4854 | 6146 |
| 2005 | 10049 | 15818 |
| 2006 | 22323 | 31309 |
| 2007 | 37527 | 52562 |
| 2008 | 57603 | 77120 |
| 2009 | 82266 | 117364 |
| 2010 | 119235 | 211144 |
| 2011 | 148551 | 296112 |
| 2012 | 178027 | 340774 |
| 2013 | 245690 | 373190 |
| 2014 | 264614 | 468305 |
| 2016 | 361399 | 579378 |
| 2017 | 388658 | 663132 |

^¥^Zimbabwe Ministry of Health

## Population size by age and gender

### Table S4a. Population size by gender, county, and age group in 2009 in Kenya^¥^

| **Age Group** | **Men** | | | | | | **Women** | | | | | |
| --- | --- | --- | --- | --- | --- | --- | --- | --- | --- | --- | --- | --- |
|  | **Homa Bay** | **Kisii** | **Kisumu** | **Migori** | **Nyamira** | **Siaya** | **Homa Bay** | **Kisii** | **Kisumu** | **Migori** | **Nyamira** | **Siaya** |
| **0 - < 1** | 18,335 | 18,236 | 17,457 | 19,265 | 10,313 | 15,093 | 18,354 | 17,993 | 16,926 | 19,309 | 10,263 | 14,860 |
| **1 - 4** | 69,799 | 69,529 | 63,054 | 69,921 | 41,165 | 56,269 | 69,250 | 69,023 | 63,172 | 69,519 | 40,396 | 55,901 |
| **5 - 9** | 75,926 | 76,757 | 67,083 | 73,872 | 46,450 | 60,966 | 75,973 | 75,778 | 67,779 | 74,333 | 46,867 | 60,710 |
| **10 - 14** | 68,689 | 68,473 | 62,706 | 64,300 | 42,590 | 58,296 | 67,159 | 68,072 | 63,359 | 63,249 | 42,198 | 56,248 |
| **15 - 19** | 57,430 | 59,228 | 55,597 | 53,075 | 36,604 | 49,220 | 54,119 | 60,776 | 56,741 | 52,238 | 36,786 | 47,825 |
| **20 - 24** | 39,573 | 41,898 | 47,281 | 38,690 | 24,409 | 32,725 | 50,309 | 58,225 | 57,649 | 48,004 | 34,184 | 41,443 |
| **25 - 29** | 30,437 | 32,792 | 40,964 | 30,727 | 19,515 | 25,961 | 36,016 | 42,878 | 40,614 | 34,670 | 27,273 | 30,135 |
| **30 - 34** | 23,259 | 26,678 | 30,412 | 23,344 | 16,605 | 20,359 | 26,342 | 30,031 | 27,515 | 25,630 | 19,487 | 22,328 |
| **34 - 39** | 16,013 | 21,766 | 21,251 | 17,024 | 14,039 | 14,793 | 20,010 | 26,051 | 20,611 | 19,313 | 17,106 | 17,932 |
| **40 - 44** | 11,914 | 15,718 | 15,145 | 12,170 | 10,470 | 11,118 | 16,513 | 18,360 | 16,894 | 14,773 | 11,377 | 16,082 |
| **45 - 49** | 11,124 | 16,797 | 13,361 | 10,549 | 11,318 | 10,390 | 15,248 | 19,181 | 15,298 | 12,888 | 11,886 | 15,486 |
| **50 - 54** | 9,705 | 12,789 | 11,251 | 8,565 | 8,379 | 9,079 | 12,942 | 14,136 | 12,504 | 10,314 | 8,703 | 14,541 |
| **55 - 59** | 8,159 | 9,527 | 8,718 | 6,399 | 5,999 | 8,414 | 9,833 | 9,528 | 9,175 | 7,692 | 5,819 | 12,265 |
| **60 - 64** | 6,989 | 7,395 | 7,054 | 5,250 | 5,026 | 7,712 | 8,587 | 7,654 | 7,597 | 6,000 | 5,107 | 11,081 |
| **65 - 69** | 4,325 | 4,637 | 4,163 | 3,382 | 3,094 | 5,107 | 5,957 | 5,320 | 5,402 | 4,508 | 3,322 | 7,732 |
| **70 - 74** | 4,029 | 3,945 | 3,777 | 2,907 | 2,753 | 5,175 | 5,355 | 5,017 | 4,757 | 3,524 | 3,153 | 7,173 |
| **75 - 79** | 2,835 | 2,743 | 2,392 | 2,033 | 1,778 | 3,549 | 3,891 | 3,338 | 3,356 | 2,968 | 1,919 | 5,464 |
| **80 - 99** | 3,726 | 3,701 | 2,821 | 2,624 | 2,393 | 4,159 | 5,316 | 5,891 | 4,615 | 3,636 | 3,475 | 6,155 |

**^¥^**Source: Kenya National Bureau of Statistics, 2009 Census

### Table S4b. Population of South Africa by age and sex ^¥^

| **Sex** | **Age group** | **Year** | | | | |
| --- | --- | --- | --- | --- | --- | --- |
|  |  | **2002** | **2005** | **2008** | **2012** | **2017** |
| **Men** | 0 - 4 | 2634839 | 2651819 | 2692300 | 2784372 | 2886299 |
|  | 5 - 9 | 2559246 | 2570683 | 2592577 | 2644720 | 2767111 |
|  | 10 - 14 | 2582620 | 2565499 | 2557928 | 2578751 | 2636981 |
|  | 15 - 19 | 2546968 | 2596517 | 2584945 | 2572032 | 2587023 |
|  | 20 - 24 | 2348165 | 2488613 | 2572307 | 2606447 | 2594656 |
|  | 25 - 29 | 2069939 | 2215800 | 2362377 | 2530034 | 2612453 |
|  | 30 - 34 | 1759391 | 1899279 | 2018461 | 2227862 | 2488823 |
|  | 35 - 39 | 1488894 | 1568198 | 1664587 | 1845019 | 2131687 |
|  | 40 - 44 | 1270053 | 1324166 | 1367150 | 1493652 | 1731088 |
|  | 45 - 49 | 1077752 | 1122009 | 1159644 | 1234887 | 1398602 |
|  | 50 - 54 | 831061 | 925655 | 973184 | 1038560 | 1147992 |
|  | 55 - 59 | 636945 | 677128 | 760379 | 856728 | 947123 |
| **Women** | 0 - 4 | 2587465 | 2602664 | 2640997 | 2729586 | 2825703 |
|  | 5 - 9 | 2523770 | 2535299 | 2555462 | 2604517 | 2719700 |
|  | 10 - 14 | 2557891 | 2533039 | 2526253 | 2545219 | 2603685 |
|  | 15 - 19 | 2527828 | 2568409 | 2552976 | 2535920 | 2555420 |
|  | 20 - 24 | 2323512 | 2451570 | 2523985 | 2560705 | 2550570 |
|  | 25 - 29 | 2064262 | 2167428 | 2282514 | 2447992 | 2550351 |
|  | 30 - 34 | 1807453 | 1906808 | 1967958 | 2123170 | 2394266 |
|  | 35 - 39 | 1577255 | 1648612 | 1717873 | 1821711 | 2039776 |
|  | 40 - 44 | 1370835 | 1434647 | 1491937 | 1594914 | 1745050 |
|  | 45 - 49 | 1184477 | 1243314 | 1302936 | 1396666 | 1541175 |
|  | 50 - 54 | 924873 | 1054059 | 1124243 | 1214556 | 1348848 |
|  | 55 - 59 | 727478 | 783876 | 898804 | 1035986 | 1160340 |

^¥^Source: United Nations Population Database

### Table S4c. Population of Zimbabwe by age and sex in 2010^¥^

| Age Group (years) | Male | Female |
| --- | --- | --- |
| 0-4 | 1003778 | 993503 |
| 5-9 | 849773 | 849884 |
| 10-14 | 791114 | 795446 |
| 15-19 | 729897 | 766954 |
| 20-24 | 646316 | 730145 |
| 25-29 | 511221 | 596614 |
| 30-34 | 407730 | 471291 |
| 35-39 | 296577 | 333524 |
| 40-44 | 220200 | 245985 |
| 45-49 | 163800 | 200252 |
| 50-54 | 125947 | 167229 |
| 55-59 | 99658 | 142147 |
| 60-64 | 68299 | 100376 |
| 65-69 | 60276 | 94053 |
| 70-74 | 39694 | 70357 |
| 75-79 | 21954 | 48407 |
| 80-84 | 9792 | 28377 |
| 85-89 | 2574 | 10891 |
| 90-94 | -- | 2858 |

Source: Zimbabwe National Statistics Agency.

## Voluntary medical male circumcision

### Table S5a. Circumcision status quo by county, age group, and year in Kenya ^¥^

|  | **Homa Bay** | | | **Kisii** | | | **Kisumu** | | | | **Migori** | | | | **Nyamira** | | | | **Siaya** | | | |
| --- | --- | --- | --- | --- | --- | --- | --- | --- | --- | --- | --- | --- | --- | --- | --- | --- | --- | --- | --- | --- | --- | --- |
| **Year** | **10-14** | **15-24** | **25-49** | **10-14** | **15-24** | **25-49** | **10-14** | **15-24** | **25-49** | **10-14** | | **15-24** | **25-49** | **10-14** | | **15-24** | **25-49** | **10-14** | | **15-24** | **25-49** |  |
| **Pre-2008** | 0.249 | 0.249 | 0.249 | 0.948 | 0.948 | 0.948 | 0.322 | 0.322 | 0.322 | 0.410 | | 0.410 | 0.410 | 0.965 | | 0.965 | 0.965 | 0.252 | | 0.252 | 0.252 |  |
| **2008** | 0.118 | 0.235 | 0.275 | 0.948 | 0.948 | 0.948 | 0.152 | 0.303 | 0.333 | 0.194 | | 0.385 | 0.451 | 0.965 | | 0.965 | 0.965 | 0.119 | | 0.243 | 0.270 |  |
| **2009** | 0.122 | 0.240 | 0.283 | 0.948 | 0.948 | 0.948 | 0.178 | 0.315 | 0.339 | 0.199 | | 0.388 | 0.463 | 0.965 | | 0.965 | 0.965 | 0.127 | | 0.250 | 0.277 |  |
| **2010** | 0.166 | 0.285 | 0.299 | 0.948 | 0.948 | 0.948 | 0.324 | 0.389 | 0.362 | 0.214 | | 0.395 | 0.478 | 0.965 | | 0.965 | 0.965 | 0.222 | | 0.310 | 0.293 |  |
| **2011** | 0.226 | 0.355 | 0.313 | 0.948 | 0.948 | 0.948 | 0.450 | 0.479 | 0.385 | 0.280 | | 0.423 | 0.486 | 0.965 | | 0.965 | 0.965 | 0.293 | | 0.375 | 0.304 |  |
| **2012** | 0.337 | 0.486 | 0.339 | 0.948 | 0.948 | 0.948 | 0.516 | 0.559 | 0.409 | 0.516 | | 0.530 | 0.509 | 0.965 | | 0.965 | 0.965 | 0.417 | | 0.483 | 0.324 |  |
| **2013** | 0.368 | 0.565 | 0.361 | 0.948 | 0.948 | 0.948 | 0.564 | 0.634 | 0.436 | 0.587 | | 0.614 | 0.528 | 0.965 | | 0.965 | 0.965 | 0.435 | | 0.549 | 0.340 |  |
| **2014** | 0.471 | 0.710 | 0.399 | 0.948 | 0.948 | 0.948 | 0.620 | 0.712 | 0.467 | 0.717 | | 0.726 | 0.554 | 0.965 | | 0.965 | 0.965 | 0.537 | | 0.659 | 0.368 |  |
| **2015** | 0.506 | 0.811 | 0.438 | 0.948 | 0.948 | 0.948 | 0.672 | 0.790 | 0.502 | 0.742 | | 0.813 | 0.580 | 0.965 | | 0.965 | 0.965 | 0.562 | | 0.739 | 0.395 |  |
| **2016** | 0.476 | 0.894 | 0.488 | 0.948 | 0.948 | 0.948 | 0.756 | 0.844 | 0.538 | 0.697 | | 0.891 | 0.613 | 0.965 | | 0.965 | 0.965 | 0.628 | | 0.841 | 0.425 |  |
| **2017** | 0.489 | 0.937 | 0.537 | 0.948 | 0.948 | 0.948 | 0.863 | 0.889 | 0.572 | 0.680 | | 0.949 | 0.648 | 0.965 | | 0.965 | 0.965 | 0.759 | | 0.917 | 0.457 |  |
| **2018** | 0.589 | 0.925 | 0.637 | 0.948 | 0.948 | 0.948 | 0.944 | 0.925 | 0.651 | 0.775 | | 0.976 | 0.744 | 0.965 | | 0.965 | 0.965 | 0.844 | | 0.959 | 0.562 |  |
| **2019** | 0.741 | 0.904 | 0.678 |  |  |  | 0.784 | 0.943 | 0.677 | 0.785 | |  | 0.775 |  | |  |  | 0.790 | | 0.976 | 0.596 |  |
| **2020** | 0.802 | 0.901 | 0.717 |  |  |  | 0.787 | 0.925 | 0.703 | 0.800 | |  | 0.808 |  | |  |  | 0.800 | |  | 0.633 |  |
| **2021** | 0.802 | 0.909 | 0.749 |  |  |  | 0.836 | 0.913 | 0.727 |  | |  | 0.836 |  | |  |  |  | |  | 0.668 |  |
| **2022** |  | 0.917 | 0.779 |  |  |  | 0.804 | 0.947 | 0.750 |  | |  | 0.865 |  | |  |  |  | |  | 0.704 |  |
| **2023** |  | 0.926 | 0.807 |  |  |  | 0.804 |  | 0.773 |  | |  | 0.892 |  | |  |  |  | |  | 0.738 |  |
| **2024** |  | 0.935 | 0.833 |  |  |  | 0.804 |  | 0.795 |  | |  | 0.918 |  | |  |  |  | |  | 0.772 |  |
| **2025** |  | 0.944 | 0.858 |  |  |  | 0.804 |  | 0.815 |  | |  | 0.942 |  | |  |  |  | |  | 0.803 |  |
| **2026** |  | 0.949 | 0.879 |  |  |  | 0.801 |  | 0.832 |  | |  | 0.961 |  | |  |  |  | |  | 0.832 |  |
| **2027** |  | 0.953 | 0.898 |  |  |  | 0.801 |  | 0.848 |  | |  | 0.976 |  | |  |  |  | |  | 0.976 |  |
| **2028** |  | 0.955 | 0.915 |  |  |  | 0.801 |  | 0.862 |  | |  |  |  | |  |  |  | |  |  |  |
| **2029** |  |  | 0.931 |  |  |  | 0.801 |  | 0.874 |  | |  |  |  | |  |  |  | |  |  |  |
| **2030** |  |  | 0.945 |  |  |  | 0.801 |  | 0.885 |  | |  |  |  | |  |  |  | |  |  |  |
| **2031** |  |  | 0.955 |  |  |  | 0.802 |  | 0.897 |  | |  |  |  | |  |  |  | |  |  |  |
| **2032** |  |  |  |  |  |  |  |  | 0.907 |  | |  |  |  | |  |  |  | |  |  |  |
| **2033** |  |  |  |  |  |  |  |  | 0.917 |  | |  |  |  | |  |  |  | |  |  |  |
| **2034** |  |  |  |  |  |  |  |  | 0.924 |  | |  |  |  | |  |  |  | |  |  |  |
| **2035** |  |  |  |  |  |  |  |  | 0.931 |  | |  |  |  | |  |  |  | |  |  |  |

**^¥^Source:** Circumcision prevalence prior to 2008 is obtained from the Kenya Demographic and Health Survey, 2003. Prevalence of circumcision from 2008 onward combines prevalence of traditional male circumcision and voluntary medical male circumcision estimates obtained from the Decision-Makers' Program Planning Toolkit 2.

### Table S5b. Number of voluntary medical male circumcisions conducted in South Africa by age group^*^

| **Year** | **Age Group** | | | | | |
| --- | --- | --- | --- | --- | --- | --- |
|  | **10 - 14** | **15 - 19** | **20 - 24** | **25 - 34** | **35 - 49** | **≥50** |
| **2010** | 55,431 | 30,552 | 15,856 | 18,047 | 7,864 | 1,160 |
| **2011** | 137,648 | 75,866 | 39,374 | 44,816 | 19,527 | 2,881 |
| **2012** | 175,060 | 96,487 | 50,075 | 56,996 | 24,834 | 3,664 |
| **2013** | 156,496 | 86,255 | 44,765 | 50,952 | 22,201 | 3,276 |
| **2014** | 199,750 | 110,095 | 57,138 | 65,035 | 28,337 | 4,181 |
| **2015** | 199,535 | 109,976 | 57,076 | 64,965 | 28,306 | 4,176 |
| **2016** | 165,672 | 91,312 | 47,390 | 53,940 | 23,502 | 3,468 |
| **2017** | 203,960 | 112,415 | 58,342 | 66,406 | 28,934 | 4,269 |
| **2018** | 279,500 | 154,050 | 79,950 | 91,000 | 39,650 | 5,850 |
| **2019** | 258,000 | 142,200 | 73,800 | 84,000 | 36,600 | 5,400 |
| **2020** | 236,500 | 130,350 | 67,650 | 77,000 | 33,550 | 4,950 |
| **2021** | 107,500 | 59,250 | 30,750 | 35,000 | 15,250 | 2,250 |
| **2022 onwards** | 43,000 | 23,700 | 12,300 | 14,000 | 6,100 | 900 |

^*^Source: South Africa Department of Health (unpublished data) and South Africa National Strategic Plan for HIV, TB, and STIs 2017-2022(25).

### Table S5c. Number of voluntary medical male circumcisions conducted in Zimbabwe among male aged 15-25 years old^*^

| **Year** | **Target # of  males**  **(15-25 years old)** |
| --- | --- |
| **2008.5** | 2784 |
| **2009.5** | 9381 |
| **2010.5** | 27973 |
| **2011.5** | 29321 |
| **2012.5** | 65679 |
| **2013.5** | 110163 |
| **2014.5** | 133012 |
| **2015.5** | 136986 |
| **2016.5** | 159476 |
| **2017.5** | 197705 |
| **2018.5** | 207375 |
| **2019.5** | 53743 |
| **2020.5** | 53743 |
| **2021.5-2040.5** | 4288000 |

Source: McGillen JB, Stover J, Klein DJ, Xaba Sinokuthemba, et al. The emerging health impact of voluntary medical male circumcision in Zimbabwe: An evaluation using three epidemiological models. Jul 2018 PLOS One. https://doi.org/10.1371/journal.pone.0199453

## Age-specific population fertility rates

### Table S5a. Age-specific population fertility rates in Kenya 1950-2044^¥^

|  | **Age-specific fertility rates (births per 1,000 women)** | | | | | | |
| --- | --- | --- | --- | --- | --- | --- | --- |
| **Year** | **15-19** | **20-24** | **25-29** | **30-34** | **35-39** | **40-44** | **45-49** |
| **1950-1955** | 169.1 | 351.6 | 338.1 | 284.3 | 203.5 | 110.7 | 38.9 |
| **1955-1960** | 175.9 | 365.9 | 351.9 | 295.8 | 211.8 | 115.2 | 40.5 |
| **1960-1965** | 182.3 | 379.1 | 364.5 | 306.5 | 219.4 | 119.4 | 41.9 |
| **1965-1970** | 183.3 | 381.2 | 366.6 | 308.2 | 220.6 | 120 | 42.2 |
| **1970-1975** | 180.6 | 375.5 | 361.1 | 303.6 | 217.3 | 118.3 | 41.5 |
| **1975-1980** | 172.7 | 359.1 | 345.3 | 290.3 | 207.8 | 113.1 | 39.7 |
| **1980-1985** | 163.1 | 339.2 | 326.2 | 274.2 | 196.3 | 106.8 | 37.5 |
| **1985-1990** | 147.8 | 307.3 | 295.5 | 248.4 | 177.8 | 96.8 | 34.0 |
| **1990-1995** | 115.3 | 268.9 | 252.0 | 206.8 | 161.6 | 73.4 | 52.0 |
| **1995-2000** | 111.5 | 260.7 | 253.3 | 196.2 | 143.3 | 62.4 | 42.7 |
| **2000-2005** | 104.2 | 243.6 | 236.7 | 183.4 | 133.9 | 58.3 | 39.9 |
| **2005-2010** | 97.1 | 227.1 | 221.4 | 170.6 | 123.7 | 53.6 | 36.5 |
| **2010-2015** | 86.2 | 201.9 | 202.3 | 149.2 | 102.1 | 42.4 | 27.9 |
| **2015-2020** | 75.1 | 176.5 | 179.8 | 129.6 | 85.8 | 34.8 | 22.4 |
| **2020-2024** | 69.9 | 165.1 | 171.8 | 120.2 | 76.2 | 29.9 | 18.6 |
| **2025-2029** | 65.0 | 154.7 | 164.8 | 112.6 | 68.5 | 26.0 | 15.5 |
| **2030-2034** | 60.6 | 145.7 | 159.1 | 106.7 | 62.5 | 22.9 | 13.1 |
| **2035-2039** | 56.2 | 137.2 | 153.6 | 101.8 | 57.6 | 20.4 | 11.0 |
| **2040-2044** | 52.2 | 129.7 | 149.3 | 98.2 | 53.8 | 18.5 | 9.4 |

**^¥^Source**: 2019 World Population Prospects

### Table S5b. Age-specific population fertility rates in South Africa 1950-2044^¥^

|  | **Age-specific fertility rates (births per 1,000 women)** | | | | | | |
| --- | --- | --- | --- | --- | --- | --- | --- |
| **Year** | **15-19** | **20-24** | **25-29** | **30-34** | **35-39** | **40-44** | **45-49** |
| **1950-1955** | 66.8 | 265 | 291.9 | 242.2 | 189.8 | 132 | 72.3 |
| **1955-1960** | 65.7 | 260.8 | 287.3 | 238.3 | 186.7 | 130 | 71.2 |
| **1960-1965** | 64.7 | 256.6 | 282.7 | 234.5 | 183.7 | 127.9 | 70 |
| **1965-1970** | 60.4 | 239.7 | 264.1 | 219.1 | 171.7 | 119.5 | 65.4 |
| **1970-1975** | 76.1 | 233.9 | 253.6 | 211 | 160.2 | 105.1 | 54 |
| **1975-1980** | 86.1 | 217.3 | 231.8 | 193.6 | 142.3 | 87.4 | 41.5 |
| **1980-1985** | 93.6 | 201.1 | 211.3 | 177 | 125.9 | 71.7 | 30.5 |
| **1985-1990** | 95.4 | 179.4 | 185.7 | 155.9 | 107.3 | 56 | 20.4 |
| **1990-1995** | 90.8 | 152.2 | 155.2 | 130.8 | 86.9 | 41 | 11.7 |
| **1995-2000** | 80.6 | 140.5 | 142.5 | 111.5 | 74.4 | 31 | 10.3 |
| **2000-2005** | 70.7 | 139 | 141.8 | 105.6 | 67.4 | 27.1 | 8.8 |
| **2005-2010** | 59.2 | 131.7 | 135.1 | 95.9 | 58.4 | 22.6 | 7.1 |
| **2010-2015** | 50.9 | 129 | 133.1 | 90.2 | 52.3 | 19.4 | 5.9 |
| **2015-2020** | 43.6 | 127 | 131.6 | 85.3 | 47 | 16.6 | 4.8 |
| **2020-2024** | 37.2 | 125.7 | 130.8 | 81.3 | 42.4 | 14.1 | 3.8 |
| **2025-2029** | 31.4 | 124.8 | 130.4 | 77.8 | 38.3 | 11.9 | 2.9 |
| **2030-2034** | 26.2 | 124.5 | 130.4 | 74.8 | 34.6 | 9.8 | 2.1 |
| **2035-2039** | 21.5 | 124.8 | 131.3 | 72.5 | 31.4 | 7.9 | 1.4 |
| **2040-2044** | 17 | 125.7 | 132.6 | 70.6 | 28.5 | 6.2 | 0.6 |

**^¥^Source**: 2012 World Population Prospects

### Table S5c. Age-specific population fertility rates in Zimbabwe 1950-2044^¥^

|  | **Age-specific fertility rates (births per 1,000 women)** | | | | | | |
| --- | --- | --- | --- | --- | --- | --- | --- |
| **Year** | **15-19** | **20-24** | **25-29** | **30-34** | **35-39** | **40-44** | **45-49** |
| **1950-1955** | 159.9 | 296.1 | 289.3 | 261.5 | 188.6 | 124.2 | 40.4 |
| **1955-1960** | 164.6 | 304.8 | 297.8 | 269.2 | 194.2 | 127.8 | 41.6 |
| **1960-1965** | 171.7 | 317.8 | 310.5 | 280.8 | 202.5 | 133.3 | 43.4 |
| **1965-1970** | 174.0 | 322.2 | 314.8 | 284.6 | 205.3 | 135.1 | 44.0 |
| **1970-1975** | 174.0 | 322.2 | 314.8 | 284.6 | 205.3 | 135.1 | 44.0 |
| **1975-1980** | 171.7 | 317.8 | 310.5 | 280.8 | 202.5 | 133.3 | 43.4 |
| **1980-1985** | 127.9 | 280.5 | 280.7 | 249.2 | 188.7 | 98.5 | 34.9 |
| **1985-1990** | 110.6 | 241.6 | 240.0 | 214.4 | 161.6 | 81.1 | 25.2 |
| **1990-1995** | 102.7 | 207.0 | 195.0 | 171.9 | 126.7 | 60.5 | 19.0 |
| **1995-2000** | 100.1 | 190.7 | 178.9 | 143.9 | 103.2 | 46.1 | 14.1 |
| **2000-2005** | 100.4 | 190.2 | 171.8 | 136.8 | 92.8 | 40.8 | 11.3 |
| **2005-2010** | 111.1 | 202.9 | 178.2 | 142.7 | 94.8 | 38.3 | 9.1 |
| **2010-2015** | 108.8 | 209.3 | 197.8 | 153.3 | 104.0 | 37.5 | 7.1 |
| **2015-2020** | 86.1 | 184.4 | 174.0 | 149.1 | 90.3 | 35.9 | 5.3 |
| **2020-2024** | 71.7 | 166.2 | 162.5 | 143.9 | 83.5 | 32.8 | 3.9 |
| **2025-2029** | 60.2 | 150.7 | 152.8 | 139.5 | 77.8 | 30.3 | 2.9 |
| **2030-2034** | 51.0 | 137.6 | 144.7 | 135.9 | 73.2 | 28.1 | 2.2 |
| **2035-2039** | 43.6 | 126.2 | 137.7 | 132.9 | 69.4 | 26.2 | 1.7 |
| **2040-2044** | 37.4 | 115.9 | 131.3 | 129.9 | 66.0 | 24.5 | 1.4 |

**^¥^Source**: 2019 World Population Prospects

## Age-specific HIV-deleted Mortality Rates

### HIV-deleted mortality rates calculations

In EMOD, we modelled HIV cause-deleted mortality rates in the background and HIV transmission and related morality rates in the foreground. To calculate the HIV deleted mortality, we first investigated all-cause mortality trends between 1960 and 2000 between countries with and without widespread HIV-AIDS crises. Countries without the epidemic demonstrated an exponential decline in mortality, while those grappling with the crisis experienced an exponential decrease interrupted by a sudden spike in the 1980s. We assumed the difference between these two curves (i.e., the spike) is due to the impact of the HIV-AIDS epidemic. Taking Kenya as an example, we fitted an exponential curve from 1970 to 1980 to represent the cause-deleted mortality. We then conducted a check on the population demographics generated by EMOD, ensuring that the age structure of the population simulated through both cause-deleted mortality and simulated HIV transmission aligns with the UN WPP’s population projections post-1980.

### Table S6a. Age-specific HIV deleted mortality rates in Kenya 1950-2049 by gender ^¥^

|  |  | **Age-specific mortality rates (%)** | | | | | | |
| --- | --- | --- | --- | --- | --- | --- | --- | --- |
| **Sex** | **Year** | **15-19** | **20-24** | **25-29** | **30-34** | **35-39** | **40-44** | **45-49** |
| **Women** | **1997.5** | 0.136 | 0.191 | 0.244 | 0.294 | 0.361 | 0.453 | 0.542 |
|  | **2002.5** | 0.116 | 0.167 | 0.215 | 0.259 | 0.321 | 0.408 | 0.493 |
|  | **2007.5** | 0.100 | 0.145 | 0.188 | 0.229 | 0.286 | 0.367 | 0.449 |
|  | **2012.5** | 0.085 | 0.127 | 0.166 | 0.202 | 0.254 | 0.331 | 0.408 |
|  | **2017.5** | 0.073 | 0.110 | 0.145 | 0.178 | 0.226 | 0.298 | 0.371 |
|  | **2022.5** | 0.063 | 0.096 | 0.128 | 0.158 | 0.201 | 0.268 | 0.338 |
|  | **2027.5** | 0.054 | 0.084 | 0.112 | 0.139 | 0.179 | 0.241 | 0.307 |
|  | **2032.5** | 0.046 | 0.073 | 0.099 | 0.123 | 0.160 | 0.217 | 0.280 |
|  | **2037.5** | 0.039 | 0.064 | 0.087 | 0.108 | 0.142 | 0.196 | 0.254 |
|  | **2042.5** | 0.034 | 0.055 | 0.076 | 0.096 | 0.126 | 0.176 | 0.231 |
| **Men** | **1997.5** | 0.165 | 0.253 | 0.288 | 0.345 | 0.433 | 0.551 | 0.713 |
|  | **2002.5** | 0.142 | 0.219 | 0.251 | 0.304 | 0.385 | 0.494 | 0.648 |
|  | **2007.5** | 0.121 | 0.189 | 0.219 | 0.267 | 0.343 | 0.443 | 0.589 |
|  | **2012.5** | 0.104 | 0.163 | 0.191 | 0.235 | 0.305 | 0.398 | 0.535 |
|  | **2017.5** | 0.089 | 0.141 | 0.166 | 0.207 | 0.271 | 0.357 | 0.486 |
|  | **2022.5** | 0.076 | 0.122 | 0.145 | 0.182 | 0.241 | 0.320 | 0.441 |
|  | **2027.5** | 0.065 | 0.106 | 0.126 | 0.160 | 0.215 | 0.287 | 0.401 |
|  | **2032.5** | 0.056 | 0.091 | 0.110 | 0.141 | 0.191 | 0.258 | 0.364 |
|  | **2037.5** | 0.048 | 0.079 | 0.096 | 0.124 | 0.170 | 0.231 | 0.331 |
|  | **2042.5** | 0.041 | 0.068 | 0.083 | 0.109 | 0.151 | 0.208 | 0.300 |

**^¥^Source**: 2019 World Population Prospects

### Table S6b. Age-specific mortality rates in South Africa 1950-2049 by gender^¥^

|  |  | **Age-specific mortality rates (%)** | | | | | | |
| --- | --- | --- | --- | --- | --- | --- | --- | --- |
| **Sex** | **Year** | **15-19** | **20-24** | **25-29** | **30-34** | **35-39** | **40-44** | **45-49** |
| **Women** | **1997.5** | 0.116 | 0.163 | 0.215 | 0.263 | 0.351 | 0.482 | 0.702 |
|  | **2002.5** | 0.096 | 0.134 | 0.179 | 0.222 | 0.302 | 0.424 | 0.629 |
|  | **2007.5** | 0.079 | 0.111 | 0.150 | 0.187 | 0.260 | 0.373 | 0.563 |
|  | **2012.5** | 0.066 | 0.092 | 0.125 | 0.158 | 0.223 | 0.328 | 0.504 |
|  | **2017.5** | 0.054 | 0.076 | 0.105 | 0.133 | 0.192 | 0.288 | 0.451 |
|  | **2022.5** | 0.045 | 0.063 | 0.087 | 0.113 | 0.165 | 0.254 | 0.404 |
|  | **2027.5** | 0.037 | 0.052 | 0.073 | 0.095 | 0.142 | 0.223 | 0.362 |
|  | **2032.5** | 0.031 | 0.043 | 0.061 | 0.080 | 0.122 | 0.196 | 0.324 |
|  | **2037.5** | 0.025 | 0.035 | 0.051 | 0.068 | 0.105 | 0.172 | 0.290 |
|  | **2042.5** | 0.021 | 0.029 | 0.042 | 0.057 | 0.090 | 0.152 | 0.260 |
| **Men** | **1997.5** | 0.177 | 0.312 | 0.361 | 0.437 | 0.594 | 0.872 | 1.264 |
|  | **2002.5** | 0.169 | 0.281 | 0.327 | 0.395 | 0.541 | 0.803 | 1.177 |
|  | **2007.5** | 0.173 | 0.254 | 0.296 | 0.358 | 0.494 | 0.739 | 1.095 |
|  | **2012.5** | 0.156 | 0.230 | 0.268 | 0.324 | 0.451 | 0.681 | 1.019 |
|  | **2017.5** | 0.141 | 0.207 | 0.243 | 0.294 | 0.411 | 0.627 | 0.949 |
|  | **2022.5** | 0.127 | 0.187 | 0.220 | 0.266 | 0.375 | 0.578 | 0.883 |
|  | **2027.5** | 0.115 | 0.169 | 0.199 | 0.241 | 0.342 | 0.532 | 0.822 |
|  | **2032.5** | 0.103 | 0.153 | 0.180 | 0.218 | 0.312 | 0.490 | 0.765 |
|  | **2037.5** | 0.093 | 0.138 | 0.163 | 0.197 | 0.285 | 0.451 | 0.713 |
|  | **2042.5** | 0.082 | 0.124 | 0.148 | 0.179 | 0.260 | 0.415 | 0.663 |

**^¥^Source**: 2012 World Population Prospects

### Table S6C. Age-specific HIV-deleted mortality rates in Zimbabwe 1950-2049 by gender

|  |  | **Age-specific mortality rates (%)** | | | | | | |
| --- | --- | --- | --- | --- | --- | --- | --- | --- |
| **Sex** | **Year** | **15-19** | **20-24** | **25-29** | **30-34** | **35-39** | **40-44** | **45-49** |
| **Women** | **1997.5** | 0.130 | 0.205 | 0.293 | 0.369 | 0.470 | 0.580 | 0.692 |
|  | **2002.5** | 0.104 | 0.172 | 0.251 | 0.321 | 0.416 | 0.521 | 0.631 |
|  | **2007.5** | 0.084 | 0.144 | 0.216 | 0.279 | 0.368 | 0.467 | 0.574 |
|  | **2012.5** | 0.068 | 0.121 | 0.186 | 0.243 | 0.325 | 0.420 | 0.523 |
|  | **2017.5** | 0.055 | 0.102 | 0.159 | 0.212 | 0.288 | 0.377 | 0.477 |
|  | **2022.5** | 0.044 | 0.085 | 0.137 | 0.184 | 0.254 | 0.338 | 0.434 |
|  | **2027.5** | 0.035 | 0.071 | 0.118 | 0.160 | 0.225 | 0.303 | 0.395 |
|  | **2032.5** | 0.029 | 0.060 | 0.101 | 0.140 | 0.199 | 0.272 | 0.360 |
|  | **2037.5** | 0.023 | 0.050 | 0.087 | 0.121 | 0.176 | 0.244 | 0.328 |
|  | **2042.5** | 0.018 | 0.042 | 0.075 | 0.106 | 0.156 | 0.219 | 0.299 |
| **Men** | **1997.5** | 0.185 | 0.285 | 0.333 | 0.407 | 0.521 | 0.666 | 0.869 |
|  | **2002.5** | 0.154 | 0.240 | 0.283 | 0.351 | 0.456 | 0.590 | 0.784 |
|  | **2007.5** | 0.128 | 0.203 | 0.241 | 0.302 | 0.399 | 0.522 | 0.708 |
|  | **2012.5** | 0.107 | 0.171 | 0.205 | 0.261 | 0.349 | 0.463 | 0.638 |
|  | **2017.5** | 0.089 | 0.144 | 0.174 | 0.225 | 0.305 | 0.410 | 0.576 |
|  | **2022.5** | 0.074 | 0.122 | 0.148 | 0.194 | 0.267 | 0.363 | 0.520 |
|  | **2027.5** | 0.062 | 0.103 | 0.126 | 0.167 | 0.234 | 0.321 | 0.469 |
|  | **2032.5** | 0.052 | 0.087 | 0.107 | 0.144 | 0.204 | 0.285 | 0.423 |
|  | **2037.5** | 0.043 | 0.073 | 0.091 | 0.124 | 0.179 | 0.252 | 0.381 |
|  | **2042.5** | 0.036 | 0.062 | 0.078 | 0.107 | 0.156 | 0.223 | 0.344 |

**^¥^Source**: 2019 World Population Prospects

## Model fit to age-specific and overall prevalence from population-based surveys by sex

### Figure S1a Model fit to age-specific and overall prevalence from population-based surveys by sex in Kenya


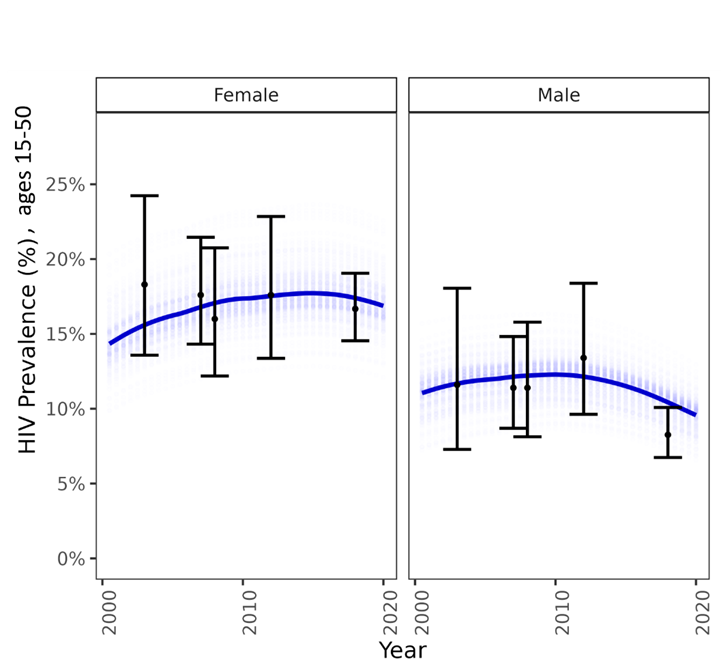


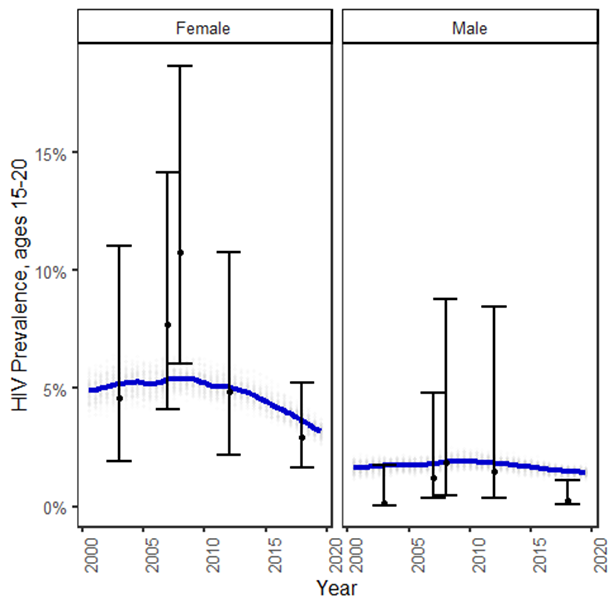

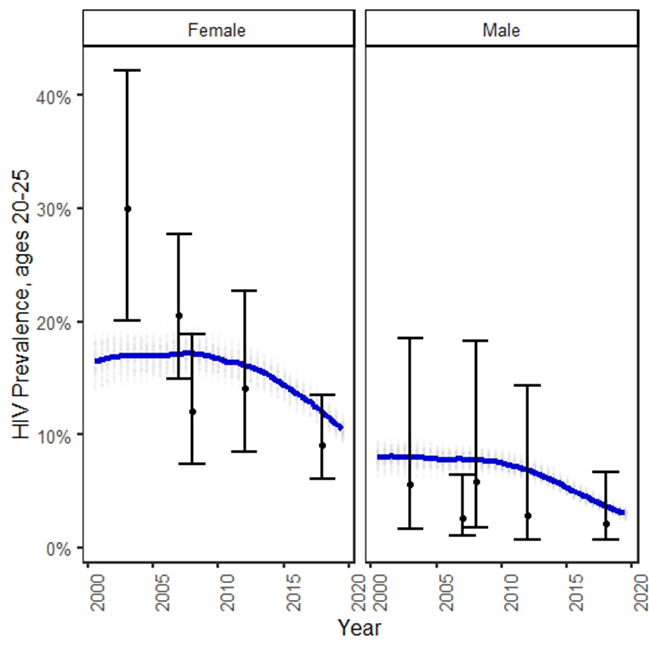

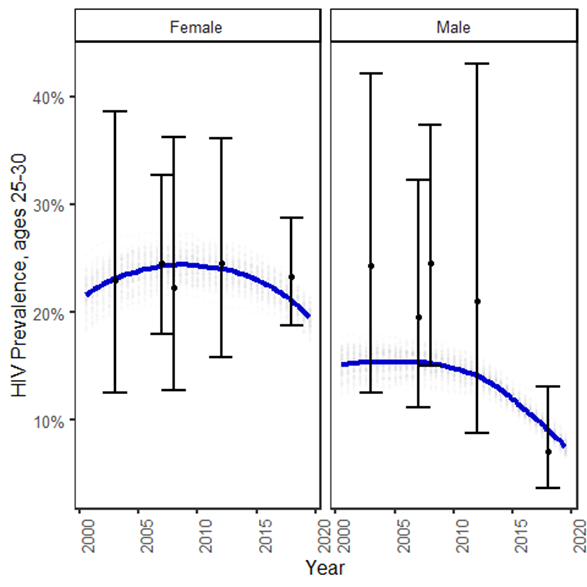


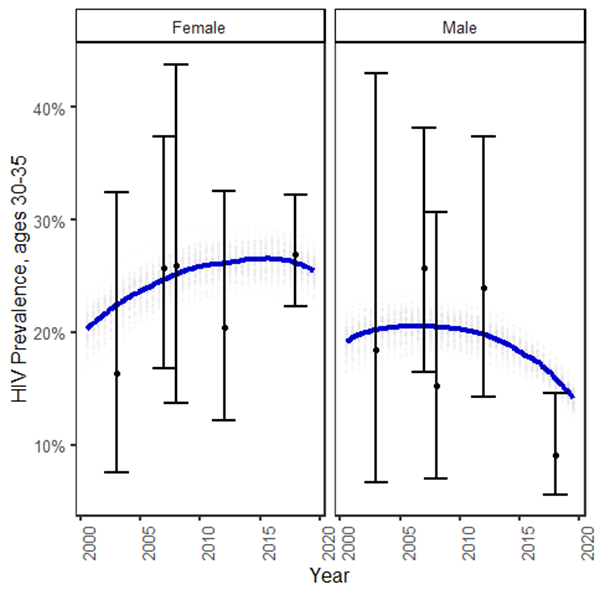


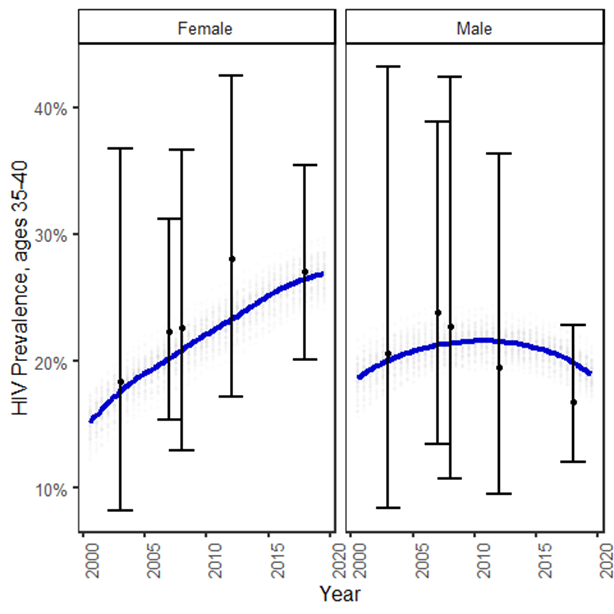


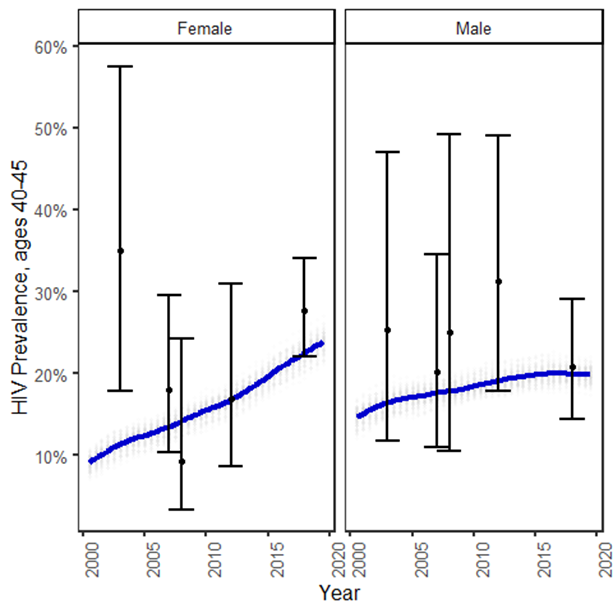


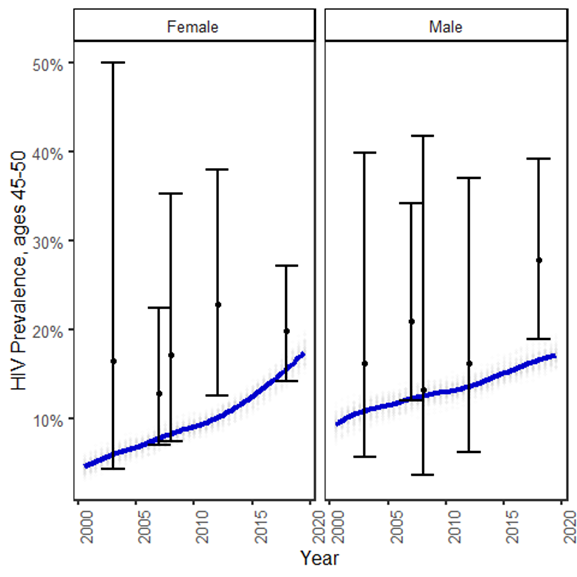


* Blue curves refer to the LOESS line fitting to the 100 simulations; the error bars refer to the empirical estimates and 95% confidence intervals for HIV prevalence obtained from Kenya Demographic and Health Surveys and Kenya AIDS Indicator Surveys.

### Figure S1b Model fit to age-specific and overall prevalence from population-based surveys by sex in South Africa


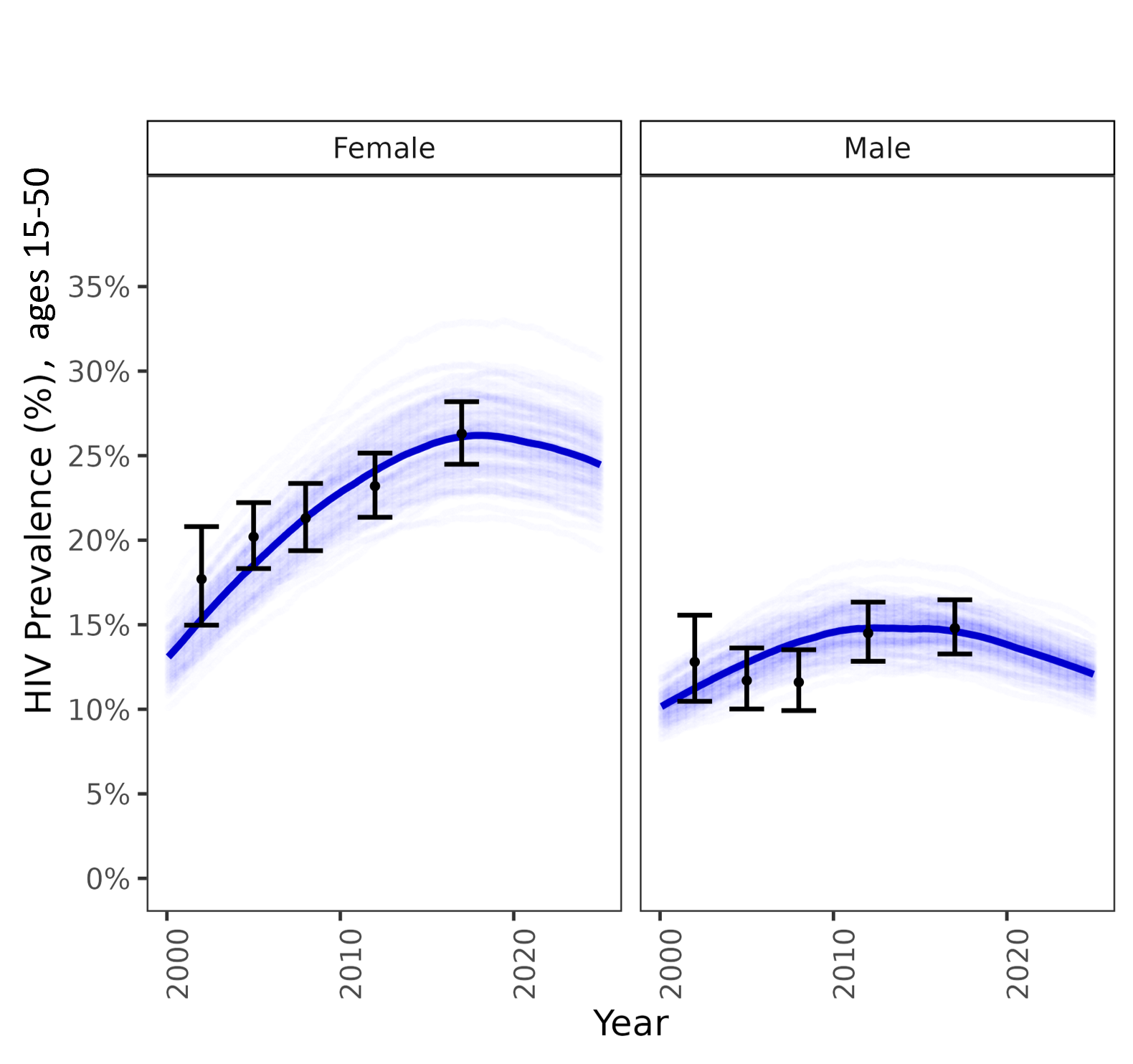


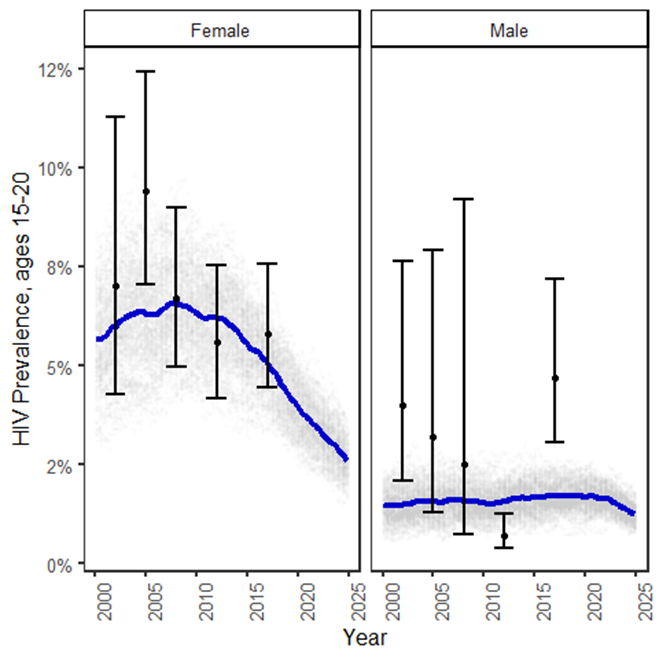


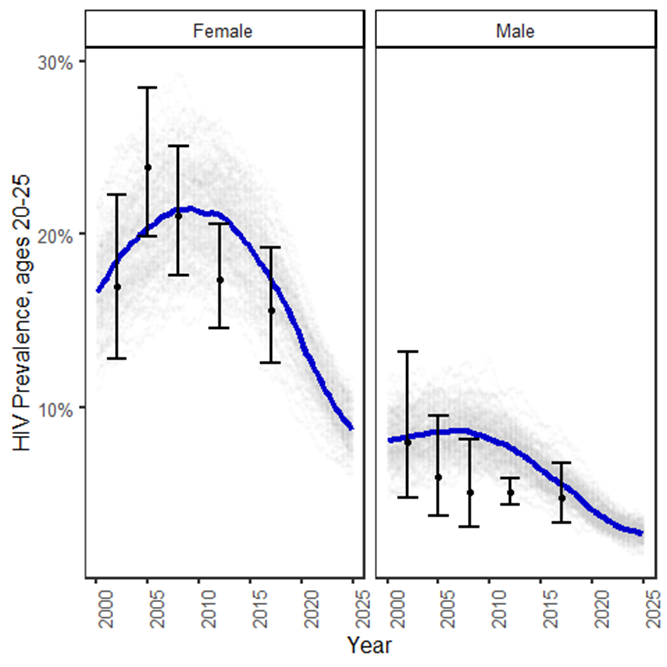


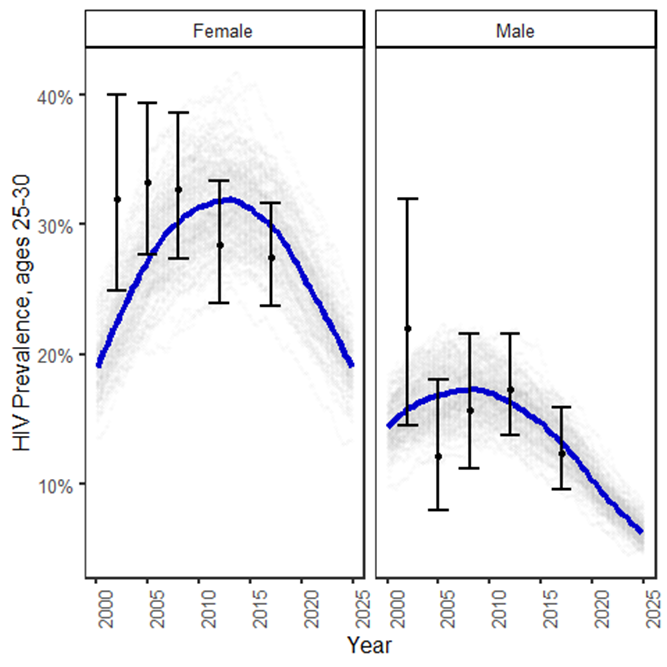


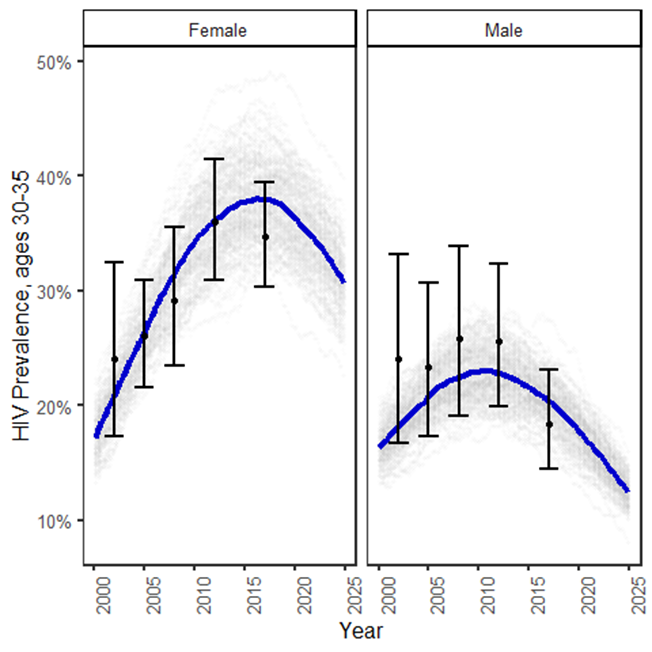


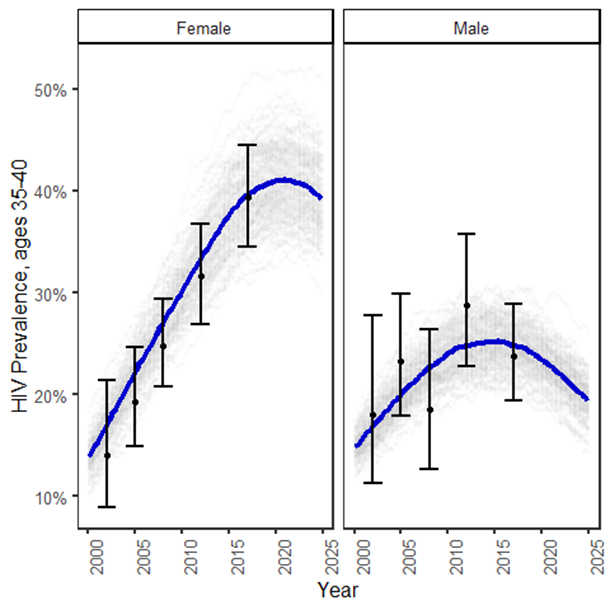


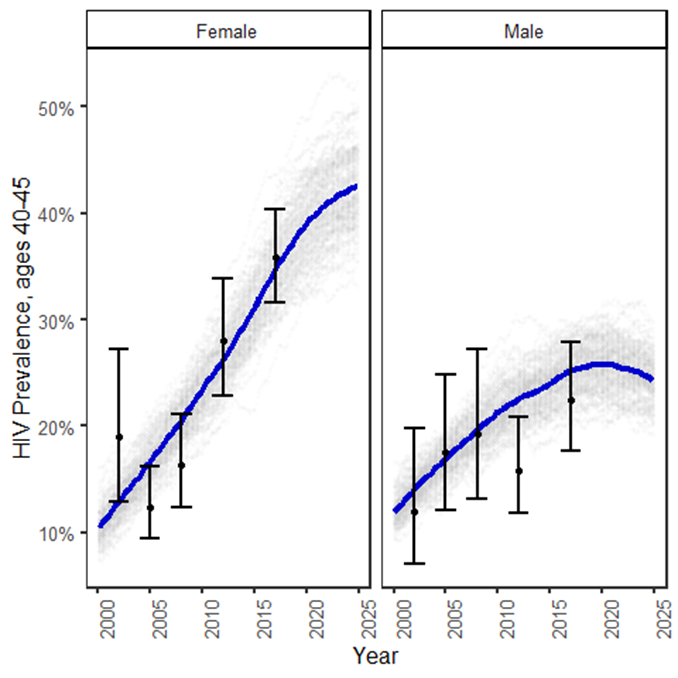


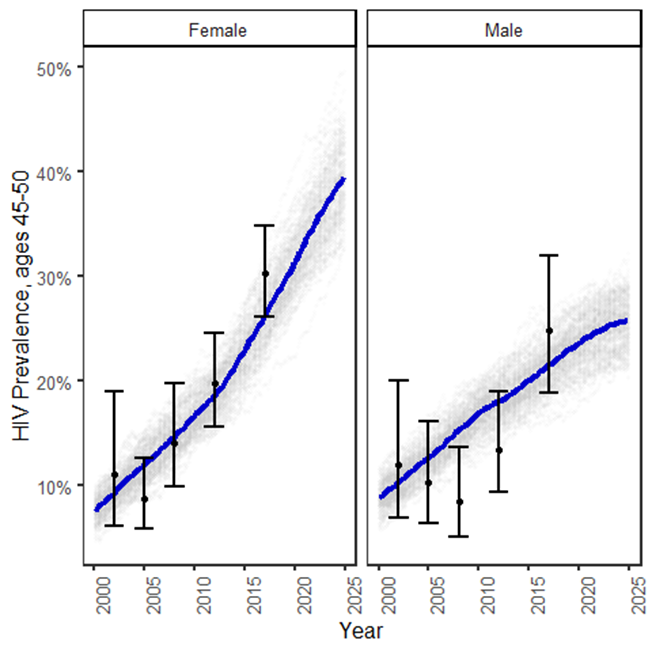


* Blue curves refer to the LOESS average fitting to the 100 simulations; the error bars refer to the empirical estimates and 95% confidence intervals for HIV prevalence obtained from South African National HIV Prevalence, Incidence and Behaviour Surveys (2002, 2005, 2008, 2012 and 2017) from the Human Sciences Research Council (HSRC)

### Figure S1c. Model fit to age-specific and overall prevalence from population-based surveys by sex in Zimbabwe


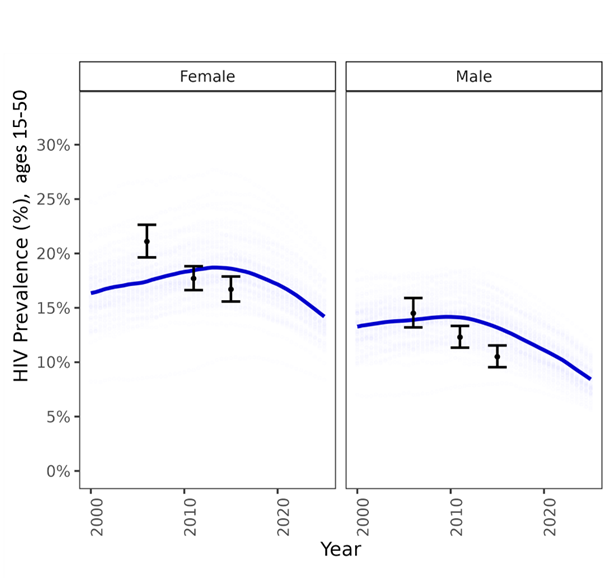


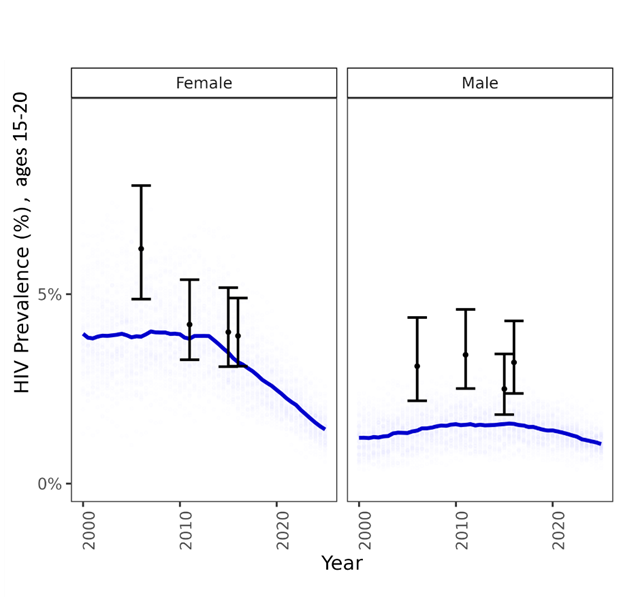


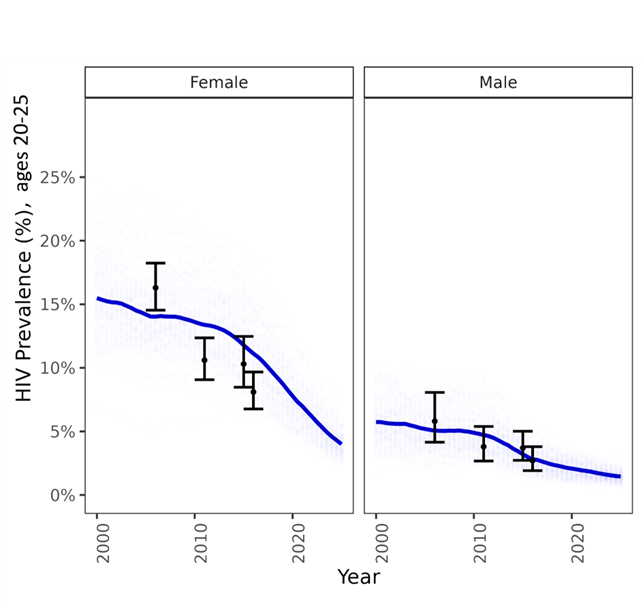


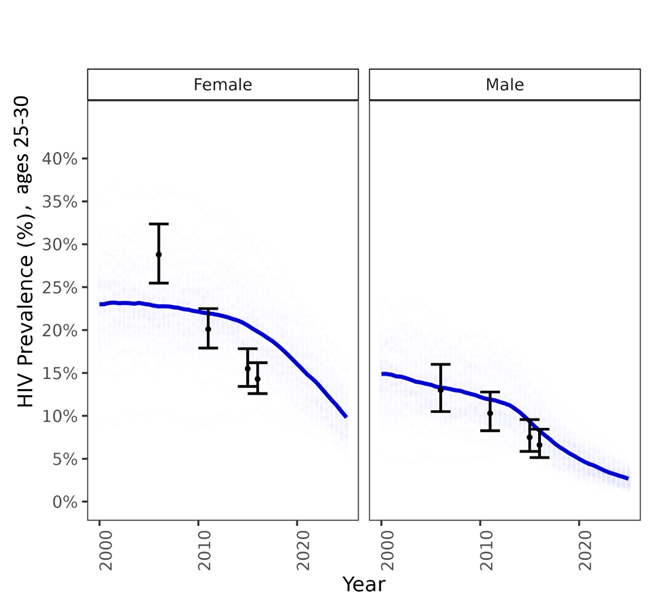


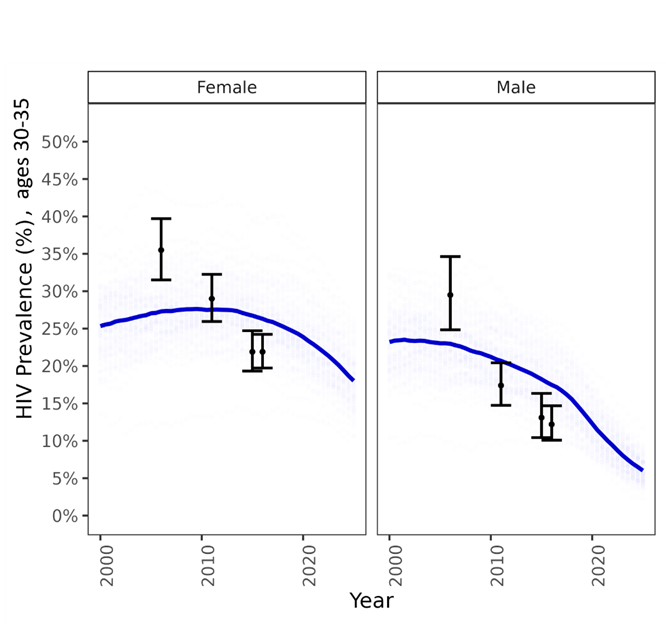


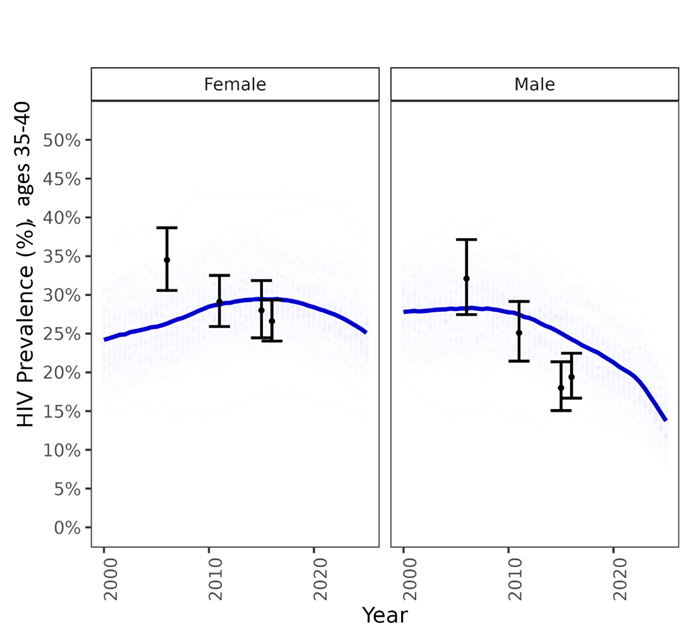


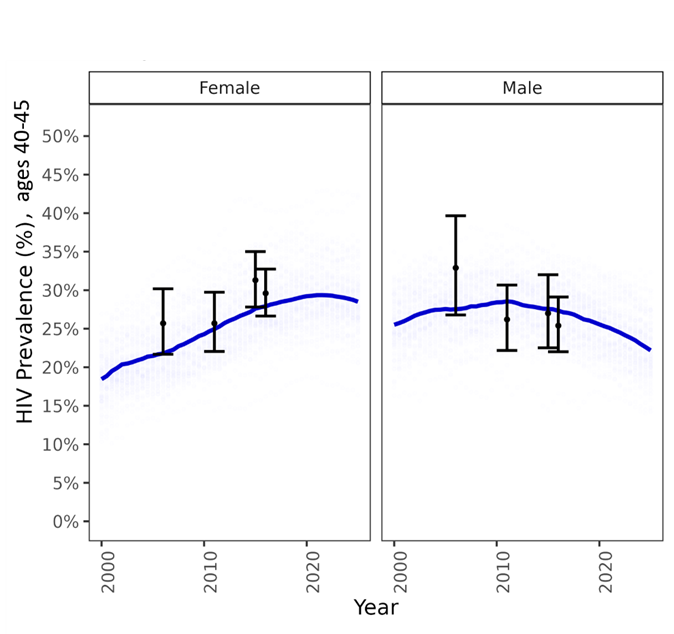


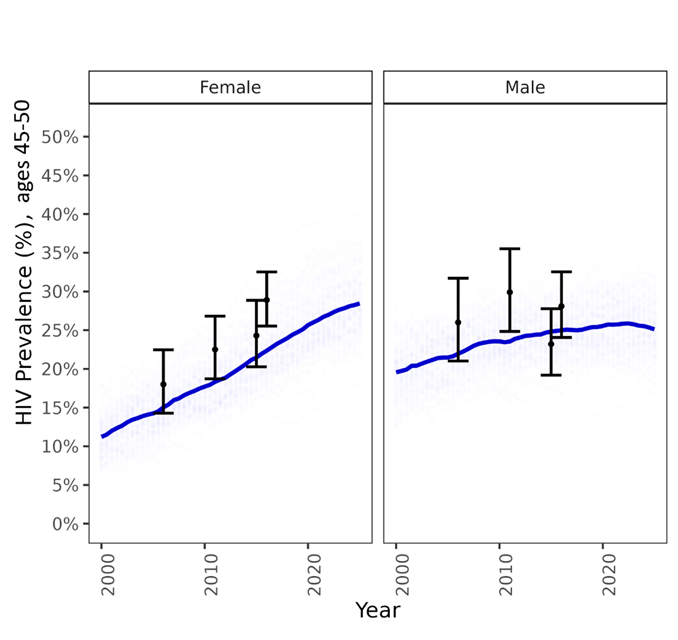


* Blue curves refer to the LOESS average fitting to the 100 simulations; the error bars refer to the empirical estimates and 95% confidence intervals for HIV prevalence obtained from PHIA Surveys

## Model fit to age-specific and overall prevalence from population-based surveys by sex

### Figure S2a Model fit to age-specific and overall ART coverage from population-based surveys by sex in Kenya


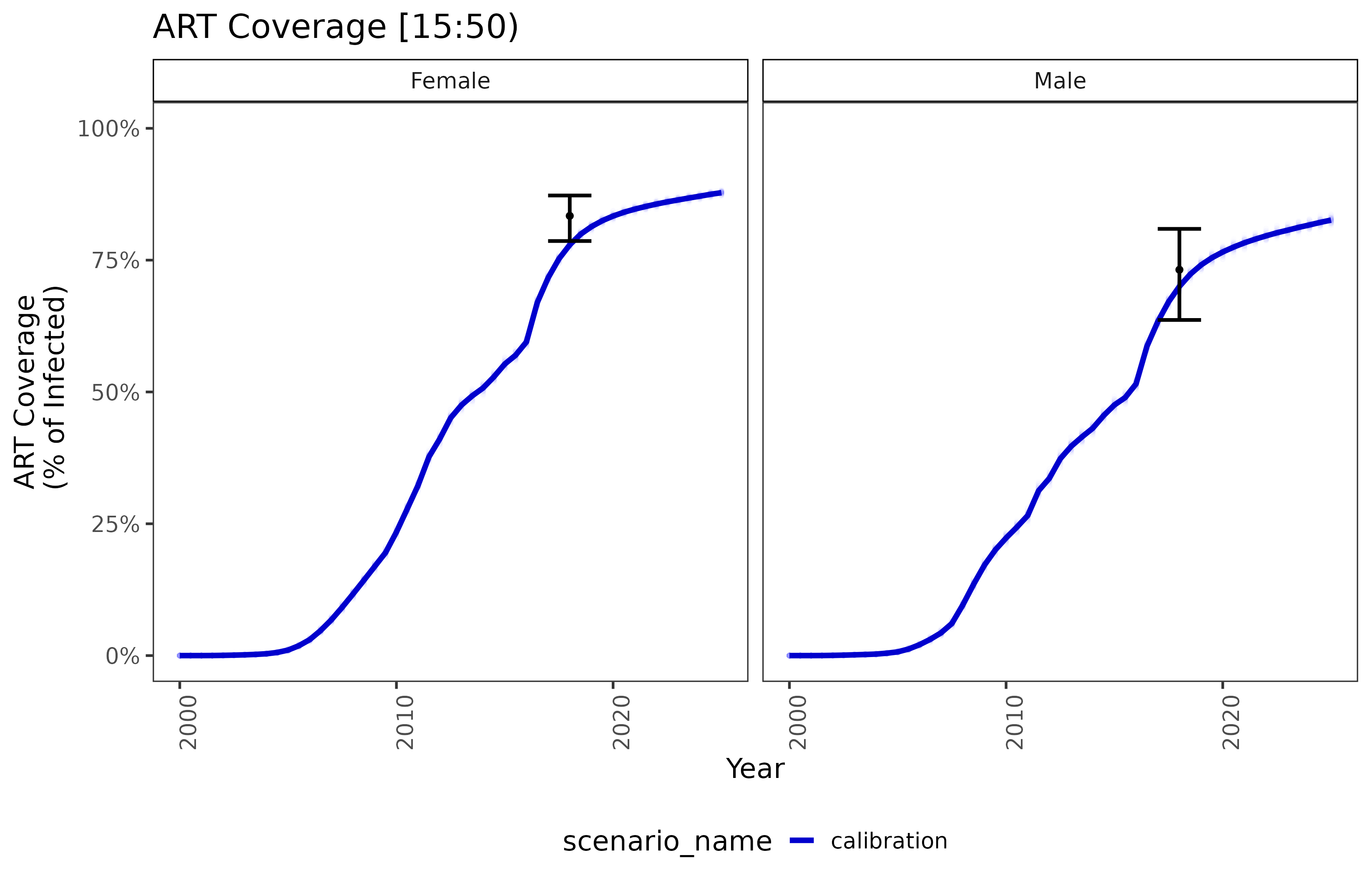


### Figure S2b Model fit to age-specific and overall ART coverage from population-based surveys by sex in South Africa


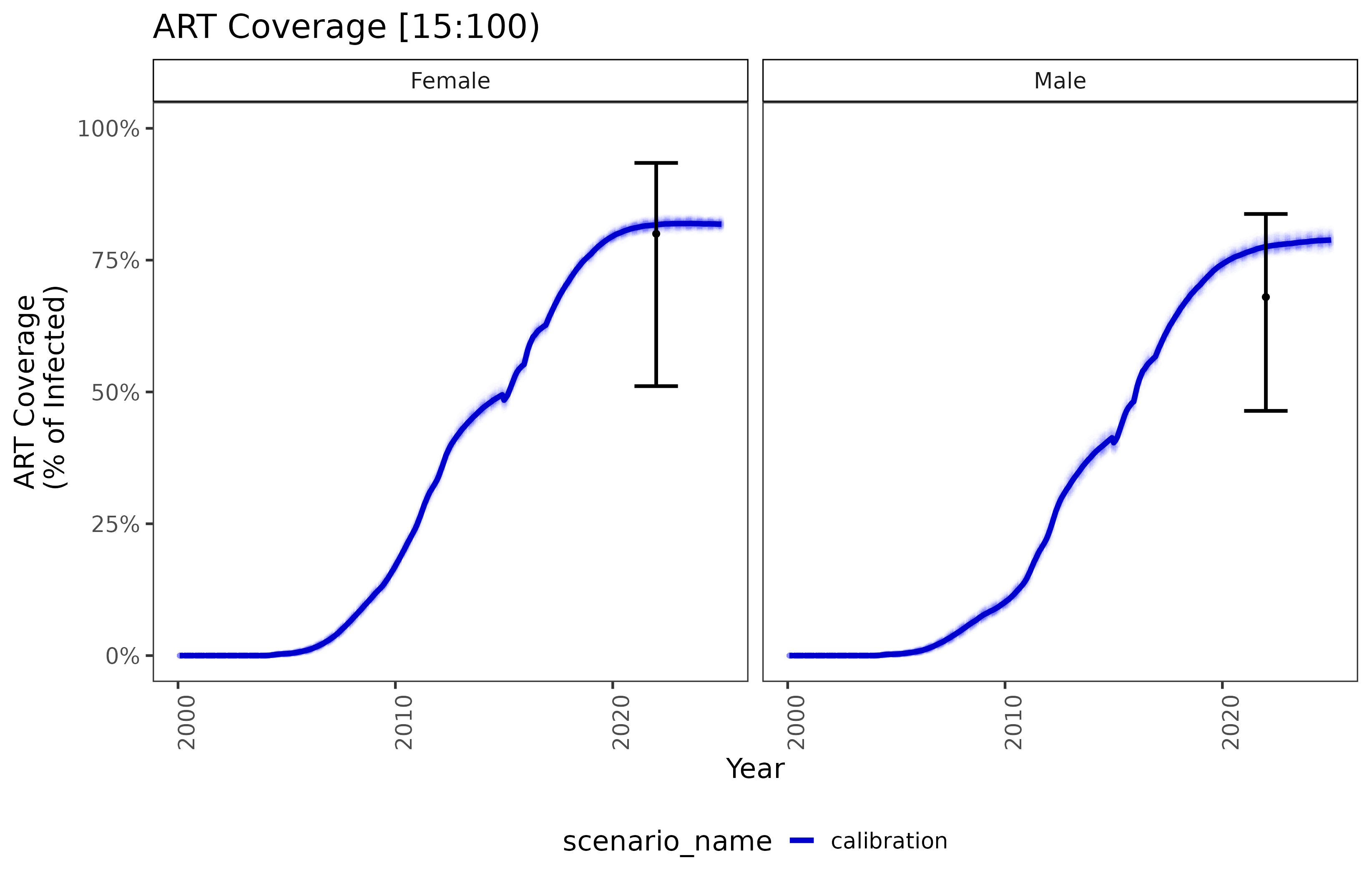


### Figure S2c Model fit to age-specific and overall ART coverage from population-based surveys by sex in Zimbabwe


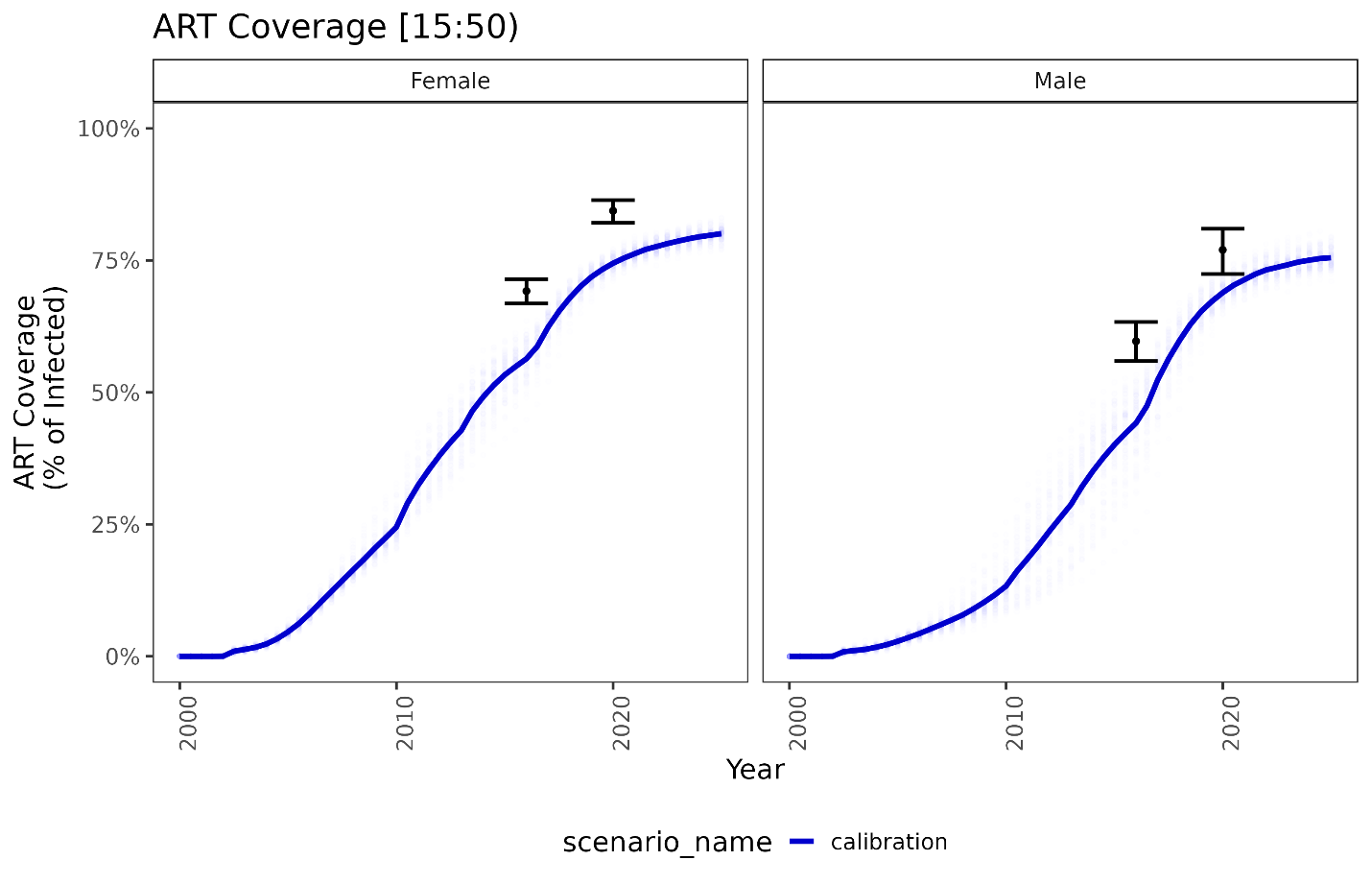


# Model Overview and Parameters

*Model initialization*

The simulations begin prior to the start of infection in the year 1960 to allow sufficient time for the epidemic to burn-in. During this time, individuals with demographic properties specified in the demographics file begin forming relationships; relationship formation rates for each gender and relationship type are updated daily using a relationship flow algorithm. Adjustment of pair formation entry rates is terminated at a specific timepoint (e.g., 1975) and the rates are fixed at that value for the remainder of the simulation. The age profile of the population is initialized using demographic data including population age distribution or age-specific fertility rates. A prior analysis of our model evaluated the age/sex pairings, partnership length and other sexual network characteristics and confirmed that these outputs reached equilibrium within 20 years, prior to the introduction of HIV into the model: <https://ieeexplore.ieee.org/abstract/document/6426573>. (1) HIV infections are seeded in 1980 and affect a certain proportion of the population based on the age and gender distribution reported from historical data. ART intervention is introduced in the year 2012. Eligible individuals enroll in ART based on historical eligibility criteria based on CD4 count, which changes over time based on WHO guidelines for ART initiation until the implementation of universal ART, which is assumed to remain the same until the end of the simulations (year 2050). Calibration and validation processes are performed to refine the initialization and ensure that the model aligns with observed HIV dynamics in the target population. (2, 3)

The following provides a more detailed description of the model initialization process: <https://docs.idmod.org/projects/emod-hiv/en/latest/sti-model-relationships.html>. (2)

*Modelled time step*

The model is implemented using a monthly time step, aggregating events and changes over the course of each month. Monthly updated information can then be ascertained regarding a range of activities and occurrences, including sexual mixing, relationship formation, stages of HIV infection, HIV testing and its results, and PrEP initiation and discontinuation.

*PrEP cascade*

HIV-negative individuals aged 18 to 49 years were eligible for PrEP if testing negative at the time of initiation or continuation, and if sexually active with at least one partner. PrEP discontinuation occurred if individuals are lost to follow-up or no longer met the eligibility criteria (i.e., an individual turned 50 years old, all partnerships end, or tested HIV-positive). Individuals who stopped PrEP can re-start at any time if they meet the eligibility criteria (i.e. start a new partnership).

## Model Parameters

### Table S7a. Select model parameters used to fit the EMOD-HIV transmission model to survey data on prevalence and ART coverage from Kenya.

| **Parameter** | **Parameter Description** | **Fitted median** | **(IQR)** |
| --- | --- | --- | --- |
| ARTLinkMax | Maximum probability of linkage to ART | 0.999 | (0.978, 1.000) |
| ARTLinkMid | Year of ART linkage (given eligibility), that is, time of the inflection point in the sigmoid trend. | 2003.008 | (2002.920, 2003.837) |
| AcuteDurationMonths | The time since infection, in months, over which the Acute_Stage_Infectivity_Multiplier is applied to coital acts occurring in that time period. | 1.000 | (1.000, 1.331) |
| CircumcisionReducedAcquire | The reduction of susceptibility to STI by voluntary male medical circumcision (VMMC). | 0.600 | (0.598, 0.600) |
| Homa_BayInfrmlCondomsMax | Maximum rate of condom use in informal relationships in Homa Bay | 0.231 | (0.230, 0.233) |
| Homa_BayLOWRisk | Proportion of the population that is low-risk in Homa Bay | 0.557 | (0.549, 0.559) |
| Homa_BayTrnsCondomsMax | Maximum rate of condom use in transitory relationships in Homa Bay | 0.244 | (0.232, 0.245) |
| InfmrlFormRate | Informal relationship formation rate | 0.000 | (0.000, 0.000) |
| InfrmlCondomMid | Year midpoint of logistic scale-up of condom use in informal relationships | 1993.007 | (1991.629, 1998.680) |
| InfrmlCondomRate | Rate of logistic scale-up of condom use in informal relationships | 2.661 | (1.953, 2.909) |
| InfrmlCondomsMax | Maximum rate of condom use in informal relationships | 0.216 | (0.215, 0.217) |
| InfrmlDurHet | Heterogeneity in duration of informal relationships | 0.750 | (0.750, 0.750) |
| KisiiInfrmlCondomsMax | Maximum rate of condom use in informal relationships in Kisii | 0.231 | (0.226, 0.232) |
| KisiiLOWRisk | Proportion of the population that is low-risk in Kisii | 0.938 | (0.935, 0.939) |
| KisiiTrnsCondomsMax | Maximum rate of condom use in transitory relationships in Kisii | 0.375 | (0.369, 0.376) |
| KisumuInfrmlCondomsMax | Maximum rate of condom use in informal relationships in Kisumu | 0.182 | (0.181, 0.183) |
| KisumuLOWRisk | Proportion of the population that is low-risk in Kisumu | 0.764 | (0.762, 0.765) |
| KisumuTrnsCondomsMax | Maximum rate of condom use in transitory relationships in Kisumu | 0.338 | (0.337, 0.347) |
| LogBaseInfectivity | The probability of transmission when none of the transmission multipliers apply to a particular coital act. | 0.002 | (0.002, 0.002) |
| MaleToFemaleOld | Male-to-female relative risk of infection among older individuals | 2.012 | (1.506, 2.132) |
| MaleToFemaleYoung | Male-to-female relative risk of infection among young individuals | 1.116 | (1.000, 1.242) |
| MaxInfrmlFLOW | Maximum number of informal relationships among low-risk females | 1.311 | (1.255, 1.315) |
| MaxInfrmlFMED | Maximum number of informal relationships among medium-risk females | 2.050 | (2.002, 2.351) |
| MaxInfrmlMLOW | Maximum number of informal relationships among low-risk males | 1.165 | (1.159, 1.203) |
| MaxInfrmlMMED | Maximum number of informal relationships among medium-risk males | 2.530 | (2.442, 2.735) |
| MaxMrtlFMED | Maximum number of marital relationship among medium-risk females | 1.139 | (1.136, 1.159) |
| MaxMrtlMMED | Maximum number of marital relationship among medium-risk males | 1.302 | (1.268, 1.316) |
| MaxTrnsFLOW | Maximum number of transitory relationships among low-risk females | 1.599 | (1.580, 1.625) |
| MaxTrnsFMED | Maximum number of transitory relationships among medium-risk females | 2.943 | (2.891, 3.000) |
| MaxTrnsMLOW | Maximum number of transitory relationships among low-risk males | 1.599 | (1.588, 1.677) |
| MaxTrnsMMED | Maximum number of transitory relationships among medium-risk males | 2.557 | (2.475, 2.879) |
| MigoriInfrmlCondomsMax | Maximum rate of condom use in informal relationships in Migori | 0.186 | (0.185, 0.189) |
| MigoriLOWRisk | Proportion of the population that is low-risk in Migori | 0.795 | (0.793, 0.797) |
| MigoriTrnsCondomsMax | Maximum rate of condom use in transitory relationships in Migori | 0.249 | (0.248, 0.257) |
| MrtlCondomMax | Maximum rate of condom use in marital relationships | 0.192 | (0.191, 0.193) |
| MrtlCondomMid | Year midpoint of logistic scale-up of condom use in marital relationships | 2003.131 | (1999.017, 2005.000) |
| MrtlCondomRate | Rate of logistic scale-up of condom use in marital relationships | 2.971 | (2.595, 3.000) |
| MrtlFormRate | Marital relationship formation rate | 0.000 | (0.000, 0.000) |
| NyamiraInfrmlCondomsMax | Maximum rate of condom use in informal relationships in Nyamira | 0.096 | (0.093, 0.097) |
| NyamiraLOWRisk | Proportion of the population that is low-risk in Nyamira | 0.902 | (0.900, 0.910) |
| NyamiraTrnsCondomsMax | Maximum rate of condom use in transitory relationships in Nyamira | 0.309 | (0.304, 0.313) |
| PrExInfrmlFemLOW | Probability of potential for extra-relational informal relationship among low-risk females | 0.366 | (0.364, 0.371) |
| PrExInfrmlFemMED | Probability of potential for extra-relational informal relationship among medium-risk females | 0.401 | (0.401, 0.407) |
| PrExInfrmlMaleLOW | Probability of potential for extra-relational informal relationship among low-risk males | 0.244 | (0.222, 0.249) |
| PrExInfrmlMaleMED | Probability of potential for extra-relational informal relationship among medium-risk males | 0.382 | (0.379, 0.389) |
| PrExTrnsFemLOW | Probability of potential for extra-relational transitory relationship among low-risk females | 0.033 | (0.033, 0.033) |
| PrExTrnsFemMED | Probability of potential for extra-relational transitory relationship among medium-risk females | 0.460 | (0.450, 0.468) |
| PrExTrnsMaleLOW | Probability of potential for extra-relational transitory relationship among low-risk males | 0.330 | (0.328, 0.332) |
| PrExTrnsMaleMED | Probability of potential for extra-relational transitory relationship among medium-risk males | 0.593 | (0.589, 0.611) |
| PreARTLinkMax | Maximum probability of linkage to pre-ART care | 0.715 | (0.708, 0.737) |
| PreARTLinkMid | Year midpoint of logistic scale-up of pre-ART linkage | 1996.709 | (1995.685, 1998.896) |
| PreARTLinkMin | Minimum probability of linkage to pre-ART care | 0.435 | (0.416, 0.442) |
| RiskAssortivity | Risk assortivity | 0.663 | (0.648, 0.673) |
| SeedYrHigh | Seed year | 1986.562 | (1982.000, 1988.000) |
| SexualDebutAgeFemaleWeibullHeterogeneity | Heterogeneity parameter of Weibull distribution of female age of sexual debut | 0.086 | (0.083, 0.086) |
| SexualDebutAgeFemaleWeibullScale | Scale parameter of Weibull distribution of female age of sexual debut | 16.013 | (15.540, 16.038) |
| SexualDebutAgeMaleWeibullHeterogeneity | Heterogeneity parameter of Weibull distribution of male age of sexual debut | 0.040 | (0.040, 0.040) |
| SexualDebutAgeMaleWeibullScale | Scale parameter of Weibull distribution of male age of sexual debut | 15.708 | (15.155, 16.090) |
| SiayaInfrmlCondomsMax | Maximum rate of condom use in informal relationships in Siaya | 0.160 | (0.148, 0.161) |
| SiayaLOWRisk | Proportion of the population that is low-risk in Siaya | 0.729 | (0.722, 0.731) |
| SiayaTrnsCondomsMax | Maximum rate of condom use in transitory relationships in Siaya | 0.300 | (0.293, 0.304) |
| TrnsCondomMax | Maximum rate of condom use in transitory relationships | 0.243 | (0.242, 0.253) |
| TrnsCondomMid | Year midpoint of logistic scale-up of condom use in transitory relationships | 1997.960 | (1996.962, 1999.273) |
| TrnsCondomRate | Rate of logistic scale-up of condom use in transitory relationships | 0.999 | (0.978, 1.000) |
| TrnsFormRate | Transitory relationship formation rate | 2003.008 | (2002.920, 2003.837) |

* Median and interquartile ranges (IQRs) reported for all dynamic parameters used in the calibration process from 100 best-fitting parameter sets. †

###

### Table S7b. Select model parameters used to fit the EMOD-HIV transmission model to survey data on prevalence and ART coverage from South Africa.

| **Parameter** | **Description** | **Fitted Median** | **(IQR)** |
| --- | --- | --- | --- |
| ART Link Max | Maximum probability of linkage to ART | 1.000 | (0.997, 1.000) |
| ART Link Mid | Year of ART linkage (given eligibility), that is, time of the inflection point in the sigmoid trend. | 2,005.96 | (2,005.86, 2,006.08) |
| All: Infmrl Condom | Modern condom usage rate in informal relationships across all locations | 0.61 | (0.58, 0.64) |
| All: LOW Risk | Proportion of the population that is low-risk | 0.936 | (0.930, 0.942) |
| All: Trns Condom | Modern condom usage rate in transitory relationships across all locations | 0.2 | (0.16, 0.23) |
| Base Infectivity | The probability of transmission when none of the transmission multipliers apply to a coital act (or when all multipliers are set to 1). | 0.0015 | (0.0015, 0.0016) |
| Circumcision Reduced Acquire | The reduction of susceptibility to STI by voluntary male medical circumcision (VMMC). | 0.6 |  |
| Infmrl Condoms Late | Modern condom usage rate in informal relationships, by county | 0.37 | (0.35, 0.39) |
| Infmrl Form Rate | Informal relationship formation rate | 0.0009 | (0.0008, 0.0009) |
| Infrml Condom Mid | Year midpoint of logistic scale-up of condom use in informal relationships | 1,998.27 | (1,997.81, 1,999.03) |
| Infrml Condom Rate | Rate of logistic scale-up of condom use in informal relationships | 2.03 | (1.89, 2.18) |
| Infrml Dur Het | Heterogeneity in duration of informal relationships | 0.693 | (0.675, 0.711) |
| Male To Female Old | Male-to-female relative risk of infection among older individuals | 2.33 | (2.22, 2.49) |
| Male To Female Young | Male-to-female relative risk of infection among young individuals | 2.97 | (2.74, 3.37) |
| Max Infmrl F LOW | Maximum number of informal relationships among low-risk females | 1.67 | (1.61, 1.72) |
| Max Infmrl F MED | Maximum number of informal relationships among medium-risk females | 0.92 | (0.89, 0.94) |
| Max Infmrl M LOW | Maximum number of informal relationships among low-risk males | 1.73 | (1.69, 1.76) |
| Max Infmrl M MED | Maximum number of informal relationships among medium-risk males | 0.75 | (0.70, 0.79) |
| Max Mrtl F ME | Maximum number of marital relationships among medium-risk females | 1.23 | (1.16, 1.27) |
| Max Mrtl M ME | Maximum number of marital relationships among medium-risk males | 0.93 | (0.91, 0.96) |
| Max Trns F LOW | Maximum number of transitory relationships among low-risk females | 1.5 | (1.48, 1.52) |
| Max Trns F MED | Maximum number of transitory relationships among medium-risk females | 3.1 | (3.06, 3.14) |
| Max Trns M LOW | Maximum number of transitory relationships among low-risk males | 1.49 | (1.46, 1.53) |
| Max Trns M MED | Maximum number of transitory relationships among medium-risk males | 3.02 | (2.88, 3.10) |
| Mrtl Condom Max | Maximum rate of condom use in marital relationships | 0.191 | (0.184, 0.211) |
| Mrtl Condom Mid | Year midpoint of logistic scale-up of condom use in marital relationships | 1,994.92 | (1,994.19, 1,995.27) |
| Mrtl Condom Rate | Rate of logistic scale-up of condom use in marital relationships | 3.6 | (3.49, 3.69) |
| Mrtl Form Rate | Marital relationship formation rate | 0.0001 | (0.0001, 0.0001) |
| Pr Ex Infmrl Fem LOW | Probability of potential for extra-relational informal relationship among low-risk females | 0.08 | (0.06, 0.10) |
| Pr Ex Infmrl Fem MED | Probability of potential for extra-relational informal relationship among medium-risk females | 0.391 | (0.381, 0.404) |
| Pr Ex Infmrl Male LOW | Probability of potential for extra-relational informal relationship among low-risk males | 0.46 | (0.42, 0.50) |
| Pr Ex Infmrl Male MED | Probability of potential for extra-relational informal relationship among medium-risk males | 0.379 | (0.370, 0.390) |
| Pr Ex Trns Fem LOW | Probability of potential for extra-relational transitory relationship among low-risk females | 0.066 | (0.058, 0.081) |
| Pr Ex Trns Fem MED | Probability of potential for extra-relational transitory relationship among medium-risk females | 0.58 | (0.56, 0.62) |
| Pr Ex Trns Male LOW | Probability of potential for extra-relational transitory relationship among low-risk males | 0.17 | (0.12, 0.19) |
| Pr Ex Trns Male MED | Probability of potential for extra-relational transitory relationship among medium-risk males | 0.59 | (0.58, 0.62) |
| PreART Link Max | Maximum probability of linkage to pre-ART care | 0.89 | (0.85, 0.96) |
| PreART Link Mid | Year midpoint of logistic scale-up of pre-ART linkage | 1,998.28 | (1,997.76, 1,998.72) |
| PreART Link Min | Minimum probability of linkage to pre-ART care | 0.63 | (0.62, 0.65) |
| Risk Assortivity | Risk assortivity | 0.47 | (0.44, 0.49) |
| SeedYr HIGH | Seed year | 1991 |  |
| Sexual Debut Age Female Weibull Heterogeneity | Heterogeneity parameter of Weibull distribution of female age of sexual debut | 0.062 | (0.051, 0.067) |
| Sexual Debut Age Female Weibull Scale | Scale parameter of Weibull distribution of female age of sexual debut | 16.49 | (16.34, 16.62) |
| Sexual Debut Age Male Weibull Heterogeneity | Heterogeneity parameter of Weibull distribution of male age of sexual debut | 0.043 | (0.038, 0.050) |
| Sexual Debut Age Male Weibull Scale | Scale parameter of Weibull distribution of male age of sexual debut | 16.47 | (16.24, 16.62) |
| Trns Condom Late | Modern condom usage rate in transitory relationships | 0.61 | (0.59, 0.64) |
| Trns Condom Mid | Year midpoint of logistic scale-up of condom use in transitory relationships | 2,006.77 | (2,006.08, 2,007.21) |
| Trns Condom Rate | Rate of logistic scale-up of condom use in transitory relationships | 1.94 | (1.84, 2.01) |
| Trns Form Rate | Transitory relationship formation rate | 0.0013 | (0.0013, 0.0014) |

* Median and interquartile ranges (IQRs) reported for all dynamic parameters used in the calibration process from 100 best-fitting parameter sets. †

### Table S7c. Select model parameters used to fit the EMOD-HIV transmission model to survey data on prevalence and ART coverage from Zimbabwe.

| **Parameter** | **Parameter Description** | **Fitted Median** | **(IQR)** |
| --- | --- | --- | --- |
| All: LOW Risk | Proportion of the population that is low-risk | 0.523 | (0.500, 0.540) |
| Base Infectivity | The probability of transmission when none of the transmission multipliers apply to a coital act | 0.0038 | (0.0035, 0.0040) |
| Demographic Coverage | Demographic coverage of the intervention | 0.193 | (0.185, 0.205) |
| HCT Uptake Post Debut Max | Maximum rate of HIV counseling and testing (HCT) post sexual debut | 0.34 | (0.28, 0.38) |
| HCT Uptake Post Debut Mid | Year midpoint of logistic scale-up of HIV counseling and testing (HCT) post sexual debut | 2,008.86 | (2,008.32, 2,009.70) |
| Informal Form Rate | Informal relationship formation rate | 0.0012 | (0.0011, 0.0013) |
| Infrml Condom Mid | Year midpoint of logistic scale-up of condom use in informal relationships | 1,998.13 | (1,997.41, 1,998.67) |
| Infrml Condom Rate | Rate of logistic scale-up of condom use in informal relationships | 3.85 | (3.73, 4.00) |
| Infrml Condoms Max | Maximum rate of condom use in informal relationships | 0.21 | (0.19, 0.26) |
| Male To Female Old | Male-to-female relative risk of infection among older individuals | 1.44 | (1.33, 1.66) |
| Male To Female Young | Male-to-female relative risk of infection among young individuals | 2.66 | (2.26, 2.83) |
| Marital Form Rate | Marital relationship formation rate | 0.00011 | (0.00009, 0.00014) |
| Max Infrml F LOW | Maximum number of informal relationships among low-risk females | 1.25 | (1.18, 1.32) |
| Max Infrml F MED | Maximum number of informal relationships among medium-risk females | 2.83 | (2.35, 3.15) |
| Max Infrml M LOW | Maximum number of informal relationships among low-risk males | 1.17 | (1.13, 1.21) |
| Max Infrml M MED | Maximum number of informal relationships among medium-risk males | 2.27 | (2.13, 2.46) |
| Max Mrtl F MED | Maximum number of marital relationships among medium-risk females | 1.18 | (1.12, 1.24) |
| Max Mrtl M MED | Maximum number of marital relationships among medium-risk males | 0.93 | (0.90, 0.98) |
| Max Trns F LOW | Maximum number of transitory relationships among low-risk females | 1.7 | (1.65, 1.73) |
| Max Trns F MED | Maximum number of transitory relationships among medium-risk females | 2.78 | (2.55, 3.03) |
| Max Trns M LOW | Maximum number of transitory relationships among low-risk males | 1.95 | (1.91, 2.00) |
| Max Trns M MED | Maximum number of transitory relationships among medium-risk males | 2.52 | (2.41, 2.60) |
| Mrtl Condom Max | Maximum rate of condom use in marital relationships | 0.18 | (0.18, 0.21) |
| Mrtl Condom Mid | Year midpoint of logistic scale-up of condom use in marital relationships | 2,000.76 | (2,000.28, 2,002.48) |
| Mrtl Condom Rate | Rate of logistic scale-up of condom use in marital relationships | 3.49 | (3.20, 3.61) |
| Pr Ex Infrml Fem LOW | Probability of potential for extra-relational informal relationship among low-risk females | 0.28 | (0.26, 0.32) |
| Pr Ex Infrml Fem MED | Probability of potential for extra-relational informal relationship among medium-risk females | 0.34 | (0.24, 0.37) |
| Pr Ex Infrml Male LOW | Probability of potential for extra-relational informal relationship among low-risk males | 0.49 | (0.46, 0.56) |
| Pr Ex Infrml Male MED | Probability of potential for extra-relational informal relationship among medium-risk males | 0.24 | (0.20, 0.28) |
| Pr Ex Trns Fem LOW | Probability of potential for extra-relational transitory relationship among low-risk females | 0.098 | (0.086, 0.118) |
| Pr Ex Trns Fem MED | Probability of potential for extra-relational transitory relationship among medium-risk females | 0.72 | (0.66, 0.78) |
| Pr Ex Trns Male LOW | Probability of potential for extra-relational transitory relationship among low-risk males | 0.2 | (0.14, 0.23) |
| Pr Ex Trns Male MED | Probability of potential for extra-relational transitory relationship among medium-risk males | 0.69 | (0.68, 0.75) |
| Risk Assortivity | Risk assortivity | 0.62 | (0.61, 0.65) |
| SeedYr HIGH | Seed year | 1,976.87 | (1,975.63, 1,977.77) |
| Trans Form Rate | Transitory relationship formation rate | 0.0003 | (0.0001, 0.0004) |
| Trns Condom Mid | Year midpoint of logistic scale-up of condom use in transitory relationships | 2,001.80 | (1,999.58, 2,003.89) |
| Trns Condom Rate | Rate of logistic scale-up of condom use in transitory relationships | 3.23 | (2.88, 3.48) |
| Trns Condoms Max | Maximum rate of condom use in transitory relationships | 0.22 | (0.19, 0.25) |

* Median and interquartile ranges (IQRs) reported for all dynamic parameters used in the calibration process from 100 best-fitting parameter sets. †

###

### Table S8. Utility weights for estimating disability-adjusted life-years averted

| Health State | DALY Weight | Reference |
| --- | --- | --- |
| HIV-negative | 0 | Vos *et al* (4) |
| HIV and not on ART | 0.274 |  |
| HIV and on ART | 0.078 |  |

# References

1. D. J. Klein, "Relationship formation and flow control algorithms for generating age-structured networks in HIV modeling," 2012 IEEE 51st IEEE Conference on Decision and Control (CDC), Maui, HI, USA, 2012, pp. 1041-1046, doi: 10.1109/CDC.2012.6426573

2. Institutes for Disease Modeling. Relationships and contact networks — HIV Model documentation [Internet]. [cited 2023 Jul 23]. Available from: https://docs.idmod.org/projects/emod-hiv/en/latest/sti-model-relationships.html. Accessed 1 Sept. 2023.

3. Bershteyn A, Klein D. STI and HIV Model Introduction [Internet]. 2015 [cited 2023 Jul 23]. Report No.: IAS 2015. Available from: https://institutefordiseasemodeling.github.io/EMOD/STI_and_HIV_Tutorials.pdf. Accessed 1 Sept. 2023.

4. Vos T, Lim SS, Abbafati C, Abbas KM, Abbasi M, Abbasifard M, Abbasi-Kangevari M, Abbastabar H, Abd-Allah F, Abdelalim A, Abdollahi M. Global burden of 369 diseases and injuries in 204 countries and territories, 1990–2019: a systematic analysis for the Global Burden of Disease Study 2019. The Lancet. 2020 Oct 17;396(10258):1204-22.
