# Supplementary material for: Cost thresholds for anticipated long‐acting HIV pre‐exposure prophylaxis products in Eastern and Southern Africa: a mathematical modelling study
Source: J Int AIDS Soc. 2025 Feb 24;28(2):e26427. doi: 10.1002/jia2.26427 (PMC11850439; doi:10.1002/jia2.26427)
Supplement: Supplementary file 2 — Supporting Information [file JIA2-28-e26427-s002.docx]

**Online Supplemental Appendix 2**

**Accompanying the manuscript:**

**Health impact and price threshold for long acting PrEP scale up in Africa: a modeling analysis**

**Contents**

Costs and health outcomes of under different scale up assumptions

Table S1: Additional health outcomes of LA PrEP interventions by country assuming 5% LA PrEP scale up to all adults starting in 2030 2

Table S2: Costs and health outcomes of LA PrEP interventions by country assuming 10% LA PrEP scale up to all adults starting in 2030 4

Table S3: Additional health outcomes of LA PrEP interventions by country assuming 10% LA PrEP scale up to all adults starting in 2030 6

Table S4: Costs and health outcomes of LA PrEP interventions by country assuming 15% LA PrEP scale up to all adults starting in 2030 8

Table S5: Additional health outcomes of LA PrEP interventions by country assuming 15% LA PrEP scale up to all adults starting in 2030 10

Table S6: Costs and health outcomes of LA PrEP interventions by country assuming 20% LA PrEP scale up to all adults starting in 2030 12

Table S7: Additional health outcomes of LA PrEP interventions by country assuming 20% LA PrEP scale up to all adults starting in 2030 14

HIV infections averted and maximum annual price thresholds

Table S8: HIV infections averted and maximum annual price thresholds for LA PrEP assuming PrEP scale up among all adults starting in 2035 16

Table S8a. HIV infections averted at varying LA PrEP coverage levels 16

Table S8b. Maximum price per person year at varying LA PrEP coverage levels 17

Table S8: HIV infections averted and maximum annual price thresholds for LA PrEP assuming PrEP scale up among females only starting in 2035 18

Table S8a. HIV infections averted at varying LA PrEP coverage levels 18

Table S8b. Maximum cost per person year at varying LA PrEP coverage levels 19

# Table S1: Additional health outcomes of LA PrEP interventions by country assuming 5% LA PrEP scale up to all adults starting in 2030

| **Population** | **Product** | **HIV Related Deaths Averted** | **Infections Averted** | **DALYs Averted** | **Person Years of ART Averted** | **HIV Related Deaths** |
| --- | --- | --- | --- | --- | --- | --- |
| **Western Kenya** |  |  |  |  |  |  |
| **Risk-Prioritized** | ISL | 2,837 (2,534, 3,148) | 32,765 (30,670, 34,804) | 50,718 (47,095, 54,290) | 237,936 (223,619, 251,679) | 103,941 (102,784, 105,063) |
|  | CAB | 4,037 (3,642, 4,438) | 47,375 (45,307, 49,484) | 71,198 (62,288, 79,950) | 348,042 (322,827, 373,754) | 102,740 (101,579, 103,888) |
|  | LEN | 4,270 (3,891, 4,675) | 47,403 (45,548, 49,355) | 74,456 (66,608, 82,056) | 352,582 (330,513, 374,855) | 102,507 (101,352, 103,576) |
| **Non-prioritized** | ISL | 974 (638, 1,319) | 10,742 (9,003, 12,465) | 16,264 (12,806, 19,745) | 74,873 (64,168, 86,046) | 105,804 (104,645, 106,937) |
|  | CAB | 1,384 (1,005, 1,760) | 15,957 (14,070, 17,929) | 22,498 (12,879, 31,475) | 118,168 (92,896, 144,182) | 105,393 (104,165, 106,513) |
|  | LEN | 1,347 (916, 1,770) | 15,062 (13,097, 17,023) | 24,056 (16,595, 31,636) | 116,407 (93,861, 139,116) | 105,431 (104,285, 106,513) |
| **Zimbabwe** |  |  |  |  |  |  |
| **Risk-Prioritized** | ISL | 23,756 (17,597, 29,779) | 98,228 (72,811, 122,190) | 222,129 (170,034, 275,228) | 501,058 (392,384, 615,112) | 518,385 (502,943, 534,363) |
|  | CAB | 38,874 (32,840, 45,426) | 159,147 (131,807, 188,526) | 358,194 (293,862, 425,415) | 739,376 (600,015, 892,998) | 503,267 (488,934, 517,659) |
|  | LEN | 41,082 (34,095, 48,469) | 168,354 (141,776, 197,296) | 378,494 (309,085, 451,597) | 850,678 (708,208, 1,001,361) | 501,059 (486,471, 516,382) |
| **Non-prioritized** | ISL | 10,651 (4,962, 16,480) | 33,868 (9,043, 57,645) | 95,145 (44,280, 144,506) | 171,121 (58,631, 283,553) | 531,490 (515,488, 547,418) |
|  | CAB | 14,618 (8,244, 21,115) | 59,193 (32,943, 85,704) | 147,358 (81,291, 213,617) | 293,118 (159,037, 429,148) | 527,523 (512,761, 542,971) |
|  | LEN | 16,541 (9,757, 23,033) | 54,180 (27,876, 80,966) | 143,821 (75,378, 212,032) | 299,639 (163,197, 440,111) | 525,600 (509,522, 541,234) |
| **South Africa** |  |  |  |  |  |  |
| **Risk-Prioritized** | ISL | 275,151 (255,005, 295,363) | 1,035,747 (969,260, 1,102,971) | 2,391,588 (2,223,928, 2,560,443) | 7,067,922 (6,654,584, 7,476,306) | 5,367,932 (5,307,140, 5,428,220) |
|  | CAB | 501,460 (474,135, 529,690) | 1,906,114 (1,828,709, 1,979,348) | 4,423,109 (4,094,670, 4,768,968) | 12,904,148 (12,339,745, 13,482,037) | 5,141,622 (5,079,796, 5,206,477) |
|  | LEN | 498,372(467,543, 530,209) | 1,925,853(1,849,413, 2,002,691) | 4,396,483(4,049,327, 4,748,352) | 12,901,605(12,299,277, 13,521,511) | 5,144,710(5,083,084, 5,206,637) |
| **Non-prioritized** | ISL | 51,524 (33,948, 70,074) | 237,501 (172,417, 300,747) | 536,288 (391,596, 676,132) | 1,533,284 (1,176,189, 1,909,395) | 5,591,558 (5,529,352, 5,653,130) |
|  | CAB | 118,525 (90,757, 145,028) | 477,760 (404,887, 552,350) | 1,209,648 (875,818, 1,534,887) | 3,412,939 (2,843,281, 3,962,443) | 5,524,558 (5,461,454, 5,591,312) |
|  | LEN | 96,244 (65,780, 128,162) | 384,108 (313,468, 455,502) | 1,022,164 (663,383, 1,392,101) | 2,770,167 (2,216,583, 3,345,410) | 5,546,838 (5,486,906, 5,610,245) |

# Table S2: Costs and health outcomes of LA PrEP interventions by country assuming 10% LA PrEP scale up to all adults starting in 2030

| **Population** | **Product** | **% of infections averted** | **% of deaths averted** | | **Person Years on PrEP** | **Person Years on LA PrEP Per DALY Averted** | **Max Cost per Person Year on LA PrEP** | **Max Cost per Dose** |
| --- | --- | --- | --- | --- | --- | --- | --- | --- |
| **Western Kenya** |  |  |  | |  |  |  |  |
| **Risk-Prioritized** | ISL | 20.55 (19.91, 21.19) | 4.48 (4.2, 4.73) | 6,445,815 (6,440,011, 6,452,035) | | 75 (72, 79) | $9.2 ($8.81, $9.59) | $0.77 ($0.73, $0.8) |
|  | CAB | 29.33 (28.72, 29.92) | 6.18 (5.8, 6.55) | 6,328,915 (6,322,672, 6,335,325) | | 53 (50, 58) | $12.93 ($11.98, $13.86) | $2.16 ($2.0, $2.31) |
|  | LEN | 28.96 (28.32, 29.6) | 6.21 (5.84, 6.58) | 6,591,706 (6,586,215, 6,597,150) | | 55 (51, 59) | $12.64 ($11.8, $13.48) | $6.32 ($5.9, $6.74) |
| **Non-prioritized** | ISL | 8.43 (7.71, 9.17) | 1.88 (1.55, 2.2) | 6,632,935 (6,626,630, 6,639,515) | | 194 (177, 215) | $3.57 ($3.24, $3.93) | $0.3 ($0.27, $0.33) |
|  | CAB | 12.33 (11.54, 13.08) | 2.72 (2.34, 3.12) | 6,612,773 (6,606,294, 6,619,780) | | 137 (116, 169) | $5.07 ($4.13, $6.01) | $0.85 ($0.69, $1.0) |
|  | LEN | 12.44 (11.74, 13.13) | 2.86 (2.43, 3.28) | 6,635,414 (6,629,191, 6,641,967) | | 130 (111, 156) | $5.37 ($4.46, $6.24) | $2.68 ($2.23, $3.12) |
| **Zimbabwe** |  |  |  |  | |  |  |  |
| **Risk-Prioritized** | ISL | 22.97 (20.35, 25.44) | 8.48 (7.51, 9.47) | 15,141,921 (15,101,821, 15,179,450) | | 36 (32, 41) | $16.34 ($14.43, $18.38) | $1.36 ($1.2, $1.53) |
|  | CAB | 33.87 (31.61, 36.19) | 12.95 (11.85, 14.03) | 15,026,895 (14,989,774, 15,062,808) | | 23 (21, 26) | $25.1 ($22.24, $27.99) | $4.18 ($3.71, $4.66) |
|  | LEN | 33.48 (31.1, 35.75) | 12.8 (11.71, 13.83) | 15,310,203 (15,272,778, 15,347,096) | | 24 (21, 27) | $24.35 ($21.65, $27.12) | $12.17 ($10.83, $13.56) |
| **Non-prioritized** | ISL | 7.66 (4.49, 10.81) | 3.65 (2.6, 4.68) | 15,001,641 (14,954,341, 15,049,053) | | 88 (67, 128) | $6.7 ($4.61, $8.87) | $0.56 ($0.38, $0.74) |
|  | CAB | 13.1 (9.7, 16.66) | 5.08 (3.81, 6.35) | 14,975,373 (14,928,781, 15,022,318) | | 60 (46, 85) | $9.78 ($6.91, $12.73) | $1.63 ($1.15, $2.12) |
|  | LEN | 14.05 (11.08, 16.87) | 5.48 (4.3, 6.67) | 15,018,796 (14,973,342, 15,065,276) | | 54 (43, 72) | $10.87 ($8.16, $13.74) | $5.44 ($4.08, $6.87) |
| **South Africa** |  |  |  |  | |  |  |  |
| **Risk-Prioritized** | ISL | 16.83 (16.1, 17.61) | 6.3 (5.95, 6.66) | 50,791,134 (50,672,278, 50,905,650) | | 15 (15, 16) | $43.07 ($40.69, $45.46) | $3.59 ($3.39, $3.79) |
|  | CAB | 27.35(26.55, 28.14) | 10.76(10.26, 11.25) | 51,005,659(50,884,149, 51,119,658) | | 9 (8, 10) | $72.15 ($67.47, $76.77) | $12.02 ($11.24, $12.8) |
|  | LEN | 27.41 (26.74, 28.06) | 10.51 (10.05, 10.99) | 51,431,792 (51,313,671, 51,543,625) | | 9 (9, 10) | $70.26 ($65.87, $74.66) | $35.13 ($32.93, $37.33) |
| **Non-prioritized** | ISL | 5.11 (4.28, 5.91) | 1.69 (1.32, 2.05) | 51,437,988 (51,301,030, 51,573,196) | | 57 (48, 70) | $11.99 ($9.76, $14.15) | $1.0 ($0.81, $1.18) |
|  | CAB | 9.73 (8.94, 10.49) | 3.75 (3.25, 4.26) | 51,567,468 (51,424,262, 51,711,353) | | 25 (21, 30) | $27.24 ($22.78, $31.65) | $4.54 ($3.8, $5.27) |
|  | LEN | 8.86 (8.06, 9.66) | 3.3 (2.76, 3.82) | 51,538,443 (51,396,053, 51,671,603) | | 29 (24, 36) | $23.52 ($18.84, $28.32) | $11.76 ($9.42, $14.16) |

# Table S3: Additional health outcomes of LA PrEP interventions by country assuming 10% LA PrEP scale up to all adults starting in 2030

| **Population** | **Product** | **HIV Related Deaths Averted** | **Infections Averted** | **DALYs Averted** | **Person Years of ART Averted** | **HIV Related Deaths** |
| --- | --- | --- | --- | --- | --- | --- |
| **Western Kenya** |  |  |  |  |  |  |
| **Risk-Prioritized** | ISL | 4,775 (4,479, 5,078) | 51,996 (49,913, 54,104) | 85,045 (81,597, 88,534) | 385,179 (371,221, 398,964) | 102,002 (100,912, 103,063) |
|  | CAB | 6,594 (6,195, 6,993) | 74,225 (71,893, 76,465) | 117,410 (108,651, 126,192) | 558,551 (532,990, 584,578) | 100,184 (99,094, 101,259) |
|  | LEN | 6,623 (6,215, 7,026) | 73,272 (70,948, 75,616) | 119,456 (111,552, 127,529) | 556,246 (531,889, 580,516) | 100,155 (99,074, 101,218) |
| **Non-prioritized** | ISL | 2,006 (1,659, 2,340) | 21,304 (19,360, 23,198) | 34,026 (30,640, 37,200) | 154,729 (142,056, 167,411) | 104,771 (103,568, 105,912) |
|  | CAB | 2,910 (2,476, 3,312) | 31,204 (29,081, 33,313) | 48,136 (39,254, 57,040) | 230,822 (204,102, 256,018) | 103,868 (102,721, 105,040) |
|  | LEN | 3,057 (2,578, 3,522) | 31,459 (29,469, 33,454) | 50,952 (42,695, 59,550) | 234,028 (211,206, 256,539) | 103,720 (102,611, 104,784) |
| **Zimbabwe** |  |  |  |  |  |  |
| **Risk-Prioritized** | ISL | 45,924 (40,258, 51,591) | 183,929 (158,992, 209,666) | 417,100 (370,160, 473,582) | 859,742 (758,002, 965,924) | 496,216 (481,211, 511,080) |
|  | CAB | 70,263 (63,302, 77,627) | 271,474 (244,702, 301,107) | 634,328 (562,800, 706,829) | 1,338,550 (1,196,556, 1,484,955) | 471,878 (458,867, 484,954) |
|  | LEN | 69,353 (62,627, 75,914) | 268,411 (241,205, 297,616) | 627,364 (557,407, 700,497) | 1,353,985 (1,213,857, 1,500,219) | 472,788 (459,820, 486,161) |
| **Non-prioritized** | ISL | 19,785 (13,722, 25,705) | 61,889 (36,293, 89,780) | 169,304 (117,249, 224,630) | 324,537 (205,410, 441,777) | 522,356 (506,498, 537,685) |
|  | CAB | 27,491 (20,154, 34,978) | 105,407 (76,329, 137,348) | 246,088 (173,977, 320,153) | 502,512 (353,389, 655,025) | 514,650 (499,462, 529,500) |
|  | LEN | 29,685 (22,979, 36,483) | 112,694 (87,398, 139,143) | 275,156 (203,258, 347,443) | 591,941 (449,724, 745,052) | 512,456 (498,070, 527,272) |
| **South Africa** |  |  |  |  |  |  |
| **Risk-Prioritized** | ISL | 355,254 (334,338, 376,149) | 1,468,887 (1,405,216, 1,532,317) | 3,183,801 (3,012,520, 3,360,912) | 9,613,966 (9,213,749, 10,021,599) | 5,287,828 (5,229,048, 5,349,956) |
|  | CAB | 606,829(577,438, 636,294) | 2,386,078(2,310,224, 2,461,540) | 5,351,623(5,004,096, 5,690,385) | 15,860,555(15,284,507, 16,415,051) | 5,036,254(4,976,560, 5,097,163) |
|  | LEN | 593,404 (566,216, 620,724) | 2,391,239 (2,322,909, 2,456,795) | 5,260,775 (4,924,919, 5,598,972) | 15,888,246 (15,331,409, 16,472,427) | 5,049,679 (4,987,522, 5,114,523) |
| **Non-prioritized** | ISL | 95,816 (74,476, 116,533) | 445,888 (373,830, 516,742) | 896,334 (733,983, 1,059,590) | 3,040,610 (2,618,935, 3,458,624) | 5,547,266 (5,483,631, 5,609,096) |
|  | CAB | 211,671 (182,594, 239,043) | 848,954 (778,971, 917,111) | 2,041,136 (1,698,672, 2,368,363) | 5,810,225 (5,252,984, 6,361,325) | 5,431,412 (5,364,464, 5,501,248) |
|  | LEN | 185,722 (154,618, 218,072) | 773,302 (698,567, 847,370) | 1,765,898 (1,413,649, 2,132,214) | 5,400,424 (4,829,978, 5,961,691) | 5,457,361 (5,395,748, 5,519,464) |

# Table S4: Costs and health outcomes of LA PrEP interventions by country assuming 15% LA PrEP scale up to all adults starting in 2030

| **Population** | **Product** | **% of infections averted** | **% of deaths averted** | **Person Years on PrEP** | **Person Years on LA PrEP Per DALY Averted** | **Max Cost per Person Year on LA PrEP** | **Max Cost per Dose** |
| --- | --- | --- | --- | --- | --- | --- | --- |
| **Western Kenya** |  |  |  |  |  |  |  |
| **Risk-Prioritized** | ISL | 25.16 (24.61, 25.7) | 5.27 (4.94, 5.6) | 8,960,059 (8,945,727, 8,974,453) | 86 (83, 89) | $8.06 ($7.75, $8.38) | $0.67 ($0.65, $0.7) |
|  | CAB | 36.25 (35.69, 36.8) | 7.9 (7.56, 8.25) | 8,783,184 (8,768,993, 8,798,024) | 59 (55, 62) | $11.77 ($11.1, $12.45) | $1.96 ($1.85, $2.07) |
|  | LEN | 35.39 (34.86, 35.92) | 7.54 (7.15, 7.92) | 9,359,347 (9,347,152, 9,371,478) | 64 (60, 67) | $10.86 ($10.24, $11.47) | $5.43 ($5.12, $5.73) |
| **Non-prioritized** | ISL | 12.6 (11.93, 13.27) | 2.88 (2.57, 3.2) | 9,954,772 (9,945,466, 9,964,692) | 195 (181, 211) | $3.57 ($3.3, $3.84) | $0.3 ($0.27, $0.32) |
|  | CAB | 18.57 (17.8, 19.3) | 4.2 (3.78, 4.62) | 9,925,891 (9,916,058, 9,935,946) | 138 (123, 157) | $5.05 ($4.41, $5.67) | $0.84 ($0.74, $0.94) |
|  | LEN | 18.59 (17.95, 19.2) | 4.28 (3.9, 4.66) | 9,961,784 (9,952,741, 9,971,318) | 132 (120, 147) | $5.24 ($4.71, $5.8) | $2.62 ($2.36, $2.9) |
| **Zimbabwe** |  |  |  |  |  |  |  |
| **Risk-Prioritized** | ISL | 25.55 (23.37, 27.61) | 9.56 (8.69, 10.43) | 22,136,989 (22,070,448, 22,204,028) | 46 (42, 51) | $12.78 ($11.47, $14.19) | $1.06 ($0.96, $1.18) |
|  | CAB | 37.72 (35.64, 39.86) | 14.58 (13.62, 15.55) | 21,973,134 (21,909,913, 22,039,201) | 30 (27, 33) | $19.34 ($17.57, $21.32) | $3.22 ($2.93, $3.55) |
|  | LEN | 36.6 (34.29, 38.9) | 14.41 (13.35, 15.42) | 22,450,877 (22,385,314, 22,515,567) | 31 (28, 34) | $18.9 ($17.03, $20.81) | $9.45 ($8.52, $10.41) |
| **Non-prioritized** | ISL | 10.21 (7.14, 12.96) | 3.79 (2.74, 4.83) | 22,508,629 (22,437,248, 22,579,512) | 116 (92, 162) | $5.07 ($3.67, $6.44) | $0.42 ($0.31, $0.54) |
|  | CAB | 18.75 (16.2, 21.3) | 6.96 (5.86, 8.03) | 22,479,665 (22,413,603, 22,546,436) | 64 (54, 78) | $9.23 ($7.58, $10.97) | $1.54 ($1.26, $1.83) |
|  | LEN | 18.76 (15.73, 21.7) | 7.19 (6.08, 8.25) | 22,549,347 (22,482,223, 22,616,869) | 61 (51, 75) | $9.67 ($7.87, $11.48) | $4.84 ($3.94, $5.74) |
| **South Africa** |  |  |  |  |  |  |  |
| **Risk-Prioritized** | ISL | 20.14 (19.41, 20.87) | 7.42 (7.11, 7.73) | 74,914,194 (74,731,852, 75,090,802) | 19 (19, 20) | $34.68 ($33.23, $36.17) | $2.89 ($2.77, $3.01) |
|  | CAB | 32.21 (31.58, 32.86) | 12.15 (11.69, 12.63) | 75,242,349 (75,061,956, 75,415,678) | 12 (11, 12) | $56.09 ($53.05, $59.23) | $9.35 ($8.84, $9.87) |
|  | LEN | 32.3 (31.53, 33.06) | 11.89 (11.41, 12.37) | 76,190,839 (76,015,849, 76,360,520) | 12 (11, 13) | $54.15 ($51.09, $57.32) | $27.07 ($25.54, $28.66) |
| **Non-prioritized** | ISL | 8.23 (7.57, 8.9) | 2.9 (2.58, 3.22) | 77,281,836 (77,078,770, 77,482,081) | 51 (46, 56) | $13.42 ($12.13, $14.7) | $1.12 ($1.01, $1.22) |
|  | CAB | 13.95 (13.16, 14.72) | 5.32 (4.83, 5.8) | 77,523,589 (77,311,096, 77,726,068) | 27 (24, 31) | $25.13 ($22.16, $28.11) | $4.19 ($3.69, $4.69) |
|  | LEN | 13.97 (13.21, 14.72) | 5.24 (4.73, 5.76) | 77,538,163 (77,326,174, 77,743,484) | 27 (24, 31) | $24.66 ($21.52, $27.82) | $12.33 ($10.76, $13.91) |

# Table S5: Additional health outcomes of LA PrEP interventions by country assuming 15% LA PrEP scale up to all adults starting in 2030

| **Population** | **Product** | **HIV Related Deaths Averted** | | **Infections Averted** | **DALYs Averted** | **Person Years of ART Averted** | **HIV Related Deaths** |
| --- | --- | --- | --- | --- | --- | --- | --- |
| **Western Kenya** |  |  | |  |  |  |  |
| **Risk-Prioritized** | ISL | 5,635 (5,286, 5,980) | 63,639 (61,683, 65,669) | | 103,619 (99,703, 107,539) | 471,871 (458,365, 485,410) | 101,142 (100,051, 102,202) |
|  | CAB | 8,434 (8,037, 8,818) | 91,706 (89,341, 94,035) | | 148,278 (139,645, 157,133) | 696,520 (671,579, 721,996) | 98,343 (97,244, 99,403) |
|  | LEN | 8,052 (7,607, 8,486) | 89,546 (87,251, 91,830) | | 145,849 (137,338, 154,000) | 687,736 (665,225, 710,758) | 98,725 (97,636, 99,777) |
| **Non-prioritized** | ISL | 3,079 (2,737, 3,420) | 31,874 (29,978, 33,749) | | 50,945 (47,143, 54,816) | 229,801 (217,652, 242,454) | 103,699 (102,572, 104,746) |
|  | CAB | 4,485 (4,022, 4,939) | 46,992 (44,857, 49,121) | | 71,854 (62,605, 80,961) | 339,385 (314,055, 363,987) | 102,293 (101,167, 103,382) |
|  | LEN | 4,572 (4,155, 4,978) | 47,034 (45,120, 48,973) | | 74,990 (67,090, 82,689) | 343,658 (319,989, 365,407) | 102,205 (101,066, 103,262) |
| **Zimbabwe** |  |  |  | |  |  |  |
| **Risk-Prioritized** | ISL | 51,871 (46,400, 57,291) | 204,745 (181,435, 227,797) | | 475,744 (427,984, 528,041) | 1,048,746 (940,702, 1,154,542) | 490,270 (475,918, 504,996) |
|  | CAB | 79,123 (72,816, 86,000) | 302,226 (273,923, 332,599) | | 715,341 (648,418, 785,513) | 1,502,304 (1,361,123, 1,656,860) | 463,018 (450,419, 475,671) |
|  | LEN | 78,108 (71,535, 84,955) | 293,353 (265,205, 324,807) | | 713,999 (642,878, 787,616) | 1,525,773 (1,377,866, 1,687,294) | 464,033 (450,767, 477,301) |
| **Non-prioritized** | ISL | 20,497 (14,542, 26,323) | 82,038 (56,437, 105,687) | | 192,546 (140,329, 243,199) | 418,293 (295,414, 534,302) | 521,644 (506,703, 536,948) |
|  | CAB | 37,755 (31,692, 44,194) | 150,291 (124,478, 176,368) | | 349,040 (286,259, 415,359) | 696,840 (561,600, 842,855) | 504,386 (489,977, 518,287) |
|  | LEN | 38,994 (32,912, 45,375) | 150,252 (122,693, 179,310) | | 366,572 (299,094, 435,043) | 774,512 (626,781, 920,955) | 503,147 (488,298, 517,719) |
| **South Africa** |  |  |  | |  |  |  |
| **Risk-Prioritized** | ISL | 418,727 (399,676, 436,878) | 1,757,733 (1,692,341, 1,823,379) | | 3,780,189 (3,622,163, 3,942,881) | 11,684,143 (11,313,674, 12,059,043) | 5,224,355 (5,164,790, 5,286,372) |
|  | CAB | 685,970 (658,400, 713,558) | 2,810,434 (2,747,824, 2,875,421) | | 6,144,664 (5,800,192, 6,475,646) | 18,542,461 (18,026,709, 19,050,470) | 4,957,113 (4,896,372, 5,023,289) |
|  | LEN | 671,083 (641,978, 700,019) | 2,818,485 (2,740,078, 2,896,729) | | 6,007,947 (5,663,002, 6,348,607) | 18,394,239 (17,796,701, 19,002,820) | 4,971,999 (4,913,598, 5,030,146) |
| **Non-prioritized** | ISL | 163,878 (144,790, 182,245) | 717,846 (656,300, 777,009) | | 1,510,336 (1,362,694, 1,656,486) | 4,710,797 (4,334,372, 5,073,322) | 5,479,204 (5,418,941, 5,539,868) |
|  | CAB | 300,157 (272,757, 327,267) | 1,218,106 (1,146,551, 1,286,687) | | 2,834,709 (2,490,558, 3,169,683) | 8,147,324 (7,599,668, 8,692,564) | 5,342,926 (5,281,340, 5,408,050) |
|  | LEN | 295,350 (264,822, 325,573) | 1,218,477 (1,147,374, 1,290,845) | | 2,783,070 (2,433,824, 3,127,290) | 8,050,340 (7,456,802, 8,629,649) | 5,347,733 (5,285,986, 5,412,842) |

# Table S6: Costs and health outcomes of LA PrEP interventions by country assuming 20% LA PrEP scale up to all adults starting in 2030

| **Population** | **product** | **% of infections averted** | **% of deaths averted** | | **Person Years on PrEP** | **Person Years on LA PrEP Per DALY Averted** | | **Max Cost per Person Year on LA PrEP** | **Max Cost per Dose** |
| --- | --- | --- | --- | --- | --- | --- | --- | --- | --- |
| **Western Kenya** |  |  |  | |  |  | |  |  |
| **Risk-Prioritized** | ISL | 28.55 (27.94, 29.14) | 6.17 (5.88, 6.46) | 10,770,779 (10,743,150, 10,798,708) | | | 91 (88, 94) | $7.62 ($7.37, $7.88) | $0.64 ($0.61, $0.66) |
|  | CAB | 40.96 (40.45, 41.47) | 8.97 (8.6, 9.34) | 10,528,317 (10,501,997, 10,555,472) | | | 61 (58, 65) | $11.27 ($10.65, $11.89) | $1.88 ($1.78, $1.98) |
|  | LEN | 40.6 (40.11, 41.09) | 8.89 (8.55, 9.24) | 11,441,859 (11,418,331, 11,466,156) | | | 66 (63, 70) | $10.42 ($9.94, $10.91) | $5.21 ($4.97, $5.45) |
| **Non-prioritized** | ISL | 16.19 (15.6, 16.78) | 3.7 (3.36, 4.03) | 13,279,053 (13,266,491, 13,292,556) | | | 197 (187, 208) | $3.53 ($3.34, $3.72) | $0.29 ($0.28, $0.31) |
|  | CAB | 23.24 (22.6, 23.87) | 5.17 (4.8, 5.53) | 13,243,208 (13,230,199, 13,256,599) | | | 143 (130, 159) | $4.85 ($4.38, $5.33) | $0.81 ($0.73, $0.89) |
|  | LEN | 24.13 (23.5, 24.73) | 5.31 (4.96, 5.67) | 13,292,773 (13,281,286, 13,304,823) | | | 136 (125, 148) | $5.12 ($4.72, $5.52) | $2.56 ($2.36, $2.76) |
| **Zimbabwe** |  |  |  |  | | |  |  |  |
| **Risk-Prioritized** | ISL | 27.67 (25.55, 29.85) | 10.16 (9.35, 10.98) | 28,750,950 (28,636,538, 28,863,462) | | | 55 (50, 61) | $10.68 ($9.68, $11.72) | $0.89 ($0.81, $0.98) |
|  | CAB | 40.71 (38.52, 42.9) | 15.49 (14.46, 16.5) | 28,555,495 (28,446,019, 28,663,007) | | | 36 (33, 40) | $16.19 ($14.66, $17.8) | $2.7 ($2.44, $2.97) |
|  | LEN | 40.5 (38.67, 42.25) | 15.72 (14.82, 16.6) | 29,331,357 (29,226,681, 29,435,379) | | | 37 (34, 40) | $15.87 ($14.5, $17.36) | $7.93 ($7.25, $8.68) |
| **Non-prioritized** | ISL | 16.05 (13.54, 18.41) | 6.41 (5.47, 7.34) | 30,031,410 (29,936,653, 30,122,485) | | | 98 (85, 115) | $6.04 ($5.14, $6.94) | $0.5 ($0.43, $0.58) |
|  | CAB | 21.95 (18.91, 24.85) | 8.83 (7.62, 10.0) | 29,991,216 (29,896,233, 30,080,222) | | | 69 (60, 82) | $8.51 ($7.2, $9.9) | $1.42 ($1.2, $1.65) |
|  | LEN | 23.02 (20.45, 25.55) | 9.06 (7.97, 10.14) | 30,084,217 (29,996,334, 30,170,227) | | | 66 (57, 78) | $8.92 ($7.63, $10.25) | $4.46 ($3.81, $5.13) |
| **South Africa** |  |  |  |  | | |  |  |  |
| **Risk-Prioritized** | ISL | 22.49 (21.78, 23.2) | 8.35 (8.01, 8.71) | 97,965,670 (97,724,105, 98,198,695) | | | 23 (22, 24) | $29.72 ($28.49, $30.95) | $2.48 ($2.37, $2.58) |
|  | CAB | 35.22 (34.48, 35.93) | 13.23 (12.78, 13.7) | 98,361,730 (98,126,402, 98,601,311) | | | 14 (13, 15) | $47.31 ($44.95, $49.67) | $7.88 ($7.49, $8.28) |
|  | LEN | 35.32 (34.63, 36.03) | 13.21 (12.72, 13.7) | 100,169,474 (99,931,584, 100,393,501) | | | 14 (14, 15) | $45.91 ($43.58, $48.29) | $22.96 ($21.79, $24.15) |
| **Non-prioritized** | ISL | 10.36 (9.74, 10.98) | 3.72 (3.39, 4.06) | 103,184,253 (102,922,844, 103,437,375) | | | 53 (49, 58) | $12.87 ($11.87, $13.9) | $1.07 ($0.99, $1.16) |
|  | CAB | 18.22 (17.39, 19.02) | 6.94 (6.4, 7.47) | 103,678,871 (103,394,410, 103,952,465) | | | 28 (25, 31) | $24.14 ($21.82, $26.45) | $4.02 ($3.64, $4.41) |
|  | LEN | 18.04 (17.36, 18.73) | 6.8 (6.3, 7.3) | 103,660,582 (103,396,155, 103,920,511) | | | 29 (26, 32) | $23.4 ($21.15, $25.68) | $11.7 ($10.58, $12.84) |

# Table S7: Additional health outcomes of LA PrEP interventions by country assuming 20% LA PrEP scale up to all adults starting in 2030

| **Population** | **product** | **% of infections averted** | **% of deaths averted** | | **Person Years on PrEP** | **Person Years on LA PrEP Per DALY Averted** | | **Max Cost per Person Year on LA PrEP** | **Max Cost per Dose** |
| --- | --- | --- | --- | --- | --- | --- | --- | --- | --- |
| **Western Kenya** |  |  |  | |  |  | |  |  |
| **Risk-Prioritized** | ISL | 28.55 (27.94, 29.14) | 6.17 (5.88, 6.46) | 10,770,779 (10,743,150, 10,798,708) | | | 91 (88, 94) | $7.62 ($7.37, $7.88) | $0.64 ($0.61, $0.66) |
|  | CAB | 40.96 (40.45, 41.47) | 8.97 (8.6, 9.34) | 10,528,317 (10,501,997, 10,555,472) | | | 61 (58, 65) | $11.27 ($10.65, $11.89) | $1.88 ($1.78, $1.98) |
|  | LEN | 40.6 (40.11, 41.09) | 8.89 (8.55, 9.24) | 11,441,859 (11,418,331, 11,466,156) | | | 66 (63, 70) | $10.42 ($9.94, $10.91) | $5.21 ($4.97, $5.45) |
| **Non-prioritized** | ISL | 16.19 (15.6, 16.78) | 3.7 (3.36, 4.03) | 13,279,053 (13,266,491, 13,292,556) | | | 197 (187, 208) | $3.53 ($3.34, $3.72) | $0.29 ($0.28, $0.31) |
|  | CAB | 23.24 (22.6, 23.87) | 5.17 (4.8, 5.53) | 13,243,208 (13,230,199, 13,256,599) | | | 143 (130, 159) | $4.85 ($4.38, $5.33) | $0.81 ($0.73, $0.89) |
|  | LEN | 24.13 (23.5, 24.73) | 5.31 (4.96, 5.67) | 13,292,773 (13,281,286, 13,304,823) | | | 136 (125, 148) | $5.12 ($4.72, $5.52) | $2.56 ($2.36, $2.76) |
| **Zimbabwe** |  |  |  |  | | |  |  |  |
| **Risk-Prioritized** | ISL | 27.67 (25.55, 29.85) | 10.16 (9.35, 10.98) | 28,750,950 (28,636,538, 28,863,462) | | | 55 (50, 61) | $10.68 ($9.68, $11.72) | $0.89 ($0.81, $0.98) |
|  | CAB | 40.71 (38.52, 42.9) | 15.49 (14.46, 16.5) | 28,555,495 (28,446,019, 28,663,007) | | | 36 (33, 40) | $16.19 ($14.66, $17.8) | $2.7 ($2.44, $2.97) |
|  | LEN | 40.5 (38.67, 42.25) | 15.72 (14.82, 16.6) | 29,331,357 (29,226,681, 29,435,379) | | | 37 (34, 40) | $15.87 ($14.5, $17.36) | $7.93 ($7.25, $8.68) |
| **Non-prioritized** | ISL | 16.05 (13.54, 18.41) | 6.41 (5.47, 7.34) | 30,031,410 (29,936,653, 30,122,485) | | | 98 (85, 115) | $6.04 ($5.14, $6.94) | $0.5 ($0.43, $0.58) |
|  | CAB | 21.95 (18.91, 24.85) | 8.83 (7.62, 10.0) | 29,991,216 (29,896,233, 30,080,222) | | | 69 (60, 82) | $8.51 ($7.2, $9.9) | $1.42 ($1.2, $1.65) |
|  | LEN | 23.02 (20.45, 25.55) | 9.06 (7.97, 10.14) | 30,084,217 (29,996,334, 30,170,227) | | | 66 (57, 78) | $8.92 ($7.63, $10.25) | $4.46 ($3.81, $5.13) |
| **South Africa** |  |  |  |  | | |  |  |  |
| **Risk-Prioritized** | ISL | 22.49 (21.78, 23.2) | 8.35 (8.01, 8.71) | 97,965,670 (97,724,105, 98,198,695) | | | 23 (22, 24) | $29.72 ($28.49, $30.95) | $2.48 ($2.37, $2.58) |
|  | CAB | 35.22 (34.48, 35.93) | 13.23 (12.78, 13.7) | 98,361,730 (98,126,402, 98,601,311) | | | 14 (13, 15) | $47.31 ($44.95, $49.67) | $7.88 ($7.49, $8.28) |
|  | LEN | 35.32 (34.63, 36.03) | 13.21 (12.72, 13.7) | 100,169,474 (99,931,584, 100,393,501) | | | 14 (14, 15) | $45.91 ($43.58, $48.29) | $22.96 ($21.79, $24.15) |
| **Non-prioritized** | ISL | 10.36 (9.74, 10.98) | 3.72 (3.39, 4.06) | 103,184,253 (102,922,844, 103,437,375) | | | 53 (49, 58) | $12.87 ($11.87, $13.9) | $1.07 ($0.99, $1.16) |
|  | CAB | 18.22 (17.39, 19.02) | 6.94 (6.4, 7.47) | 103,678,871 (103,394,410, 103,952,465) | | | 28 (25, 31) | $24.14 ($21.82, $26.45) | $4.02 ($3.64, $4.41) |
|  | LEN | 18.04 (17.36, 18.73) | 6.8 (6.3, 7.3) | 103,660,582 (103,396,155, 103,920,511) | | | 29 (26, 32) | $23.4 ($21.15, $25.68) | $11.7 ($10.58, $12.84) |

| Table S8a. HIV infections averted at varying LA PrEP coverage levels |
| --- |

# Table S8: HIV infections averted and maximum annual price thresholds for LA PrEP assuming PrEP scale up among all adults starting in 2035

| **Population** | **Product** | **5%** | **10%** | **15%** | **20%** |
| --- | --- | --- | --- | --- | --- |
| **Western Kenya** |  |  |  |  |  |
| **Risk-Prioritized** | ISL | 10.64 (9.89, 11.36) | 16.94 (16.35, 17.51) | 21.06 (20.44, 21.69) | 24.14 (23.56, 24.74) |
|  | CAB | 15.74 (15.0, 16.47) | 24.61 (23.95, 25.26) | 30.72 (30.08, 31.36) | 35.23 (34.71, 35.74) |
|  | LEN | 15.57 (14.96, 16.19) | 24.2 (23.59, 24.8) | 29.89 (29.39, 30.4) | 34.84 (34.32, 35.35) |
| **Non-prioritized** | ISL | 3.42 (2.65, 4.18) | 6.96 (6.24, 7.66) | 10.46 (9.76, 11.14) | 13.61 (12.97, 14.25) |
|  | CAB | 5.21 (4.42, 5.96) | 10.39 (9.63, 11.13) | 14.91 (14.21, 15.62) | 20.2 (19.47, 20.93) |
|  | LEN | 5.25 (4.47, 6.02) | 10.17 (9.48, 10.88) | 15.28 (14.64, 15.9) | 20.04 (19.32, 20.75) |
| **Zimbabwe** |  |  |  |  |  |
| **Risk-Prioritized** | ISL | 10.98 (8.43, 13.43) | 20.18 (17.54, 22.76) | 20.62 (18.06, 23.11) | 23.11 (20.56, 25.65) |
|  | CAB | 19.26 (16.27, 22.29) | 28.78 (26.43, 31.16) | 31.74 (29.38, 34.14) | 34.54 (32.29, 36.75) |
|  | LEN | 17.96 (15.11, 20.65) | 28.88 (26.66, 31.11) | 30.49 (28.15, 32.8) | 34.5 (32.33, 36.63) |
| **Non-prioritized** | ISL | 5.01 (2.33, 7.62) | 6.82 (3.69, 9.83) | 8.58 (5.63, 11.43) | 12.72 (10.18, 15.25) |
|  | CAB | 7.82 (4.44, 11.04) | 11.5 (8.16, 14.85) | 15.5 (12.28, 18.75) | 19.27 (16.51, 21.89) |
|  | LEN | 6.4 (3.21, 9.58) | 10.55 (7.31, 13.61) | 14.65 (11.65, 17.51) | 20.39 (17.68, 23.04) |
| **South Africa** |  |  |  |  |  |
| **Risk-Prioritized** | ISL | 9.74 (9.02, 10.44) | 14.02 (13.32, 14.73) | 16.88 (16.16, 17.61) | 19.39 (18.69, 20.09) |
|  | CAB | 18.84 (18.07, 19.59) | 23.62 (22.83, 24.39) | 27.54 (26.8, 28.28) | 30.36 (29.6, 31.13) |
|  | LEN | 18.17 (17.35, 18.99) | 23.23 (22.49, 23.96) | 27.17 (26.42, 27.87) | 30.19 (29.46, 30.91) |
| **Non-prioritized** | ISL | 2.14 (1.4, 2.89) | 4.56 (3.77, 5.33) | 7.11 (6.33, 7.86) | 9.45 (8.66, 10.19) |
|  | CAB | 4.34 (3.62, 5.03) | 8.76 (7.99, 9.53) | 12.34 (11.64, 13.06) | 15.4 (14.69, 16.12) |
|  | LEN | 4.28 (3.57, 4.99) | 8.15 (7.37, 8.93) | 11.97 (11.2, 12.75) | 15.1 (14.27, 15.96) |

| Table S8b. Maximum price per person year at varying LA PrEP coverage levels | | | | | |
| --- | --- | --- | --- | --- | --- |
| **Population** | **Product** | **5%** | **10%** | **15%** | **20%** |
| **Western Kenya** |  |  |  |  |  |
| **Risk-Prioritized** | ISL | $8.96 ($8.06, $9.89) | $8.14 ($7.7, $8.6) | $7.35 ($7.01, $7.7) | $6.88 ($6.63, $7.14) |
|  | CAB | $13.28 ($11.13, $15.34) | $11.67 ($10.51, $12.8) | $10.72 ($9.88, $11.51) | $10.39 ($9.71, $11.08) |
|  | LEN | $13.57 ($11.69, $15.46) | $11.35 ($10.32, $12.35) | $10.17 ($9.45, $10.88) | $9.66 ($9.1, $10.22) |
| **Non-prioritized** | ISL | $2.65 ($1.67, $3.63) | $3.0 ($2.53, $3.47) | $3.23 ($2.93, $3.53) | $3.09 ($2.87, $3.3) |
|  | CAB | $3.66 ($1.47, $5.86) | $4.5 ($3.38, $5.63) | $4.36 ($3.61, $5.11) | $4.55 ($4.0, $5.09) |
|  | LEN | $4.66 ($2.7, $6.63) | $4.57 ($3.64, $5.52) | $4.7 ($4.01, $5.37) | $4.58 ($4.11, $5.05) |
| **Zimbabwe** |  |  |  |  |  |
| **Risk-Prioritized** | ISL | $16.87 ($12.38, $21.18) | $16.31 ($13.91, $18.78) | $11.46 ($9.89, $13.1) | $9.98 ($8.62, $11.4) |
|  | CAB | $29.88 ($23.74, $36.3) | $22.88 ($19.93, $26.04) | $17.51 ($15.32, $19.82) | $15.01 ($13.27, $16.72) |
|  | LEN | $27.33 ($21.25, $33.83) | $23.44 ($20.49, $26.46) | $16.99 ($14.82, $19.19) | $14.44 ($12.84, $16.18) |
| **Non-prioritized** | ISL | $7.56 ($3.04, $12.42) | $6.08 ($3.71, $8.42) | $4.59 ($3.0, $6.22) | $5.2 ($4.16, $6.3) |
|  | CAB | $12.29 ($5.35, $18.96) | $10.12 ($6.84, $13.49) | $9.04 ($6.85, $11.22) | $7.98 ($6.43, $9.56) |
|  | LEN | $12.22 ($6.21, $18.42) | $8.96 ($5.72, $12.19) | $8.49 ($6.36, $10.66) | $8.68 ($7.19, $10.29) |
| **South Africa** |  |  |  |  |  |
| **Risk-Prioritized** | ISL | $55.48 ($50.26, $60.97) | $39.78 ($37.2, $42.37) | $32.87 ($31.08, $34.59) | $28.41 ($27.03, $29.78) |
|  | CAB | $105.85 ($95.99, $115.85) | $70.85 ($65.37, $76.54) | $54.94 ($51.32, $58.59) | $45.61 ($42.93, $48.36) |
|  | LEN | $98.75 ($88.77, $108.94) | $68.32 ($63.08, $73.42) | $52.96 ($49.25, $56.66) | $44.18 ($41.4, $46.99) |
| **Non-prioritized** | ISL | $11.21 ($5.73, $16.85) | $12.57 ($10.04, $15.13) | $12.54 ($10.86, $14.21) | $12.62 ($11.28, $13.9) |
|  | CAB | $29.43 ($18.98, $39.94) | $26.9 ($21.69, $32.46) | $25.18 ($21.7, $28.74) | $23.19 ($20.48, $25.94) |
|  | LEN | $29.69 ($19.31, $40.22) | $25.98 ($20.71, $31.19) | $22.53 ($18.97, $26.26) | $21.58 ($18.82, $24.45) |

| Table S8a. HIV infections averted at varying LA PrEP coverage levels |
| --- |

# Table S8: HIV infections averted and maximum annual price thresholds for LA PrEP assuming PrEP scale up among females only starting in 2035

| **Population** | **Product** | **5%** | **10%** | **15%** | **20%** |
| --- | --- | --- | --- | --- | --- |
| **Western Kenya** |  |  |  |  |  |
| **Risk-Prioritized** | ISL | 10.28 (9.69, 10.87) | 13.02 (12.29, 13.73) | 16.16 (15.55, 16.77) | 19.1 (18.49, 19.69) |
|  | CAB | 15.73 (15.05, 16.41) | 20.04 (19.36, 20.72) | 24.34 (23.68, 24.99) | 28.93 (28.28, 29.6) |
|  | LEN | 15.96 (15.3, 16.62) | 19.53 (18.84, 20.2) | 23.72 (23.12, 24.32) | 28.38 (27.86, 28.89) |
| **Non-prioritized** | ISL | 2.19 (1.44, 2.92) | 4.99 (4.23, 5.74) | 7.23 (6.46, 7.99) | 9.47 (8.79, 10.17) |
|  | CAB | 3.82 (2.95, 4.7) | 7.42 (6.64, 8.17) | 11.39 (10.63, 12.17) | 14.59 (13.86, 15.33) |
|  | LEN | 3.66 (2.94, 4.38) | 7.42 (6.69, 8.15) | 11.23 (10.46, 11.95) | 15.12 (14.42, 15.8) |
| **Zimbabwe** |  |  |  |  |  |
| **Risk-Prioritized** | ISL | 11.02 (8.25, 13.69) | 12.57 (10.0, 15.07) | 12.51 (9.62, 15.41) | 16.26 (14.11, 18.37) |
|  | CAB | 17.72 (14.93, 20.55) | 18.79 (15.96, 21.54) | 22.13 (19.53, 24.76) | 24.68 (21.89, 27.37) |
|  | LEN | 16.84 (13.96, 19.64) | 19.14 (16.37, 21.89) | 21.66 (19.01, 24.36) | 24.51 (21.54, 27.26) |
| **Non-prioritized** | ISL | 2.81 (-0.09, 5.66) | 3.05 (-0.33, 6.37) | 5.05 (1.9, 7.98) | 8.11 (5.09, 11.0) |
|  | CAB | 6.42 (2.87, 9.85) | 8.33 (5.1, 11.57) | 11.03 (7.77, 14.2) | 13.68 (10.34, 17.01) |
|  | LEN | 3.27 (0.17, 6.16) | 8.06 (5.27, 10.76) | 10.23 (6.88, 13.32) | 13.4 (10.03, 16.6) |
| **South Africa** |  |  |  |  |  |
| **Risk-Prioritized** | ISL | 7.4 (6.64, 8.18) | 7.97 (7.28, 8.64) | 9.54 (8.73, 10.35) | 10.42 (9.59, 11.26) |
|  | CAB | 13.23 (12.45, 13.99) | 15.43 (14.71, 16.16) | 16.87 (16.12, 17.63) | 19.41 (18.71, 20.14) |
|  | LEN | 12.96 (12.26, 13.65) | 14.7 (13.88, 15.5) | 16.52 (15.81, 17.27) | 18.69 (17.99, 19.38) |
| **Non-prioritized** | ISL | 1.25 (0.52, 1.99) | 3.34 (2.62, 4.03) | 3.99 (3.24, 4.71) | 5.71 (4.97, 6.49) |
|  | CAB | 3.73 (2.91, 4.57) | 5.65 (5.0, 6.31) | 8.22 (7.46, 9.0) | 11.19 (10.37, 12.01) |
|  | LEN | 2.81 (2.05, 3.56) | 5.38 (4.55, 6.21) | 8.4 (7.57, 9.23) | 11.37 (10.55, 12.17) |

| Table S8b. Maximum cost per person year at varying LA PrEP coverage levels |
| --- |

| **Population** | **Product** | **5%** | **10%** | **15%** | **20%** |
| --- | --- | --- | --- | --- | --- |
| **Western Kenya** |  |  |  |  |  |
| **Risk-Prioritized** | ISL | $13.35 ($12.2, $14.54) | $10.39 ($9.52, $11.23) | $9.39 ($8.91, $9.87) | $8.43 ($8.02, $8.85) |
|  | CAB | $19.28 ($16.32, $22.23) | $16.32 ($14.39, $18.25) | $13.91 ($12.6, $15.16) | $13.08 ($12.02, $14.1) |
|  | LEN | $19.81 ($17.27, $22.41) | $15.68 ($14.09, $17.23) | $13.23 ($12.19, $14.27) | $12.13 ($11.33, $12.94) |
| **Non-prioritized** | ISL | $4.4 ($2.8, $5.99) | $3.96 ($3.12, $4.78) | $4.34 ($3.84, $4.85) | $4.28 ($3.93, $4.65) |
|  | CAB | $6.53 ($2.75, $10.3) | $5.74 ($3.62, $7.77) | $6.1 ($4.81, $7.4) | $6.2 ($5.24, $7.2) |
|  | LEN | $5.63 ($2.35, $9.04) | $6.2 ($4.52, $7.88) | $6.61 ($5.43, $7.76) | $6.26 ($5.36, $7.14) |
| **Zimbabwe** |  |  |  |  |  |
| **Risk-Prioritized** | ISL | $25.26 ($18.81, $31.82) | $17.2 ($13.43, $21.04) | $12.08 ($9.42, $14.78) | $11.93 ($10.22, $13.74) |
|  | CAB | $39.41 ($30.59, $48.53) | $27.82 ($22.29, $33.55) | $21.1 ($17.64, $24.62) | $17.67 ($14.98, $20.53) |
|  | LEN | $36.69 ($28.63, $45.02) | $27.21 ($21.75, $32.92) | $20.9 ($17.34, $24.71) | $18.05 ($15.15, $21.0) |
| **Non-prioritized** | ISL | $9.37 ($2.23, $17.06) | $6.12 ($1.76, $10.46) | $6.37 ($3.75, $9.09) | $6.65 ($4.57, $8.78) |
|  | CAB | $18.24 ($6.12, $30.42) | $12.42 ($6.37, $18.58) | $10.63 ($6.84, $14.5) | $10.29 ($7.56, $13.17) |
|  | LEN | $12.02 ($2.38, $22.03) | $12.6 ($7.15, $17.94) | $11.35 ($8.01, $14.81) | $10.85 ($8.12, $13.72) |
| **South Africa** |  |  |  |  |  |
| **Risk-Prioritized** | ISL | $68.28 ($60.45, $75.95) | $41.04 ($36.54, $45.36) | $33.09 ($30.1, $36.2) | $26.68 ($24.33, $29.06) |
|  | CAB | $134.58 ($115.66, $153.53) | $81.86 ($72.84, $91.26) | $61.53 ($55.73, $67.48) | $51.95 ($47.27, $56.6) |
|  | LEN | $126.07 ($109.8, $142.33) | $74.89 ($65.98, $83.93) | $58.97 ($52.8, $65.32) | $49.51 ($44.95, $54.05) |
| **Non-prioritized** | ISL | $12.02 ($2.92, $21.17) | $17.02 ($12.7, $21.39) | $14.35 ($11.54, $17.29) | $16.14 ($13.81, $18.49) |
|  | CAB | $45.23 ($27.57, $62.8) | $35.8 ($26.97, $44.65) | $33.88 ($27.88, $40.2) | $32.13 ($27.75, $36.5) |
|  | LEN | $34.43 ($15.66, $53.58) | $32.29 ($23.44, $41.42) | $31.82 ($26.06, $37.67) | $31.34 ($26.63, $36.39) |

# Table S9: Costs and health outcomes of LA-PrEP interventions varying LA PrEP effectiveness, assuming 5% LA-PrEP scale up to all adults starting in 2030

| **Population** | **Product** | **Effectiveness** | **% of infections averted** | **% of deaths averted** | **Person Years on LA PrEP Per DALY Averted** | **Max. price per Person Year on LA PrEP ($)** |
| --- | --- | --- | --- | --- | --- | --- |
| **Zimbabwe** | | | | | | |
| Non-Prioritized | CAB | Low (76%) | 6.13 (2.58–9.57) | 2.59 (1.37–3.75) | 57.74 (38.29–122.34) | 10.31 (4.94–15.85) |
|  |  | High (97%) | 7.92 (4.59–11.32) | 3.11 (1.93–4.3) | 48.54 (33.19–90.05) | 12.26 (6.71–17.69) |
|  |  | Base (95%) | 7.34 (4.23–10.44) | 2.68 (1.5–3.79) | 50.77 (35.09–93.32) | 11.68 (6.37–16.86) |
| **Risk-Prioritized** |  | Low (76%) | 17.03 (14.39–19.67) | 5.81 (4.74–6.87) | 25.90 (21.07–33.40) | 22.96 (17.94–28.18) |
|  |  | High (97%) | 22.2 (19.41-24.96) | 7.95 (6.86-9.08) | 19.23 (16.34-23.04) | 30.89 (25.71-36.42) |
|  |  | Base (95%) | 19.86 (16.9–22.79) | 7.17 (6.07–8.3) | 21.0 (17.62–25.74) | 28.30 (23.14–33.6) |
| Non-Prioritized | MO | Low (56%) | 2.74 (-0.53–5.99) | 1.2 (0.03–2.31) | 110.01 (62.1–431.33) | 5.40 (1.34–9.7) |
|  |  | High (77%) | 6.65 (3.78–9.54) | 2.37 (1.35–3.38) | 59.99 (42.53–99.14) | 9.89 (5.98–13.77) |
|  |  | Base (67%) | 4.16 (1.25–7.03) | 1.94 (0.91–2.97) | 78.79 (51.78–176.72) | 7.52 (3.48–11.38) |
| **Risk-Prioritized** |  | Low (56%) | 11.3 (8.12-14.36) | 4.39 (3.23-5.52) | 34.54 (27.56-45.94) | 17.18 (13.01-21.47) |
|  |  | High (77%) | 15.9 (13.25-18.5) | 5.58 (4.49-6.68) | 26.98 (22.59-33.55) | 21.95 (17.8-26.16) |
|  |  | Base (67%) | 12.26 (9.4–15.05) | 4.38 (3.26–5.46) | 34.19 (27.61–44.37) | 17.35 (13.19–21.51) |
| Non-Prioritized | LEN | High (100%) | 7.53 (4.07–10.91) | 3.49 (2.33–4.71) | 49.7 (34.64–90.82) | 11.94 (6.53–17.22) |
|  |  | Base (95%) | 6.74 (3.43–9.9) | 3.07 (1.89–4.23) | 52.15 (35.45–101.16) | 11.46 (5.94–16.73) |
| Risk-Prioritized |  | High (100%) | 21.46 (19.05-23.81) | 8.47 (7.4-9.54) | 18.39 (15.85-21.81) | 32.27 (27.38-37.32) |
|  |  | Base (95%) | 20.97 (18.18–23.83) | 7.58 (6.4–8.83) | 20.29 (16.99–25.03) | 29.29 (24.08–34.75) |
|  |  |  |  |  |  |  |
| **Population** | **Product** | **Effectiveness** | **% of infections averted** | **% of deaths averted** | **Person Years on LA PrEP Per DALY Averted** | **Max. price per Person Year on LA PrEP ($)** |
| **South Africa** | | | | | | |
| Non-Prioritized | CAB | Low (76%) | 3.98 (3.14–4.79) | 1.57 (1.09–2.08) | 26.47 (19.48–40.56) | 25.96 (17.1–35.05) |
|  |  | High (97%) | 5.47 (4.61–6.32) | 2.05 (1.57–2.54) | 21.63 (17.12–29.8) | 31.81 (23.13–40.34) |
|  |  | Base (95%) | 5.48 (4.65–6.32) | 2.1 (1.62–2.59) | 21.26 (16.54–28.96) | 32.33 (23.58–40.96) |
|  |  | Low (76%) | 15.37 (14.59–16.15) | 6.39 (5.89–6.89) | 8.46 (7.65–9.48) | 81.16 (72.34–89.81) |
| Risk Prioritized |  | High (97%) | 22.71 (21.93–23.51) | 9.12 (8.68–9.57) | 6.05 (5.63–6.55) | 113.56 (105.39–121.78) |
|  |  | Base (95%) | 21.85 (20.97–22.7) | 8.88 (8.4–9.37) | 6.26 (5.82–6.79) | 109.68 (101.16–117.99) |
| Non-Prioritized | MO | Low (56%) | 2.04 (1.39–2.67) | 0.52 (0.15–0.89) | 74.68 (49.98–138.88) | 9.17 (4.76–13.54) |
|  |  | High (77%) | 3.36 (2.6–4.09) | 1.17 (0.79–1.54) | 47.48 (36.17–69.11) | 14.45 (9.92–18.91) |
|  |  | Base (67%) | 2.73 (2.0–3.43) | 0.91 (0.59–1.24) | 47.89 (37.28–65.49) | 14.29 (10.46–18.18) |
|  |  | Low (56%) | 9.37 (8.52–10.22) | 3.62 (3.29–3.98) | 15.02 (13.72–16.57) | 45.73 (41.42–50.01) |
|  |  | High (77%) | 15.28 (14.5–16.05) | 6.02 (5.67–6.37) | 9.51 (8.99–10.09) | 72.25 (68.16–76.24) |
|  |  | Base (67%) | 11.87 (11.1–12.58) | 4.87 (4.52–5.23) | 11.42 (10.68–12.25) | 60.19 (56.03–64.4) |
| Non-Prioritized | LEN | High (100%) | 4.78 (4.04–5.54) | 1.81 (1.27–2.35) | 25.36 (18.93–37.78) | 27.04 (18.0–36.23) |
|  |  | Base (95%) | 4.4 (3.65–5.19) | 1.71 (1.17–2.25) | 25.14 (18.65–37.95) | 27.37 (17.78–36.89) |
| Risk Prioritized |  | High (100%) | 24.38 (23.61–25.16) | 9.81 (9.3–10.29) | 5.94 (5.56–6.38) | 115.81 (108.05–123.75) |
|  |  | Base (95%) | 22.07 (21.3–22.85) | 8.83 (8.28–9.38) | 6.48 (6.0–7.05) | 105.99 (97.64–114.42) |
|  |  |  |  |  |  |  |
| **Population** | **Product** | **Effectiveness** | **% of infections averted** | **% of deaths averted** | **Person Years on LA PrEP Per DALY Averted** | **Max. price per Person Year on LA PrEP ($)** |
| **Western Kenya** | | | | | | |
| Non-Prioritized | CAB | Low (76%) | 4.78 (3.89–5.68) | 1.28 (0.83–1.73) | 183.39 (122.26–366.7) | 3.80 (1.9–5.72) |
|  |  | High (97%) | 6.35 (5.53–7.18) | 1.52 (1.13–1.88) | 135.24 (98.98–220.1) | 5.16 (3.31–7.07) |
|  |  | Base (95%) | 6.31 (5.56–7.07) | 1.3 (0.95–1.66) | 146.85 (105.22–250.69) | 4.76 (2.86–6.67) |
| Risk Prioritized |  | Low (76%) | 14.84 (14.19–15.46) | 3.13 (2.72–3.53) | 58.3 (50.36–68.69) | 11.95 (10.11–13.76) |
|  |  | High (97%) | 19.08 (18.38–19.77) | 4.2 (3.84–4.56) | 44.65 (40.05–50.56) | 15.59 (13.82–17.32) |
|  |  | Base (95%) | 18.72 (18.02–19.43) | 3.79 (3.43–4.14) | 46.72 (41.43–53.25) | 14.91 (12.97–16.73) |
| Non-Prioritized | MO | Low (56%) | 3.2 (2.49–3.9) | 0.58 (0.25–0.93) | 271.85 (209.6–387.9) | 2.57 (1.8–3.28) |
|  |  | High (77%) | 4.88 (4.16–5.59) | 1.13 (0.81–1.45) | 160.79 (137.47–193.79) | 4.34 (3.6–5.09) |
|  |  | Base (67%) | 4.25 (3.57–4.91) | 0.91 (0.6–1.23) | 203.79 (167.76–260.49) | 3.42 (2.67–4.17) |
| Risk Prioritized |  | Low (56%) | 10.56 (9.93–11.18) | 2.18 (1.88–2.51) | 82.68 (75.85–90.65) | 8.42 (7.66–9.18) |
|  |  | High (77%) | 14.87 (14.2–15.55) | 3.17 (2.89–3.45) | 56.53 (53.61–59.97) | 12.32 (11.64–13.02) |
|  |  | Base (67%) | 12.95 (12.23–13.67) | 2.66 (2.38–2.94) | 66.86 (62.33–72.14) | 10.43 (9.65–11.17) |
| Non-Prioritized | LEN | High (100%) | 19.81 (19.17–20.47) | 4.33 (3.96–4.69) | 43.42 (39.46–48.31) | 16.06 (14.46–17.67) |
|  |  | Base (95%) | 18.74 (18.14–19.37) | 3.99 (3.62–4.36) | 46.4 (42.03–51.64) | 15.0 (13.47–16.54) |
| Risk Prioritized |  | High (100%) | 6.43 (5.66–7.22) | 1.34 (0.92–1.75) | 132.06 (98.03–200.73) | 5.26 (3.42–7.11) |
|  |  | Base (95%) | 5.95 (5.21–6.69) | 1.26 (0.86–1.67) | 137.79 (104.86–198.08) | 5.05 (3.38–6.61) |
